# Influence of Complexation of Thiosemicarbazone Derivatives with Cu (II) Ions on Their Antitumor Activity against Melanoma Cells

**DOI:** 10.3390/ijms22063104

**Published:** 2021-03-18

**Authors:** Monika Pitucha, Agnieszka Korga-Plewko, Agnieszka Czylkowska, Bartłomiej Rogalewicz, Monika Drozd, Magdalena Iwan, Joanna Kubik, Ewelina Humeniuk, Grzegorz Adamczuk, Zbigniew Karczmarzyk, Emilia Fornal, Waldemar Wysocki, Paulina Bartnik

**Affiliations:** 1 Independent Radiopharmacy Unit, Faculty of Pharmacy, Medical University of Lublin, PL-20093 Lublin, Poland; monika.drozd@umlub.pl; 2 Independent Medical Biology Unit, Faculty of Pharmacy, Medical University of Lublin, PL-20093 Lublin, Poland; agnieszka.korga@umlub.pl (A.K.-P.); joanna.kubik@umlub.pl (J.K.); ewelinahumeniuk@umlub.pl (E.H.); grzegorzadamczuk@umlub.pl (G.A.); 3 Institute of General and Ecological Chemistry, Faculty of Chemistry, Lodz University of Technology, Zeromskiego 116, 90-924 Lodz, Poland; agnieszka.czylkowska@p.lodz.pl (A.C.); 211150@edu.p.lodz.pl (B.R.); 4 Chair and Department of Toxicology, Faculty of Pharmacy, Medical University of Lublin, PL-20093 Lublin, Poland; magda.iwan@umlub.pl; 5 Faculty of Science, Siedlce University of Natural Sciences and Humanities, 3 Maja 54, 08-110 Siedlce, Poland; zbigniew.karczmarzyk@uph.edu.pl (Z.K.); waldemar.wysocki@uph.edu.pl (W.W.); paulina.bartnik95@gmail.com (P.B.); 6 Chair and Department of Pathophysiology, Faculty of Medicine, Medical University of Lublin, PL-20090 Lublin, Poland; emilia.fornal@umlub.pl

**Keywords:** synthesis, thiosemicarbazone, X-ray investigation, copper(II) complex, TG-DTG techniques, melanoma, anticancer activity

## Abstract

A series of thiosemicarbazone derivatives was prepared and their anti-tumor activity in vitro was tested. The X-ray investigation performed for compounds T2, T3 and T5 confirmed the synthesis pathway and assumed molecular structures of analyzed thiosemicarbazones. The conformational preferences of the thiosemicarbazone system were characterized using theoretical calculations by AM1 method. Selected compounds were converted into complexes of Cu (II) ions. The effect of complexing on anti-tumor activity has been investigated. The copper(II) complexes, with Schiff bases T1, T10, T12, T13, and T16 have been synthesized and characterized by chemical and elemental analysis, FTIR spectroscopy and TGA method. Thermal properties of coordination compounds were studied using TG-DTG techniques under dry air atmosphere. G361, A375, and SK-MEL-28 human melanoma cells and BJ human normal fibroblast cells were treated with tested compounds and their cytotoxicity was evaluated with MTT test. The compounds with the most promising anti-tumour activity were then selected and their cytotoxicity was verified with cell cycle analysis and apoptosis/necrosis detection. Additionally, DNA damages in the form of a basic sites presence and the expression of oxidative stress and DNA damage response genes were evaluated. The obtained results indicate that complexation of thiosemicarbazone derivatives with Cu (II) ions improves their antitumor activity against melanoma cells. The observed cytotoxic effect is associated with DNA damage and G2/M phase of cell cycle arrest as well as disorders of the antioxidant enzymes expression.

## 1. Introduction

Thiosemicarbazones are a group of sulfur derivatives of semicarbazones that are obtained as a result of the condensation of appropriate aldehydes or ketones and thiosemicarbazides in an acidic environment. The structure of thiosemicarbazones has a significant influence on biological activity. Their antiviral [1], antidiabetic [2], antifungal, and antibacterial [3] activity is known.

There are also reports in the literature on the antitumor activity of thiosemicarbazone derivatives. Many substances are currently in clinical trials, for example: Triapine is a powerful anti-cancer drug in Phase 3 clinical trials. It is an inhibitor of ribonucleotide reductase with chelating properties [4]. It has been shown to form complex compounds with iron ions easily [5]. It has also been shown that the same compound, either in combination with cisplatin or gemcitabine, effectively inhibits the growth of cell proliferation of various types of cancer [6]. According to the literature, triapine shows great potential for use in the treatment of lung and ovarian cancers and some types of leukemia [7]. On the other hand, the compound labeled Dp44mT (di-2-pyridylketone-4,4-dimethyl-3-thiosemicarbazone) showed stronger antitumor effect than triapine and doxorubicin in in vitro studies, and was effective in the exposure of chemo- and radio-resistant cancer cell lines [8]. The potential of this molecule was to be used in the treatment of neuroblastoma, osteosarcoma and prostate cancer [9]. However, the compound showed strong cardiotoxic properties, therefore the search for safer analogues was started [10]. This led to the discovery of di-2-pyridylketone-4-cyclohexyl-4-methyl-3-thiosemicarbazone, which was found to be effective in the treatment of neuroblastoma [11]. Recently, there have been reports on the biological activity of thiosemicarbazone derivative complexes [12,13,14,15].

In our work, we have synthesized a new series of thiosemicarbazone derivatives. We subjected selected compounds to complexation reaction with Cu (II) ions. We investigated the effect of complexing on anti-cancer activity of new compounds.

## 2. Results and Discussion

### 2.1. Chemistry

**Scheme 1:**
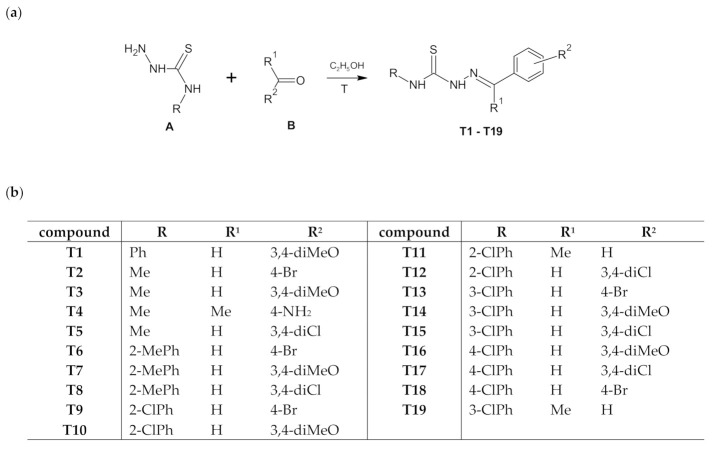
The route of synthesis of thiosemicarbazone derivatives (T1–T19) (**a**) and list of substituents (**b**).

Thiosemicarbazones can be classified as imine derivatives obtained by condensation reaction of thiosemicarbazide derivatives with aldehydes or ketones. The title compounds T1–T19 were obtained by the classic method using thiosemicarbazides: 4-methylthiosemicarbazide, 4-phenylthiosemicarbazide, 2-methylphenylthiosemicarbazide, 2-chlorophenylthiosemicarbazide, 3-chlorophenylthiosemicarbazide, 3-chlorophenylthiosemicarbazide, as substrates. The thiosemicarbazide was heated with the appropriate aldehyde or ketone (4-bromobenzaldehyde, 3,4-dimethoxybenzaldehyde, 3,4-dichlorobenzaldehyde, 4-aminoacetophenone, acetophenone) for 2.5h in ethanol. The reaction was carried out according to Scheme 1. The newly obtained compounds were identified by modern spectroscopic methods. For the newly obtained compounds, additional mass measurements were performed by high-performance liquid chromatography (Table S1). Compounds T1, T2, T14, T16 have been described earlier [16,17,18,19]. Compounds T15, T18, and T19 were obtained in the reactions of the corresponding hydrazone with an isothiocyanate [20,21].

**Figure 1:**
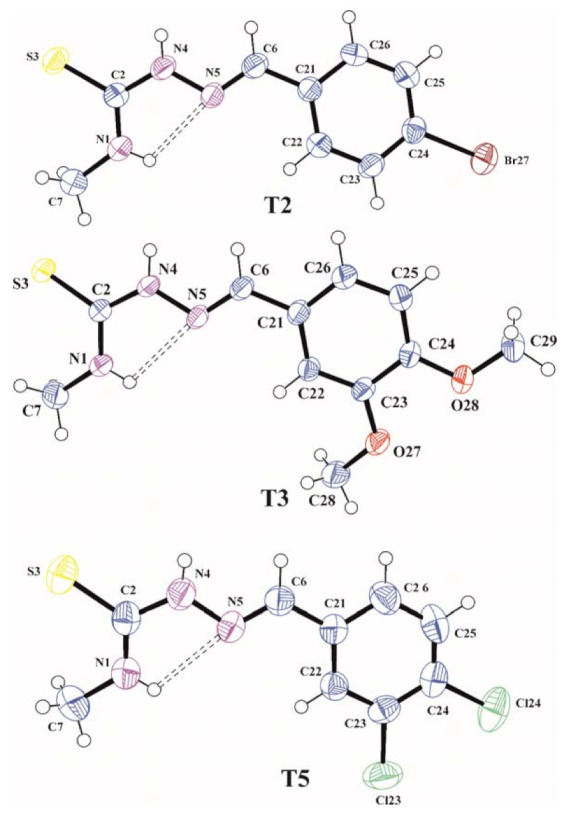
The molecular structures of T2, T3, and T5 with atom label ligand displacement ellipsoids (30% probability). The intramolecular hydrogen bond is shown as dashed line.

In order to confirm assumed synthesis pathway and molecular structures of the synthesized compounds the X-ray analysis for T2, T3, and T5 were performed. A view of these molecules in conformation observed in the crystal is shown in Figure 1.

The C2=S3, N4–N5, and N5=C6 bond lengths of 1.686(4), 1.373(4), and 1.266(5) Å in T2, 1.6896(13), 1.3809(14), and 1.2766(17) Å in T3 and 1.693(3), 1.375(3), and 1.272(3) Å in T5, respectively, are characteristic for those observed in other organic molecules containing thiosemicarbazone system [22,23] and confirming that the thione tautomeric form is observed in the crystalline state. In all analyzed molecules the thiosemicarbazone skeleton N1/C2/S3/N4/N5/C6 is slightly distorted from planarity with the largest deviation from the mean plane of 0.025(3) Å for N5 in T2, 0.0563(12) Å for N4 in T3 and 0.015(2) Å for N4 in T5. The torsion angles C7–N1–C2–N4, N1–C2–N4–N5, C2–N4–N5–C6, N4–N5–C6–C21 and N6–C6–C21–C22 of −179.5(4), −3.7(5), 179.2 (3), −178.7(3) and −7.3(6)° in T2, −177.01(14), 5.86(19), 178.25(13), −177.95(12) and 2.4(2)° in T3 and −174.5(3), −1.1(4), −179.8(2), −179.3(2) and −3.6(4)° in T5, respectively, show the *trans-cis-trans-trans-cis* conformation of the tiosemicarbazone chain in all molecules. The *cis* conformation on the C2–N4 bond favours the formation of N1–H1…N5 intramolecular hydrogen bond that stabilizes the conformation of the molecules observed in the crystal. The methoxy groups in T3 are located almost coplanar with the plane of the benzene ring and they have a *trans* conformation with respect to C23–C24 bond confirmed by the torsion angles C24–C23–O27–C28 and C23–C24–O28–C29 of −172.08(14) and −174.28(12)°, respectively. In all investigated molecules the benzene rings are planar within 0.005(4) Å in T2, 0.0121(14) Å in T3 and 0.010(3) Å in T5 and the planes of these rings are inclined with respect to the mean planes of thiosemicarbazone functions at an angle of 9.65(11), 8.08(4) and 4.03(7)° for T2, T3, and T5, respectively, which shows that all molecules as a whole are almost flat.

The intermolecular hydrogen bond N4–H4…S3 observed in the crystals of T2, T3, and T5 (Table 1), linking the molecules in molecular dimers, stabilized the tautomeric form occurring in the crystalline state.

It is worth noting that the pairs of these N4–H4…S3 hydrogen bonds in the crystal structure of T5 join the molecular chains parallel to Y crystallographic axis formed by molecules related by 2_1_ screw axis via N1–H1…S3 intermolecular hydrogen bonds. Similar molecular chains are observed in the crystal of T3, where molecules are linked via N1–H1…O27 hydrogen bonds. Moreover, in the crystal structure of T5, the benzene rings belonging to the inversion related molecules overlap each other with centroid-to-centroid separation of 3.8327(17) Å and the *π*…*π* distance of 3.6374(11) Å typical for the overlapping *π*-aromatic ring systems. The structural motifs formed by intermolecular hydrogen bonds in the crystal of T1, T2, and T3 are presented in Figure S1 (Supplementary Materials).

**Figure 2:**
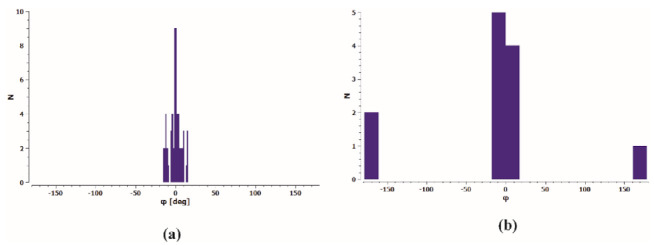
Histogram of torsion angle *φ* = N1–C2–N4–N5 in thiosemicarbazide system for (**a**) organic molecules and (**b**) ligands in Cu complexes deposited in CSD.

It can be assumed that the conformation of the thiosemicarbazone part in analysed molecules is one of the key factors related to the ability to coordinate the metal in the metalloorganic complexes. A search of the Cambridge Structural Database (CSD; ver. 2020.2.0,) [24,25] revealed 50 organic crystal structures and 59 molecules containing thiosemicarbazone system with N1-methyl and C6-phenyl substituents. The distribution of the torsion angle *φ* = N1–C2–N4–N5 calculated for all molecules is shown in Figure 2a. One can see that the *cis* conformation is observed in all found organic structures with the torsion angle φ in the range from −15.7 to +16.1°. Review of CSD exhibited also 7 metalloorganic crystal structures and 12 molecules of Cu complexes with a model thiosemicarbazone system as the ligands in these complexes. A certain tendency in torsion angle *φ* distribution (Figure 2b) can be seen. For the S-monodentate ligands (9 hits) conformation *cis* is preserved, while for S,N-bidentate ligands (3 hits) the *trans* conformation is observed.

**Figure 3:**
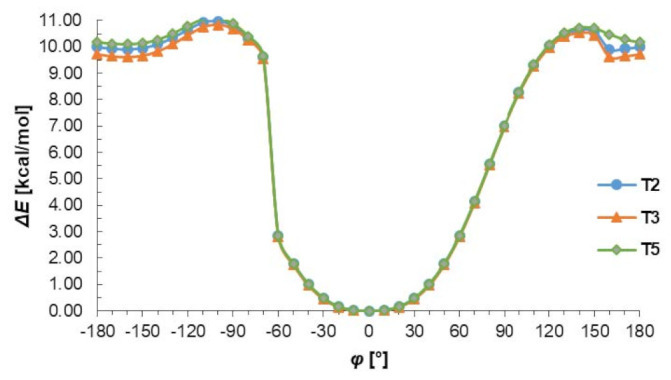
The energy effect of the free rotation for the torsion angle *φ* = N1–C2–N4–N5 calculated for **T2**, **T3**, and **T5** using the semiempirical AM1 method.

The conformational preferences of the T2, T3 and T4 were investigated by calculation of the energy effect of the free-rotation at the C2–N4 bond using the AM1 method. The energies of conformations were minimized, and all geometrical parameters optimized for each rotation with a 10° increment from −180 to 180° of torsion angle *φ* (Figure 3). As can be seen in Figure 3 two minima of energy are observed but the minimum energy corresponding to *cis* conformation (*φ* = 0°) is about 10 kcal/mol deeper than that of *trans* conformation (*φ* = ± 160°). This energy difference is due to the possibility of the N–H…N intramolecular hydrogen bond formation stabilizing the *cis* conformation. Changing from *cis* to *trans* conformation requires an energy input of about 11 kcal/mol, which may confirm the preference of thiosemicarbazone system to act as S-monodentate and does not exclude N,S-bidentate ligands in Cu complexes. It should be emphasized that the presented results of conformational analysis do not depend on the nature of substituents in benzene ring and are consistent with the data obtained from X-ray investigations.

Then, selected ligands from the group of thiosemicarbazone derivatives were subjected to complexation reaction with copper(II) ions. In order to identify the obtained compounds, FTIR spectra of the organic ligands and their copper(II) coordination compounds were recorded Figures S2–S6 (Supplementary Materials). The *ν*(NH) bands are visible in the spectra of ligands in the range 3334–3246 cm^−1^. However, they do not show a *ν*(S-H) band at ~ 2570 cm^−1^, but only strong bands for *ν*(NH), indicating that in the solid state Schiff bases are mainly in the tautomeric thione form. The δ(NH) modes are also present in the spectrum of all compounds. They are in the range: 1555–1492 cm^−1^ for free ligands and 1552–1499 cm^−1^ for coordination compounds. The bands of *ν*(N-N) appear in the range 1031–1010 cm^−1^ for Schiff bases and they do not move in the case of complexes. For uncoordinated ligands, the CH stretching vibrations are observed in the range 3145–2827 cm^−1^ and also the modes of β(CH) and γ(CH) in the ranges: 1277–1114 cm^−1^ and 799–703 cm^−1^, respectively. In the spectra of complexes, they are visible as follows: *ν*(CH) 3167–2828 cm^−1^, β(CH) 1278–1128 cm^−1^, and γ(CH) 774–718 cm^−1^. In all spectra of organic ligands there are several bands ascribed to *ν*(C=S). Due to the coordination of the sulphur atom with the metal ion, the spectra in this range become less vibrating. The disappearance of most of these bands in all complexes indicates formation of metal-sulphur bonds. For complexes Cu(T10)_2_Cl_2_ and Cu(T12)_2_Cl_2_ the bands of *ν*(CN) are in the range 1595–1579 cm^−1^ and they are in the similar range as for uncoordinated ligands. For these compounds, the organic ligands adopt only thione form. In the case of T1 and T16 two strong absorption bands of *ν*(CN) become one in the complexes. However, for Cu(T13)Cl_2_ one *ν*(CN) mode is shifted to higher wavenumbers (11 cm^−1^) as a result of coordination. Based on these data, it is likely that the Schiff bases (T1, T13 and T16) in these complexes coordinate via both the thiolate sulphur and azomethine nitrogen atoms [26,27]. Thus, it is clear from FTIR data that the Schiff bases bond with metal ions in different ways: as monodentate (T10, T12) and chelating (T1, T13, T16) ligands.

### 2.2. Thermal Decomposition

The thermal decompositions of described complexes have been studied in air by TG-DTG method. Pyrolysis of analyzed complexes in air is a multistage, overlapping process and complicated to interpret. Figures S7–S11 (Supplementary Materials) show thermal profiles of investigated complexes. Table 2 presents thermal decomposition results.

Cu(T1)Cl_2_ complex is stable up to 120 °C. The first step of decomposition takes place in the temperature range of 120–350 °C and is associated with partial destruction of an organic ligand (mass loss: found. 34.0%, calc. 33.45%). From this temperature up to 680 °C a total decomposition of complex (mass loss: found. 43.0%, calc. 42.83%) takes place. Final solid product of decomposition is copper(II) oxide.

Decomposition of Cu(T10)_2_Cl_2_ complex begins at 120 °C. In the range 120–360 °C the first step of pyrolysis of an organic ligand (mass loss: found. 45.0%, calc. 44.51%) occurs. In the temperature range of 360–720 °C there is a total destruction of complex (mass loss: found. 43.0%, calc. 42.67%). Final solid product appears above 720 °C.

Cu(T12)_2_Cl_2_ starts to decompose at 150 °C. Above this temperature decomposition of an organic ligand begins (mass loss: found. 37.5%, calc. 37.33%). Total destruction takes place in the temperature range of 260–720 °C with mass loss found 56.0% and calculated 55.20. Final solid product of thermolysis, copper(II) oxide, is created above 720 °C.

Decomposition of Cu(T13)Cl_2_ complex begins at 120 °C. Between 120 and 520 °C there is first mass loss on TG curve (mass loss: found. 33.5%, calc. 33.59%) which is connected with partial destruction of organic ligand. When the temperature rises (520–800 °C) further pyrolysis takes place (mass loss: found. 61.5%, calc. 50.60%). The process stops above 800 °C.

Cu(T16)Cl_2_ complex is stable up to 120 °C. In the temperature ranges: 120–360 °C and 360–780 °C partial and total degradation occurs. The mass losses connected with these processes are: found. 32.0%, calc. 31.01% and found. 47.5%, calc. 46.90%, respectively. A constant mass level appears above 780 °C.

According to the literature data, thiosemicarbazones are N, N, and S-donor ligands, therefore they show a high ability to form coordinate bonds with metal ions [28]. There is a theory that the antiproliferative activity of the group of thiosemicarbazones is due to this ability, while the strength of the activity of the coordination compounds formed is higher than that of the ligands [29,30,31,32].

### 2.3. Biological Assays

#### Cytotoxicity Analyses

Newly synthetized compounds were screened for anti cancer activity using in vitro methods. Three melanoma cel lines and normal human fibroblast were treated with thiosemicarbazone derivatives T1–T19 and Cu (II) complex of thiosemicarbazone derivatives CuT1, CuT10, CuT12, CuT13, and CuT16. For comparison, cells were treated with dacarbazine (DTIC)—a drug that is used in melanoma standard treatment.

**Figure 4:**
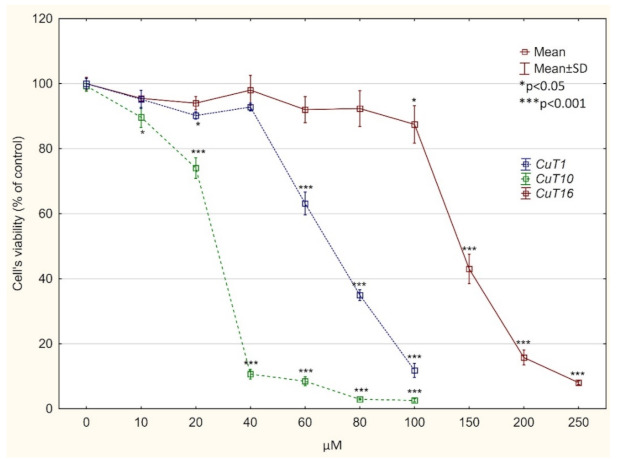
Viability of A375 cells, treated with CuT1, CuT10, and CuT16 compound or DMSO as vehicle in control culture for 24 h (determined by MTT test). Data presented as % of control culture viability (mean ± SD).

Compounds T1–T19 revealed weak cytotoxic activity towards melanoma cell lines or the compounds were toxic at the same level to melanoma cells and normal fibroblasts. However in case of Cu (II) complexes of obtain derivatives, analysis of IC_50_ values revealed that CuT1, CuT10, and CuT16 were toxic for A375 cells and at the same time less toxic for normal cells. CuT12 and CuT13 showed similar activity towards malignant and normal cell (IC_50_ values for selected derivatives and their corresponding Cu (II) complexes were summarized in Table 3). On the basis of the data received, CuT1, CuT10, and CuT16 have been choosen for further analysis using A375 cell precise cytotoxicity curves weres presented on Figure 4). It is worth noting that IC_50_ values obtained for selected compounds were lower than IC values for drug that is used in melanoma treatment—dacarbazine (DTIC).

**Figure 5:**
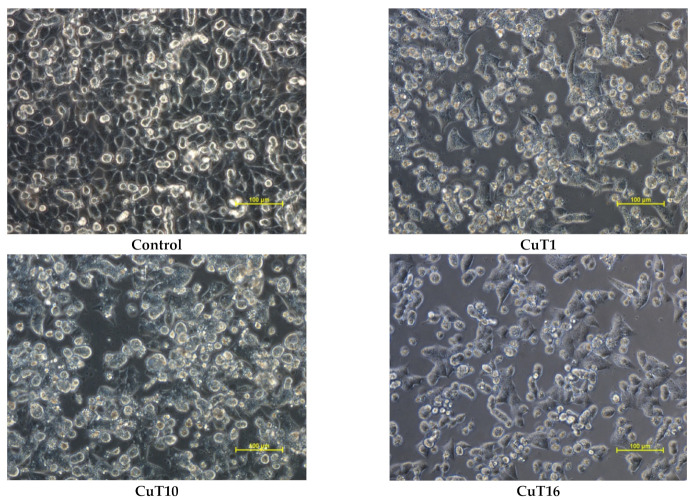
Morphology of A375 cells treated with CuT1, CuT10, and CuT16 compound in concentration corresponding to appropriate IC_50_ value (69.92, 26.05, and 144.60 µM, respectively) or DMSO as vehicle in control culture.

The MTT test results for selected compounds were verified by microscopic observations of cell morphology. The vehicle treated control cells revealed normal, epithelial-like morphology and were closely arranged and well adherent. Cells treated with CuT1, CuT10, and CuT16 in concentrations corresponding to IC_50_ values for 24 h became round and had poor adherence. A large part of the cells floated in the culture medium (Figure 5).

**Figure 6:**
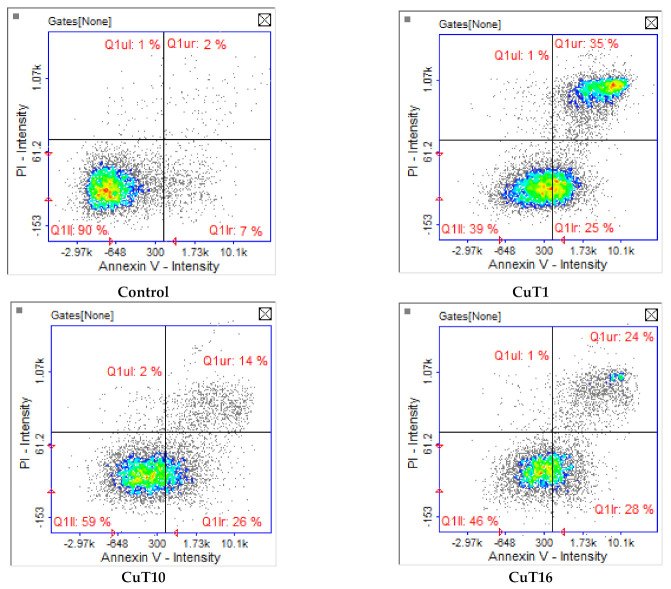
Apoptosis/necrosis detection by image cytometry. A375 cells were treated with CuT1, Cu10, CuT16 in concentrations corresponding to IC_50_ values (69.92, 26.05, and 144.60 µM respectively) or DMSO as vehicle in control cultures. The results show one representative experiment of three independently performed. Q1II—live, Q1Ir—early apoptotic, Q1ur—late apoptotic, and Q1uI—necrotic cells.

Apoptosis/necrosis analysis with image cytometry revealed that A375 cell treated with tested compounds were dying exclusively by apoptosis. The largest number of cells in the early and late stage of apoptosis after 24 h incubation was observed in case of CuT1 treatment. The fewest apoptotic cells were observed after treatment with CuT10, most of which were in the early phase (Figure 6).

Cytotoxicity studies have shown that A375 melanoma cell line was the most sensitive to the tested compounds. The identification of a specific feature that differs this cell line from the others might have contributed to the identification of the potential mechanism of action of the tested compounds. A375 and G361 have been described as low metastatic melanoma and SK-MEL-28 as highly metastatic melanoma cell line [33,34]. In accordance with Kim et al. results [35] A375 has intermediate migratory and invasive potential compared to G361 and SK-HEP-28. All three cell lines express mutant *B-Raf* gene. Analysis of published data revealed that A375 cells displaying low NAD(P)H: quinone oxidoreductase (NQO1) expression levels are sensitive to prooxidant agent (2,6-dichlorophenolindophenol) in contrast to G361 cells with high expression of NQO1 [36]. According to Sauviago et al. [37], who studied DNA repair signatures of the different melanoma cell lines, A375 is characterized by low DNA repair capacities in contrast to SK-MEL-28 cell line that has high DNA repair capacities.

**Figure 7:**
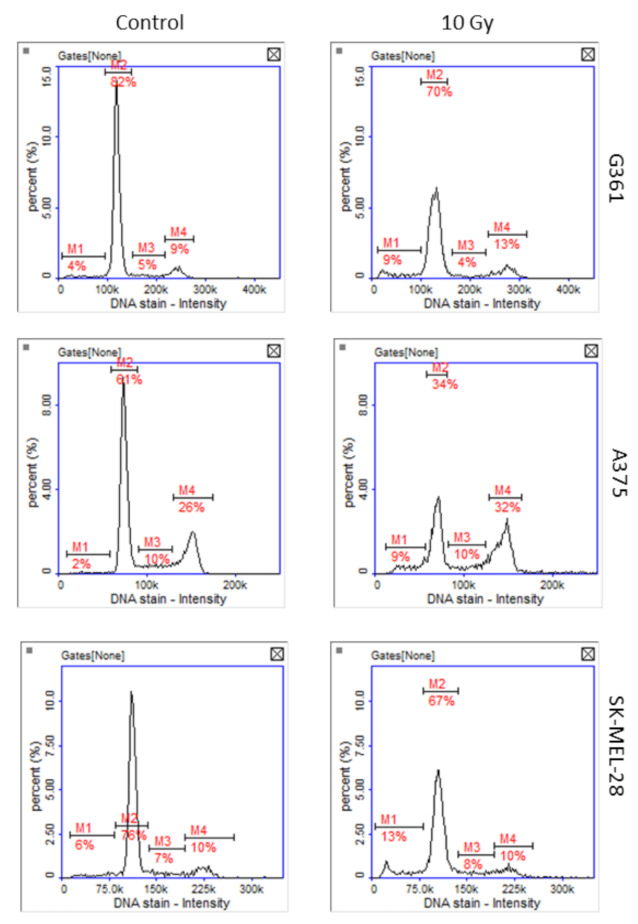
Representative histograms of cell cycle analysis obtained for G361, A375, and SK-MEL-28 melanoma cells 48 h after X-ray exposure (10 Gy, M1: subG1, M2: G1, M3: S, M4: G2/M phase).

Greater sensitivity of A375 to oxidative stress and DNA damage was confirmed by the unpublished results of our team’s research, in which cells of three melanoma lines were exposed to X-rays at a dose of 10 Gy. Cell cycle analysis of irradiated cells revealed that after 48 h only A375 cells were arrested in G2/M phase of the cell cycle (Figure 7).

The absorption of ionizing radiation by living cells can directly damage the molecules, causing chemical and biological changes or indirectly through water radiolysis products—free radicals that can damage nucleic acids, proteins, and lipids [38]. The majority of DNA damage after IR exposure is caused by IR-generated reactive oxygen species (ROS) [39].

**Figure 8:**
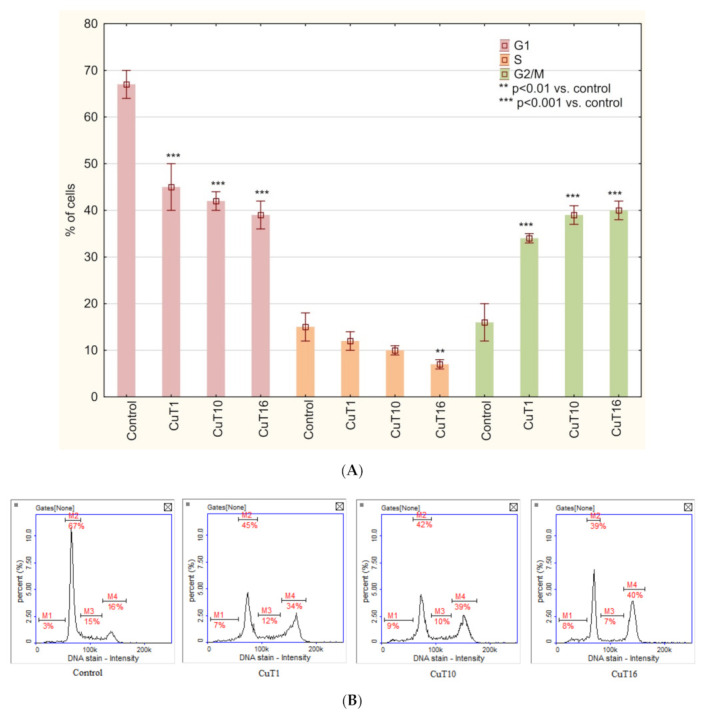
(**A**) Cell cycle analysis based on DAPI staining and image cytometry measurement. A375 cells were treated for 24 h with CuT1, CuT10, and CuT16 compounds in concentration corresponding to appropriate IC_50_ value (69.92, 26.05, and 144.60 µM respectively) or DMSO as vehicle in control cultures. Data presented as mean ± SD derived from three independent experiments. (**B**) Representative histograms (M1: subG1, M2: G1, M3: S, M4: G2/M phase).

Cell cycle analysis of A375 cells treated with tested compounds similarly revealed G2/M phase arrest (Figure 8).

**Figure 9:**
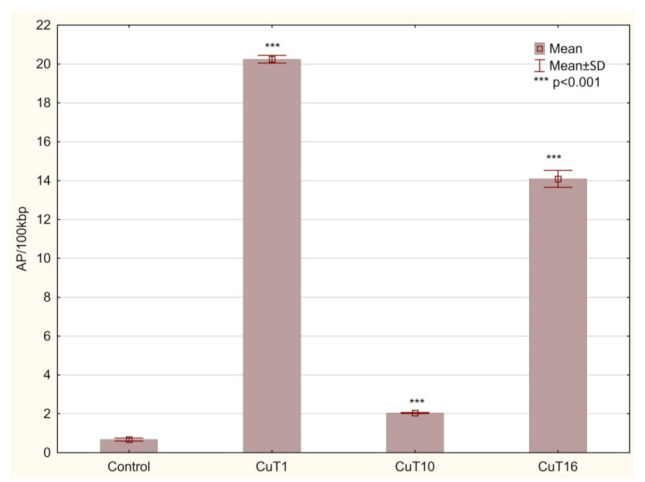
The amount of AP sites/100 kbp in DNA of A375 cells treated for 24 h with CuT1, CuT10, and CuT16 compound in concentration corresponding to appropriate IC50 value (69.92, 26.05, and 144.60 µM, respectively) or DMSO as vehicle in control cultures. Data presented as mean ± SD derived from three independent experiments.

G2/M phase arrest is connected to DNA repair attempt of damaged cells before mitosis phase. DNA damage agents (including prooxidative agents) can trigger a G2 checkpoint that results in a delay in the activation of cyclin B/Cdc2 kinase activity at the G2/M border [40]. The numbers of basic sites (AP sites) were measurement as a marker of DNA damage. AP sites can be formed by spontaneous depurination, but also occur as a result of oxidative damage. Hydroxyl radicals can attack the deoxyribose moiety and lead to the release of free bases, directly creating an AP site [41]. Moreover, these lesions are intermediates in base excision repair pathway where they are formed by DNA glycosylases [42]. In the DNA of cells treated with all three compounds, AP sites number was significantly elevated. However, in case of CuT10 it was only 2.043 ± 0.02 AP/100 kbp and for CuT1 and CuT16—20.24 ± 0.19 and 14.09 ± 0.44, respectively, vs. 0.67 ± 0.08 AP/100 kbp in DNA of control cells (Figure 9).

In the next step expression analysis of DNA damage and oxidative stress response genes was evaluated. Despite similar histograms of cell cycle analysis, there were not similar pattern of tested genes expression (Table 4). Only ATM transcript level was elevated in samples from cells treated with all three compounds. ATM is a key sensor of double strand breaks in DNA (DSBs) in G1 and G2 phase of cell cycle that signalizes this lesion presence to downstream effectors. ATM has been also hypothesized to play a vital role in the response to oxidative stress [40,43]. Thus, obtained results suggest that tested compounds may induce DNA damage probably resulted from oxidative stress.

The lower AP sites level in cells treated with CuT10 can possibly be connected with decreased expression of 8-oxoguanine-DNA glycosylase 1 (OGG1, RQ = 0.264 ± 0.071). OGG1, is the major enzyme for repairing 8-oxoguanine (8-oxoG), a critical mutagenic DNA lesion induced by reactive oxygen species. Human OGG1 excised the damaged base from an 8-oxoG, leaving AP sites as the major product [44].

Analysis of oxidative stress response genes revealed that in all samples superoxide dismutase 2 (SOD2) expression was elevated and catalase (CAT) and glutathione peroxidase 1 (GPX1) was downregulated, where the expression of GPX has decreased to an almost indeterminate level (Table 3). It is difficult to determine how the studied complexes of thiosemicarbazone derivatives affect the expression of these genes but regardless of the mechanism of this phenomenon, that expression profile may suggest that SOD is activated in response to oxidative stress (superoxide anion presence), but its product, hydrogen peroxide, cannot be effectively deactivated due to the absence of GPX (unable GSH–GSSG cycling) and lower level of catalase that mediates the breakdown of hydrogen peroxide to water and oxygen.

It was revealed that copper compounds can act by different mechanisms. One of them is generation of ROS by redox-cycling, that lead to an oxidative cell damage [45]. Obtained results suggest that anticancer activities of tested compounds result from oxidative damages of DNA connected with reduced ability to ROS neutralization.

## 3. Materials and Methods

### 3.1. Chemistry

All chemicals used for the synthesis were purchased from the companies Sigma-Aldrich, AlfaAesar, POCH and used without further purification. Melting points of obtained compounds were determined using Fisher-Johns block and presented without corrections. The ^1^H and ^l3^C NMR spectra were recorded on a Bruker Avance 600 spectrometer (Bruker BioSpin GmbH, Rheinstetten, Germany) in DMSO-d6. The chemical shifts are given in δ (ppm) scale using TMS as the standard reference. The attenuated total reflectance ATR-IR spectra were recorded over the range 4000–400 cm^−1^ on the Thermo Scientific Nicolet 6700 FTIR spectrophotometer. LC-QTOF HRMS measurements were performed using 1290 HPLC coupled to 6550 ifunnel QTOF LC/MS (Agilent Technologies, Santa Clara, CA, USA). MS data and MS spectrograms are presented in Supplementary Material (Table S1, Figures S2 and S3). The content of Cu (II) in solid complex was determined by the F-AAS spectrometer with a continuum source of light and using air/acetylene flame (Analityk Jena, contraAA 300, Jena, Germany). Absorbance was measured at analytical spectral line 324.7 nm. Limit of quantification was 0.04 mg/L. Solid sample was decomposed using the Anton Paar Multiwave 3000 closed system instrument. Mineralization was carried out for 45 min at 240 °C under pressure 60 bar. The contents of carbon, hydrogen and nitrogen were determined by a Vario micro company Elementar Analysensysteme GmbH. FTIR spectra were recorded with an IRTracer-100 Schimadzu Spectrometer (4000–600 cm^−1^) with accuracy of recording 1 cm^−1^ using KBr pellets. The thermolysis of compounds in air atmosphere were studied by TG-DTG techniques in the range of temperature 25–800 °C at a heating rate of 10 °C·min^−1^; TG and DTG curves were recorded on Netzsch TG 209 apparatus under air atmosphere v = 20 mL·min^−1^ using ceramic crucibles, and as a reference material ceramic crucibles were used.

#### 3.1.1. General Procedure for the Synthesis of Thiosemicarbazone Derivatives (T1–T19)

Thiosemicarbazone derivatives (T1–T19) were synthesized according to the general procedure by condensation of a thiosemicarbazide (0.01 mol) with an appropriate aldehyde or ketone (0.01 mol) in ethanol. Thiosemicarbazide was dissolved in ethanol while warm and the carbonyl compound dissolved in ethanol was added to the warm solution. The resulting mixture was heated to reflux for 1–2 h. Then, the reaction mixture was cooled to room temperature, the resulting precipitate was filtered off and, after drying, it was crystallized from ethanol.

#### 3.1.2. T1. 2-[(3,4-Dimethoxyphenyl)methylidene-*N*-phenyl- hydrazine-1-carbothioamide

C_16_H_17_N_3_O_2_S (315.39 g/mol), yield: 86%, m.p. 161–163°C. ^1^H NMR (DMSO-d_6_) δ: 3.80 (s, 3H, CH_3_), 3.83 (s, 3H, CH_3_), 6.99–7.58 (m, 8H, CH_aromat_), 8.09 (s, 1H, CH), 10.03 (s, 1H, NH), 11.74 (s, 1H, NH). ^13^C NMR (DMSO-d_6_) δ: 56, 56, 109, 111, 122, 125, 126, 127, 128, 139, 143, 149, 151, 176 [16]. FTIR spectra (KBr, cm^−1^): ν(NH) 3334, 3311; ν(CH) 3145–2834; ν(CN) 1598, 1573; δ(NH) 1540, 1507; β(CH) 1266, 1238, 1194, 1160, 1136; ν(NN) 1021; ν(CS) 976–862; γ(CH) 790, 755, 732.

#### 3.1.3. T2. 2-[(4-Bromophenyl)methylidene-*N*-methyl- hydrazine-1-carbothioamide

C_9_H_10_N_3_SBr (272.16 g/mol), yield: 81%, m.p. 181–183 °C. ^1^H NMR (DMSO-d_6_) δ: 3.02–3.03 (d, *J* = 4.8 Hz, 3H, CH_3_), 7.59–7.61 (m, 2H, CH_aromat_), 7.75–7.77 (m, 2H, CH_aromat_), 8.01 (s, 1H, CH), 8.57–8.58 (m, 1H, NH), 11.55 (s, 1H, NH). ^13^C NMR (DMSO-d_6_) δ: 31, 123, 129, 132, 134, 140, 178. IR cm^−1^: 3152, 2952, 1602 [17].

#### 3.1.4. T3. 2-[(3,4-Dimethoxyphenyl)methylidene-*N*-methyl- hydrazine-1-carbothioamide

C_11_H_15_N_3_O_2_S (253.32 g/mol), yield: 89%, m.p. 171–173 °C. ^1^H NMR (DMSO-d_6_) δ: 3.04–3.05 (d, *J =* 4.8 Hz, 3H, CH_3_), 3.79 (s, 3H, CH_3_), 3.83 (s, 3H, CH_3_), 6.96–6.98 (d, *J =* 8.4 Hz, 1H, CH_aromat_), 7.19–7.20 (d, *J =* 1.8 Hz*,* 1H, CH_aromat_), 7.46–7.47 (d, *J =* 1.8 Hz, 1H, CH_aromat_), 7.98 (s, 1H, CH), 8.42–8.43 (q, *J* = 4.2 Hz, 1H, NH), 11.39 (s, 1H, NH). ^13^C NMR (DMSO-d_6_) δ: 31, 56, 109, 111, 122, 127, 142, 149, 151, 177. IR cm^−1^: 3351, 2990, 1601 [46]. LC-QTOF HRMS (*m*/*z*): calculated monoisotopic mass: 253.0885, measured monoisotopic mass: 253.0889.

#### 3.1.5. T4. 2-[1-(4-Aminophenyl)ethylidene]-*N*-methyl-hydrazine-1-carbothioamide

C_10_H_14_N_4_S (222.31 g/mol), yield: 87%, m.p. 170–172 °C. ^1^H NMR (DMSO-d_6_) δ: 2.18 (s, 3H, CH_3_). 3.00 (s, 3H, CH_3_), 3.83 (s, 3H, CH_3_), 5.46 (s, 2H, CH_2_), 6.54–6.55 (q, *J =* 8.4 Hz, 2H, CH_aromat_), 7.63–7.64 (d, *J =* 8.4 Hz*, 2*H, CH_aromat_), 8.26–8.27 (d, *J* = 4.2 Hz, 1H, NH), 9.96 (s, 1H, NH). LC-QTOF HRMS (*m*/*z*): calculated monoisotopic mass: 222.0939, measured monoisotopic mass: 222.0944.

#### 3.1.6. T5. 2-[(3,4-Dichlorophenyl)methylidene]-*N*-methyl- hydrazine-1-carbothioamide

C_9_H_9_N_3_SCl_2_ (262.26 g/mol), yield: 80%, m.p. 192–194 °C. ^1^H NMR (DMSO-d_6_) δ: 3.02 (s, 3H, CH_3_), 7.66–7.99 (m, 3H, CH_aromat_), 8.21 (s, 1H, CH), 8.69 (q, *J* = 4.2 Hz, 1H, NH), 11.64 (s, 1H, NH). ^13^C NMR (DMSO-d_6_) δ: 31, 128, 131, 132, 135, 139, 178. IR cm^−1^: 3150, 2930, 1587 [18]. LC-QTOF HRMS (*m*/*z*): calculated monoisotopic mass: 260.9894, measured monoisotopic mass: 260.9896.

#### 3.1.7. T6. 2-[(4-Bromophenyl)methylidene]-*N*-(2-methylphenyl)hydrazine-1-carbothioamide

C_15_H_14_N_3_SBr (348,26 g/mol), yield: 85%, m.p. 178–180 °C. ^1^H NMR (DMSO-d_6_) δ: 2.23 (s, 3H, CH_3_), 7.21–7.28 (m, 4H, CH_aromat_), 7.61–7.88 (m, 4H, CH_aromat_), 8.10 (s, 1H, CH), 10.05 (s, 1H, NH), 11.58 (s, 1H, NH). IR cm^−1^: 3150, 2930, 1587. LC-QTOF HRMS (*m*/*z*): calculated monoisotopic mass: 347.0092, measured monoisotopic mass: 347.0096.

#### 3.1.8. T7. 2-[(3,4-Dimethoxyphenyl)methylidene]-*N*-(2-methylphenyl)hydrazine-1-carbothioamide

C_17_H_19_N_3_O_2_S (329.41 g/mol), yield: 85%, m.p. 205–206°C. ^1^H NMR (DMSO-d_6_) δ: 2.25 (s, 3H, CH_3_), 3.80 (s, 3H, CH_3_), 3.82 (s, 3H, CH_3_), 6.98–6.99 (m, 1H, CH_aromat_), 7.21–7.24 (m, 4H, CH_aromat_), 7.28–7.29 (m, 1H, CH_aromat_), 7.35–7.37 (m, 1H, CH_aromat_), 7.59–7.60 (m, 1H, CH_aromat_), 8.08 (s, 1H, CH), 9.87 (s, 1H, NH), 11.70 (s, 1H, NH). LC-QTOF HRMS (*m*/*z*): calculated monoisotopic mass: 329.1198, measured monoisotopic mass: 329.1202.

#### 3.1.9. T8. 2-[(3,4-Dichlorophenyl)methylidene]-*N*-(2-methylphenyl)hydrazine-1-carbothioamide

C_15_H_13_N_3_SCl_2_ (338,25 g/mol), yield: 84%, m.p. 200–202 °C. ^1^H NMR (DMSO-d_6_) δ: 2.24 (s, 3H, CH_3_), 7.22–7.30 (m, 4H, CH_aromat_), 7.65–7.67 (m, 1H, CH_aromat_), 7.77–7.78 (m, 1H, CH_aromat_), 7.79–8.10 (m, 1H, CH_aromat_), 8.37 (s, 1H, CH), 10.15 (s, 1H, NH), 11.94 (s, 1H, NH). LC-QTOF HRMS (*m*/*z*): calculated monoisotopic mass: 337.0207, measured monoisotopic mass: 337.0211.

#### 3.1.10. T9. 2-[(4-Bromophenyl)methylidene]-*N*-(2-chlorophenyl)hydrazine-1-carbothioamide

C_14_H_11_N_3_SBrCl (368,68 g/mol), yield: 86%, m.p. 209–211 °C. ^1^H NMR (DMSO-d_6_) δ: 7.31–7.86 (m, 8H, CH_aromat_), 8.13 (s, 1H, CH), 10.15 (s, 1H, NH), 12.04 (s, 1H, NH). ^13^C NMR (DMSO-d_6_) δ: 123, 127, 128, 129, 130, 131, 132, 133, 137, 142, 177. IR cm^−1^: 3137, 2970, 1738. LC-QTOF HRMS (*m*/*z*): calculated monoisotopic mass: 366.9546, measured monoisotopic mass: 366.9545.

#### 3.1.11. T10. 2-[(3,4-Dimethoxyphenyl)methylidene-*N*-(2-chlorophenyl)hydrazine-1-carbothioamide

C_16_H_16_N_3_O_2_SCl (349.84 g/mol), yield: 78%, m.p. 179–180°C. ^1^H NMR (DMSO-d_6_) δ: 3.80 (s, 3H, CH_3_), 3.82 (s, 3H, CH_3_), 7.00–7.86 (m, 7H, CH_aromat_), 8.10 (s, 1H, CH), 10.03 (s, 1H, NH), 11.94 (s, 1H, NH). ^13^C NMR (DMSO-d_6_) δ: 56, 109, 111, 123, 126, 127, 128, 129, 130, 136, 143, 149, 151, 176. FTIR spectra (KBr, cm^−1^): ν(NH) 3246; ν(CH) 3144–2827; ν(CN) 1595, 1579; δ(NH) 1552, 1507; β(CH) 1267, 1238, 1194, 1162, 1134; ν(NN) 1023; ν(CS) 978–858; γ(CH) 799, 753, 723. LC-QTOF HRMS (*m*/*z*): calculated monoisotopic mass: 349.0652, measured monoisotopic mass: 349.0653.

#### 3.1.12. T11. *N*-(2-Chlorophenyl)-2-(1-phenylethylidene)hydrazine-1-carbothioamide

C_15_H_14_N_3_SCl (303.80 g/mol), yield: 78%, m.p. 115–117°C. ^1^H NMR (DMSO-d_6_) δ: 2.41 (s, 3H, CH_3_), 7.28–8.01 (m, 8H, CH_aromat_), 10.12 (s, 1H, NH), 10.89 (s, 1H, NH). ^13^C NMR (DMSO-d_6_) δ: 125, 126, 127, 129, 136, 179. IR cm^−1^: 3599, 2970, 1594. LC-QTOF HRMS (*m*/*z*): calculated monoisotopic mass: 303.0597, measured monoisotopic mass: 303.0601.

#### 3.1.13. T12. 2-[(3,4-Dichlorophenyl)methylidene]-*N*-(2-chlorophenyl)hydrazine-1-carbothioamide

C_14_H_10_N_3_SCl_3_ (358.67 g/mol), yield: 81%, m.p. 210–211 °C. ^1^H NMR (DMSO-d_6_) δ: 7.33–8.11 (m, 7H, CH_aromat_), 8.34 (s, 1H, CH), 10.24 (s, 1H, NH), 12.11 (s, 1H, NH). ^13^C NMR (DMSO-d_6_) δ: 127, 128, 129, 131, 132, 135, 137, 140, 177. FTIR spectra (KBr, cm^−1^): ν(NH) 3315, 3258; ν(CH) 3140–2983; ν(CN) 1591; δ(NH) 1545, 1512; β(CH) 1265, 1204, 1197, 1114; ν(NN) 1031; ν(CS) 960–825; γ(CH) 787, 736, 723. LC-QTOF HRMS (*m*/*z*): calculated monoisotopic mass: 356.9661, measured monoisotopic mass: 356.9654.

#### 3.1.14. T13. 2-[(4-Bromophenyl)methylidene]-*N*-(3-chlorophenyl)hydrazine-1-carbothioamide

C_14_H_11_N_3_SBrCl (368.68 g/mol), yield: 86%, m.p. 162–164 °C. ^1^H NMR (DMSO-d_6_) δ: 7.26–7.89 (m, 8H, CH_aromat_), 8.14 (s, 1H, CH), 10.23 (s, 1H, NH), 12.02 (s, 1H, NH). ^13^C NMR (DMSO-d_6_) δ: 123, 124, 125, 130, 132, 132, 133, 141, 142, 176. FTIR spectra (KBr, cm^−1^): ν(NH) 3330; ν(CH) 3128–2972; ν(CN) 1578; δ(NH) 1536, 1499; β(CH) 1277, 1253, 1204, 1129; ν(NN) 1009; ν(CS) 964–867; γ(CH) 780, 746, 703. LC-QTOF HRMS (*m*/*z*): calculated monoisotopic mass: 366.9546, measured monoisotopic mass: 366.9551.

#### 3.1.15. T14. 2-[(4,5-Dimethoxyphenyl)methylidene-*N*-(3-chlorophenyl)hydrazine-1-carbothioamide

C_16_H_16_N_3_O_2_SCl (349.84 g/mol), yield: 80%, m.p. 180–182 °C. ^1^H NMR (DMSO-d_6_) δ: 3.81 (s, 3H, CH_3_), 3.84 (s, 3H, CH_3_), 6.99–7.79 (m, 7H, CH_aromat_), 8.11 (s, 1H, CH), 10.10 (s, 1H, NH), 11.88 (s, 1H, NH). ^13^C NMR (DMSO-d_6_) δ:. 56, 109, 111, 122, 124, 125, 126, 130, 132, 141, 144, 149, 151, 175. IR cm^−1^: 3140, 2834, 1597 [18].

#### 3.1.16. T15. *N*-(3-Chlorophenyl)-2-[(3,4-dichlorophenyl)methylidene]hydrazine-1-carbothioamide

C_14_H_10_N_3_SCl_2_ (358.67 g/mol), yield: 77%, m.p. 210–212 °C. ^1^H NMR (DMSO-d_6_) δ: 7.28–8.12 (m, 7H, CH_aromat_), 8.33 (s, 1H, CH), 10.30 (s, 1H, NH), 12.09 (s, 1H, NH) [20].

#### 3.1.17. T16. 2-[(4,5-Dimethoxyphenyl)methylidene-*N*-(4-chlorophenyl)hydrazine-1-carbothioamide

C_16_H_16_N_3_O_2_SCl (349.84 g/mol), yield: 76%, m.p. 200–202 °C. ^1^H NMR (DMSO-d_6_) δ: 3.80 (s, 3H, CH_3_), 3.83 (s, 3H, CH_3_), 6.99–7.64 (m, 7H, CH_aromat_), 8.10 (s, 1H, CH), 10.07 (s, 1H, NH), 11.83 (s, 1H, NH). ^13^C NMR (DMSO-d_6_) δ: 56, 109, 111, 122, 126, 128, 129, 138, 144, 149, 151, 176 [19]. FTIR spectra (KBr, cm^−1^): ν(NH) 3331, 3326; ν(CH) 3142–2828; ν(CN) 1599, 1574; δ(NH) 1545, 1505; β(CH) 1265, 1235, 1210, 1140; ν(NN) 1019; ν(CS) 976–832; γ(CH) 795, 768, 746, 716.

#### 3.1.18. T17. *N*-(4-Chlorophenyl)-2-[(3,4-dichlorophenyl)methylidene]hydrazine-1-carbothioamide

C_14_H_10_N_3_SCl_3_ (358.67 g/mol), yield: 83%, m.p. 202–204 °C, ^1^H NMR (DMSO-d_6_) δ: 7.43–8.12 (m, 7H, CH_aromat_), 8.34 (s, 1H, CH), 10.29 (s, 1H, NH), 12.05 (s, 1H, NH). ^13^C NMR (DMSO-d_6_) δ: 128, 129, 130, 131, 132, 135, 138, 141, 177. IR cm^−1^: 3122, 2970, 1589. LC-QTOF HRMS (*m*/*z*): calculated monoisotopic mass: 356.9661, measured monoisotopic mass: 356.9664.

#### 3.1.19. T18. 2-[(4-Bromophenyl)methylidene]-*N*-(4-chlorophenyl)hydrazine-1-carbothioamide

C_14_H_11_N_3_SBrCl (368.68 g/mol), yield: 89%, m.p. 182–184 °C. ^1^H NMR (DMSO-d_6_) δ: 7.42–7.89 (m, 8H, CH_aromat_), 8.13 (s, 1H, CH), 10.21 (s, 1H, NH), 11.98 (s, 1H, NH). ^13^C NMR (DMSO-d_6_) δ: 123, 128, 129, 130, 132, 133, 138, 142, 176, IR cm^−1^: 3128, 2971, 1589 [20].

#### 3.1.20. T19. *N*-(3-Chlorophenyl)-2-(1-phenylethylidene) hydrazine-1-carbothioamide

C_15_H_14_N_3_SCl (303,81 g/mol), yield: 80%, m.p. 162–165 °C. ^1^H NMR (DMSO-d_6_) δ: 2.39 (s, 3H, CH_3_), 7.26–7.69, 8,02 (m, 9H, CH_aromat_), 10.11 (s, 1H, NH), 10.77 (s, 1H, NH). ^13^C NMR (DMSO-d_6_) δ: 122, 123, 124, 130, 132, 141, 176. IR cm^−1^: 3138, 2970, 1586 [21].

### 3.2. X-ray Structure Determination

X-ray data of T2, T3 and T5 were collected on the KUMA Diffraction KM-4 CCD diffractometer; MoKα (λ = 0.71073 Å) radiation, ω scans, T = 296(2) K; crystal sizes 0.50 × 0.50 × 0.10 mm for T2, 0.50 × 0.50 × 0.30 mm for T3 and 0.50 × 0.40 × 0.30 mm for T5, absorption correction: multi-scan CrysAlisPro [47], Tmin/Tmax of 0.0054/1.0000, 0.7525/1.0000 and 0.2426/1.0000 for T2, T3, and T5, respectively. The structures were solved by direct methods using SHELXS97 [48] and refined by full-matrix least-squares with SHELXL-2014/7 [48]. The N-bound H atoms were located by difference Fourier synthesis and refined freely. The remaining H atoms were positioned geometrically and treated as riding on their parent C atoms with C-H distances of 0.93 Å (aromatic) and 0.96 Å (CH_3_). All H atoms were refined with isotropic displacement parameters taken as 1.5 times those of the respective parent atoms. All calculations were performed using WINGX version 1.64.05 package [49]. CCDC-2058475 for T2, 2058476 for T3, and 2058477 for T5 contain the supplementary crystallographic data for this paper. These data can be obtained free of charge at www.ccdc.cam.ac.uk/conts/retrieving.html (accessed on 15 Mar 2021) [or from the Cambridge Crystallographic Data Centre (CCDC), 12 Union Road, Cambridge CB2 1EZ, UK; fax: +44(0)-1223-336-033; email: deposit@ccdc.cam.ac.uk].

Crystal data of T2: C_9_H_10_N_3_SBr, *M* = 272.17, orthorhombic, space group *Pbca*, *a* = 13.580(15), *b* = 8.5494(8), *c* = 19.0926(18) Å, *V* = 2216.7(4) Å^3^, *Z* = 8, *d*_calc_ = 1.631 Mg m^−3, *F*^(000) = 1088, *μ*(MoK*α*) = 3.862 mm^−1^, *T* = 296K, 12,588 measured reflections (*θ* range 2.13–28.10°), 2431 unique reflections, final *R* = 0.049, *wR* = 0.129, *S* = 1.009 for 1713 reflections with *I* > 2*σ*(*I*).

Crystal data of T3: C_11_H_15_N3O2S, *M* = 253.32, monoclinic, space group *P*2_1_*/c*, *a* = 8.5409(7), *b* = 8.1329(5), *c* = 18.8922(12) Å, *β* = 102.099(7)°, *V* = 1283.15(16) Å^3^, *Z* = 4, *d*_calc_ = 1.311Mg m^−3, *F*^(000) = 536, *μ*(Mo K*α*) = 0.247 mm^−1^, *T* = 296K, 8182 measured reflections (*θ* range 2.20–27.78°), 2666 unique reflections, final *R* = 0.031, *wR*= 0.085, *S* = 1.045 for 2366 reflections with *I* > 2*σ*(*I*).

Crystal data of T5:C_9_H_9_N_3_SCl_2_, *M* = 262.15, monoclinic, space group *C*2/*c*, *a* = 12.8992(10), *b* = 10.0640(8), *c* = 18.4826(13) Å, *β* = 96.754(7), *V* = 2382.7(3) Å^3^, *Z* = 8, *d*_calc_ = 1.462 Mg m^−3, *F*^(000) = 1072, *μ*(MoK*α*) = 0.690 mm^−1^, *T* = 296K, 6897 measured reflections (*θ* range 2.22–28.06°), 2493 unique reflections, final *R* = 0.044, *wR*= 0.127, *S* = 1.066 for 1981 reflections with *I* > 2*σ*(*I*).

### 3.3. Theoretical Calculations

The conformational analysis for T2, T3 and T5 was performed using semiempirical AM1 method implemented in GAUSSIAN 03 [50]. The structures were fully optimized and the initial geometries were obtained from their crystallographic data.

### 3.4. General Procedure for the Synthesis of Cu (II) Complex of Thiosemicarbazone Derivatives

**Cu(T1)Cl_2_, Cu(T10)_2_Cl_2_, Cu(T12)_2_Cl_2_, Cu(T13)Cl_2_, Cu(T16)Cl_2_)** Synthesis of complexes was carried out in MeOH/EtOH (*v*/*v* = 1/1) mixture. In all cases molar ratio of organic ligand and copper(II) chloride dihydrate was 1:1. Total volume of reaction mixtures did not exceed 60 mL. After 6 h, green precipitates of Cu(T1)Cl_2_, Cu(T10)_2_Cl_2_, Cu(T13)Cl_2_, Cu(T16)Cl_2_, and yellow precipitate of Cu(T12)_2_Cl_2_ were filtered and washed three times with small amounts of MeOH/EtOH. Solid compounds were then dried in open air for several days and weighted.

**Cu(T1)Cl_2_ (C_16_H_17_N_3_SO_2_CuCl_2_)** (449.87 g/mol), yield (47%), anal. calculated (%): Cu, 14.12; C, 42.72; H, 3.81; N, 9.34. Found (%): Cu, 14.39; C, 42.50; H, 3.79; N, 8.85. FTIR spectra (KBr, cm^−1^): ν(NH) 3442, 3343; ν(CH) 3056–2833; ν(CN) 1590; δ(NH) 1500; β(CH) 1266, 1168, 1139; ν(NN) 1021; ν(CS) 853, 808; γ(CH) 764, 728.

**Cu(T10)_2_Cl_2_ (C_32_H_32_N_6_S_2_O_4_CuCl_4_)** (834.18 g/mol), yield (75%), anal. calculated (%): Cu, 7.62; C, 46.07; H, 3.87; N, 10.08. Found (%): Cu, 8.08; C, 46.25; H, 3.83; N, 9.66. FTIR spectra (KBr, cm^−1^): ν(NH) 3251; ν(CH) 3116–2828; ν(CN) 1597, 1576; δ(NH) 1555, 1508; β(CH) 1267, 1240; ν(NN) 1021; ν(CS) 939, 851; γ(CH) 738, 722.

**Cu(T12)_2_Cl_2_ (C_28_H_20_N_6_S_2_CuCl_8_)** (851.82 g/mol), yield (69%), anal. calculated (%): Cu, 7.46; C, 39.48; H, 2.37; N, 9.87. Found (%): Cu, 6.86; C, 39.69; H, 2.40; N, 9.71. FTIR spectra (KBr, cm^−1^): ν(NH) 3305, 3272; ν(CH) 3092, 2923; ν(CN) 1592; δ(NH) 1549, 1520; β(CH) 1278, 1205, 1197, 1128; ν(NN) 1031; ν(CS) 941, 900, 877; γ(CH) 745, 731.

**Cu(T13)Cl_2_ (C_14_H_11_N_3_SCuCl_3_Br)** (503.14 g/mol), yield (62%), anal. calculated (%): Cu, 12.63; C, 33.42; H, 2.20; N, 8.35. Found (%): Cu, 12.35; C, 33.26; H, 2.16; N, 8.23. FTIR spectra (KBr, cm^−1^): ν(NH) 3382, 3279; ν(CH) 3167–3067; ν(CN) 1589; δ(NH) 1537, 1505; β(CH) 1278, 1253, 1205, 1128; ν(NN) 1010; ν(CS) 959, 912, 871; γ(CH) 774, 745.

**Cu(T16)Cl_2_ (C_16_H_16_N_3_SO_2_CuCl_3_)** (484.31 g/mol), yield (35%), anal. calculated (%): Cu, 13.12; C, 39.68; H, 3.34; N, 8.68. Found (%): Cu, 13.15; C, 39.93; H, 3.30; N, 8.21. FTIR spectra (KBr, cm^−1^): ν(NH) 3424, 3265; ν(CH) 3096–2834; ν(CN) 1590; δ(NH) 1528, 1492; β(CH) 1268, 1177; ν(NN) 1020; ν(CS) 939, 858, 828; γ(CH) 768, 746, 718.

### 3.5. Biological Assays

#### 3.5.1. Cell Culturing

The evaluation of cytotoxicity of tested compounds was carried out on the G361, A375, and SK-MEL-28 human melanoma cells and BJ human normal fibroblast cells. The cell lines were obtained from American Type Culture Collection (ATCC, Manassas, VA, USA). The cells were cultured at 37 °C in the presence of 5% CO_2_ in atmosphere. The culture medium (Dulbecco’s Modified Eagle’s Medium for G361 and A375 and Eagle’s Minimum Essential Medium for SK-MEL-28 and BJ) was supplemented with 10% foetal bovine serum. The cells morphology was examined under inverted phase contrast microscope Nikon Eclipse Ti (Nikon, Tokyo, Japan). The authenticity of tested cell lines was verified by short tandem repeat (STR) genotyping in the Department of Forensic Medicine (Medical University of Lublin, Lublin, Poland).

#### 3.5.2. Cell Viability Assay

The cells were seeded into 96-well plates (in concentration: BJ, A375, and SK-MEL-28—1 × 10^5^ cells/mL, G361—1.5 × 10^5^ cells/mL) and cultured until they reach 70–80% confluency. Then the cells were incubated with tested compounds and dacarbazine in concentrations ranging from 10 to 500 µM or DMSO as vehicle in control cultures for next 24 h (max. DMSO concentration <0.5%). The compounds cytotoxicity was evaluated with the MTT colorimetric method based on the ability of viable cells to the transformation of tetrazolium salts (3-[4,5-dimethylthiazol-2-yl]-2,5-) diphenyltetrazolium bromide, MTT) to purple formazan, by cellular dehydrogenases. After 24-h-incubation cell cultures were supplemented with 20 µL of 5 mg/mL MTT (Thermofisher, Waltham, MA, USA) stock in PBS (Corning, NY, USA), and the incubation was continued for 4 h at 37 °C. Next, the medium with MTT was removed, and the formed crystals were dissolved in 200 µL of DMSO (dimethyl sulfoxide, Avantor, Gliwice, Poland). The solution absorbency was measured at 570 nm, using a PowerWave™ xs microplate spectrophotometer (BioTek Instruments, Winooski, VT, USA). The experiment was performed three times with three replicates for each concentration of tested compounds. IC_50_ values were determined using the AAT Bioquest IC_50_ calculator [51]. On the basis of obtained IC_50_ values for normal and malignant cells, A375 cell line and three tested compounds (CuT1, Cu10, CuT16) were chosen for further investigation.

#### 3.5.3. Cell Cycle Assay

Cell-cycle assay was performed using NC 3000 system (ChemoMetec, Lillerød, Denmark) according to manufacturer’s protocol for the two-step cell cycle analysis. The A375 cells were seeded in 12-well plates at density of 1 × 10^5^ cells/mL and after 70–80% confluency achievement the cells were treated with CuT1, Cu10, CuT16 in concentrations corresponding to IC_50_ values or DMSO as vehicle in control cultures and analysed after 24h incubation. The cells were washed with PBS (Corning, New York, NY, USA), incubated with lysis buffer (Solution 10) supplemented with 10 µg/mL of DAPI for 5 min at 37 °C. After addition of stabilization buffer (Solution 11) the samples were analysed. Fluorescence signal was quantified using a NucleoCounter^®^ NC-3000™ image cytometer (ChemoMetec USA Inc., Lillerød, Denmark). Data were derived from three independent experiments with three replicates for each concentration of tested compound.

#### 3.5.4. Cell Apoptosis Assay

Cell apoptosis assay was performed using NC 3000 system (ChemoMetec USA Inc., Lillerød, Denmark) according to the manufacturer’s protocol for Annexin V Assay. The A375 cells were seeded in 6-well culture plates at a density 1 × 10^5^ cells/mL and after 70–80% confluency achievement the cells were treated with CuT1, Cu10, CuT16 in concentrations corresponding to IC_50_ values or DMSO as vehicle in control cultures and analysed after 24h incubation. The cells were dissociated into single-cell suspensions in PBS (Corning, New York, NY, USA) and app. 4 × 10^5^ cells were resuspended in 100µl of Annexin V binding buffer and incubated with Annexin V—CF488A conjugate and Hoechst 33,342 for 15 min at 37 °C. Next, the cells were washed with Annexin V binding buffer and the cell pellets were resuspended in 100 mL of Annexin V binding buffer supplemented with 10 µg/mL of propidium iodide (PI) and Annexin V Assay was performed immediately. Cellular fluorescence was quantified using a NucleoCounter^®^ NC-3000™ image cytometer (ChemoMetec USA Inc., Lillerød, Denmark). Data were derived from three independent experiments with three replicates for each concentration of tested compounds.

#### 3.5.5. DNA Damage Evaluation

DNA damage was evaluated on the basis of a basic (AP) sites presence, that arose in response to oxidative damage or as an intermediate in base excision repair pathway. The A375 cells were seeded in 6-well culture plates at a density 1 × 10^5^ cells/mL and after 70–80% confluency achievement the cells were treated with CuT1, Cu10, CuT16 in concentrations corresponding to IC_50_ values or DMSO as vehicle in control cultures. After 24 h of incubation genomic DNA was isolated using Syngen DNA Mini Kit (Syngen Biotech, Wroclaw, Poland) according to the manufacturer’s instruction. The concentrations of isolated DNA for all samples were equalized to 100 µg/mL. The measurements of the number of AP sites were evaluated with DNA Damage Quantification Kit (Dojindo, Japan) according to the manufacturer’s instruction. The DNA was treated with Aldehyde Reactive Probe (ARP) to tag all of the AP sites with a biotin residue. Then all of the AP sites were quantified using avidin—biotin assay. The coloured product of horseradish peroxidase conjugated to the avidin was detected at 650 nm using a PowerWave™ xs microplate spectrophotometer (BioTek Instruments, Winooski, VT, USA).

#### 3.5.6. Gene Expression Evaluation

The expression level of genes connected with DNA repair and oxidative stress response was evaluated with quantitative real-time PCR (qRT-PCR) method. The A375 cells were seeded in 6-well culture plates at a density 1 × 10^5^ cells/mL and after 70–80% confluency achievement the cells were treated with CuT1, Cu10, CuT16 in concentrations corresponding to IC_50_ values or DMSO as vehicle in control cultures. After 24h of incubation total RNA was isolated using the Syngen Blood/Cell RNA Mini Kit (Syngen Biotech, Wroclaw, Poland). All samples were transcribed using NG dART RT-PCR reagents (EURx, Gdansk, Poland) according to the manufacturer’s instructions. The relative mRNA expression level was determined by relative quantification method (ΔΔCt) using 18S ribosomal N5 (RNA18SN5) and beta-actin (ACTB) as endogenous controls. The reference genes were selected on the basis of our preliminary studies, where *RNA18SN*5 and *ACTB* remained unaffected by the experimental conditions. The PCR was conducted in triplicate using Applied Biosystems^®^ 7500 Fast Real-Time PCR system (Applied Biosystems, Foster City, CA, USA) and SG qPCR Master Mix (2×) (EURx, Gdansk, Poland) in accordance with the manufacturer’s protocol. Data was presented and analysed as RQ values (RQ = 2^−ΔΔCt^). The genes and sequences of used primers were listed in Table 5.

#### 3.5.7. X-ray Radiation

G361, A375 and SK-MEL-28 were seeded in 6-well plates at following density: A375 and SK-MEL-28—1 × 10^5^ cells/mL, G361—1.5 × 10^5^ cells/mL and were irradiated using the RS-2000 Biological Research Irradiator (Rad Source Technologies, Buford, GE, USA). In the studies radiation at the dose of 10 Gy was used. X-ray treated cells were incubated for further 48 h under standard culturing conditions (characterised in Cells culturing section). Control cells that had not been exposed to X-rays were also cultured for 48 h. To compare all three cell lines responses, cell cycle analysis was conducted using NC 3000 system as described above.

#### 3.5.8. Statistical Analysis

Statistical analysis was performer using STATISTICA 13 software (StatSoft, Krakow, Poland). Comparison of values was performed by one-way analysis of variance (ANOVA) and post hoc multiple comparisons with Tukey’s honest significant difference test (Tukey’s HSD test). The data were calculated as mean ± SD. If the *p*-value was under 0.05, the results were considered statistically significant.

## 4. Conclusions

A number of thiosemicarbazone derivatives have been prepared as potential anti-cancer agents. X-ray examinations carried out for T2, T3 and T5 confirmed the synthesis pathway, the assumed molecular structures and showed that the tautomeric form of thione is the dominant crystal. All the molecules as a whole are almost flat and their conformation is strongly dependent on the intramolecular N–H… N hydrogen bond in the thiosemicarbazone part. In the next step, five Cu (II) thiosemicarbazone complexes have been successfully synthesized and described. The way of complexation has been established using adequate analytical techniques to confirm their formulae.

In vitro studies of the anti-tumor activity of ligands and complexes showed that the complexation of thiosemicarbazone derivatives with Cu (II) ions improves their anti-tumor activity against melanoma cells. The observed cytotoxic effect is related to DNA damage and the G2/M phase of cell cycle arrest and disturbed expression of antioxidant enzymes. Presented results are outstanding and highlight the potential of complexation of biologically active compounds. Further studies on this topic have to be performed, as complexation is a powerful tool in the search for more efficient and reliable drugs.

## Figures and Tables

**Table 1:** Hydrogen-bond geometry (Å, °).

Compound	D–H…A	D–H	H…A	D…A	D–H…A
**T2**					
	N1–H1…N5	0.78(5)	2.26(6)	2.646(5)	111(5)
	N4–H4…S3 *^i^*	0.80(4)	2.63(4)	3.416(3)	167(4)
	*i* = 1 − *x*, −*y*, 1 − *z*;				
**T3**					
	N1–H1…N5	0.812(18)	2.316(19)	2.6743(17)	107.5(16)
	N1–H1…O27 *^i^*	0.812(18)	2.521(18)	3.1757(17)	138.7(14)
	N4–H4…S3 *^ii^*	0.855(18)	2.569(18)	3.4098(12)	168.0(15)
	*i* = 1 − *x*, −1/2 + *y*, 1/2 − *z*; *ii* = 2 − *x*, −*y*, −*z*;				
**T5**					
	N1–H1…N5	0.76(4)	2.34(4)	2.649(3)	106(3)
	N1–H1…S3 *^i^*	0.76(4)	2.86(4)	3.522(2)	146(3)
	N4–H4…S3 *^ii^*	0.78(4)	2.70(4)	3.447(2)	160(4)
	*i* = 1/2−*x*, −1/2 + *y*, 3/2−*z*; *ii* = −*x*, *y*, 2 − *z*;				

**Table 2:** TG-DTG analysis data for decomposition processes of copper(II) complexes.

Compound		Range of Decomposition (℃)	Mass Loss (%)		Intermediate Solid Product or Residue
			Found	Calc.	
**Cu(T1)Cl_2_**	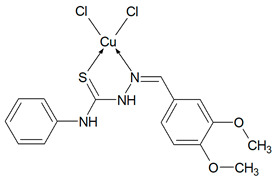	**120–350**	34.0	33.45	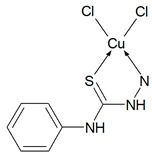
		350–680	43.0	42.83	CuO
Cu(T10)_2_Cl_2_	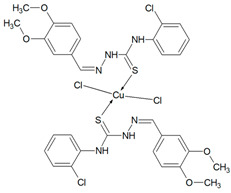	120–360	45.0	44.51	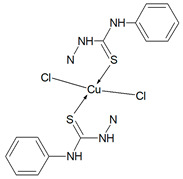
		360–720	43.0	42.67	CuO
Cu(T12)_2_Cl_2_	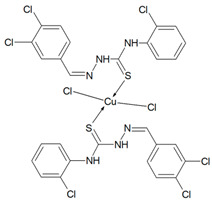	150–260	37.5	37.33	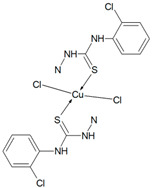
		260–720	56.0	55.20	CuO
Cu(T13)Cl_2_	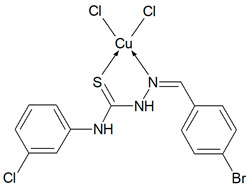	120–520	33.5	33.59	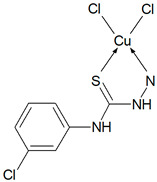
		520–800	61.5	50.60	CuO + volatile copper halide derivatives
Cu(T16)Cl_2_	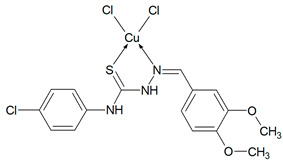	120–360	32.0	31.01	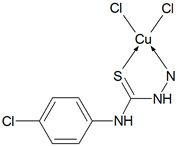
		360–780	47.5	46.90	CuO

**Table 3:** IC_50_ values obtained for tested compounds and dacarbazine (DTIC) based on 3-(4,5-dimethylthiazol-2-yl)-2,5-diphenyltetrazolium bromide test (MTT test) results after 24 h treatment of melanoma and normal fibroblast cell lines.

			IC_50_ [µM]	
	BJ	G361	A375	SK-MEL-28
**T1**	357.64 ± 7.85	488.07 ± 1.66	320.99 ± 2.85	>500
**CuT1**	257.12 ± 4.45	135.64 ± 7.01	69.92 ± 1.87	129.51 ± 3.98
**T10**	402.17 ± 12.98	370.17 ± 9.34	201.77 ± 4.87	<500
**CuT10**	202.88 ± 8.67	251.19 ± 11.08	26.05 ± 1.75	132.98 ± 8.34
**T12**	128.87 ± 7.09	177.35 ± 4.09	111.76 ± 12.75	99.64 ± 5.76
**CuT12**	42.87 ± 1.17	157.17 ± 2.75	40.49 ± 0.98	46.13 ± 2.74
**T13**	412.75 ± 3.23	395.75 ± 6.74	388.64 ± 7.06	398.86 ± 4.64
**CuT13**	130.18 ± 3.45	148.07 ± 9.77	109.78 ± 2.95	118.60 ± 9.75
**T16**	>500	>500	>500	>500
**CuT16**	450.09 ± 11.74	>500	144.60 ± 19.85	478.31 ± 11.02
**DTIC**	>500	425.98 ± 4.74	412.77 ± 7.08	370.12 ± 9.46

**Table 4:** The results of mRNA gene expression. Significantly changed mean RQ levels are marked with a color scale.

Gene symbol	Control		CuT1		CuT10		CuT16		Scale (RQ)	
	Mean RQ	SD	Mean RQ	SD	Mean RQ	SD	Mean RQ	SD		
*DNA damage response genes*										
*ERCC*	1.001	0.066	0.401	0.010	1.215	0.155	0.267	0.036	>2.00	
*MGMT*	1.007	0.147	0.715	0.088	0.448	0.024	0.361	0.053	1.51–1.99	
*OGG1*	1.011	0.185	0.902	0.022	0.264	0.071	0.952	0.160	1.11–1.50	
*MLH1*	1.002	0.078	1.001	0.163	0.337	0.057	0.929	0.068	0.91–1.10	
*PARP1*	1.011	0.193	0.908	0.056	0.342	0.108	0.640	0.069	0.51–0.90	
*ATM*	1.005	0.127	1.452	0.102	2.038	0.166	2.724	0.295	0.31–0.5	
*XPC*	0.951	0.044	1.038	0.087	1.456	0.112	1.438	0.244	<0.31	
*MSH2*	1.003	0.094	0.973	0.100	1.337	0.436	0.936	0.093		
*oxidative stress response genes*										
*CAT*	1.003	0.101	0.773	0.044	0.801	0.065	0.387	0.056		
*GPX2*	1.010	0.184	0.000	0.000	0.022	0.000	0.022	0.002		
*SOD2*	1.009	0.173	1.510	0.114	1.430	0.082	1.393	0.151		

**Table 5:** Primers used in gene expression evaluation.

Gene Symbol	Protein Name	Forward Sequence (5′→3′)	Reverse Sequence (5′→3′)
*ERCC*	ERCC Excision Repair 1	CTCGGAGTTTTGTGGGGGAC	CACTGGCGTCTACGTTCTCA
*MGMT*	O-6-methylguanine-DNA methyltransferase	ACCGTTTGCGACTTGGTACT	TGCTCACAACCAGACAGCTC
*OGG1*	8-oxoguanine DNA glycosylase	CCTGTGGGGACCTTATGCTG	TGTGAATCCCCTCTCCCGAT
*MLH1*	mutL homolog 1	GCACCGGGATCAGGAAAGAA	GCCTCACCTCGAAAGCCATA
*PARP1*	poly(ADP-ribose) polymerase 1	CCCCACGACTTTGGGATGAA	AGACTGTAGGCCACCTCGAT
*ATM*	ataxia telangiectasia mutated protein kinase	GCCGCGGTTGATACTACTTTG	GCAGCAGGGTGACAATAAACA
*XPC*	xeroderma pigmentosum group C-complementing protein	GCGAAGTGGAATTTGCCCAG	TTGGCCTTGGATTTCTGGCT
*MSH2*	MutS homolog 2	CAGGAGGTGAGGAGGTTTCG	CCGTGCGCCGTATAGAAGTC
*SOD2*	superoxide dismutase 2	CTTCAGGGTGGTATGGCTGT	TGGCCAGACCTTAATGTTCC
*GPX1*	glutathione peroxidase 1	TTGACATCGAGCCTGACATC	ACTGGGATCAACAGGACCAG
*CAT*	catalase	AGCTTAGCGTTCATCCGTGT	TCCAATCATCCGTCAAAACA
*RNA18SN5*	18S ribosomal N5	GAAACTGCGAATGGCTCATTAAA	CACAGTTATCCAAGTGGGAGAGG
*ACTB*	beta-actin	AGAGCTACGAGCTGCCTGAC	AGCACTGTGTTGGCGTACAG

## Data Availability

Not applicable.

## Supplementary Materials

Supplementary materials can be found at https://www.mdpi.com/1422-0067/22/6/3104/s1.

## Abbreviations

MDPI	Multidisciplinary Digital Publishing Institute
DOAJ	Directory of open access journals
TLA	Three letter acronym
LD	linear dichroism
