# Supplementary material for: Influence of Complexation of Thiosemicarbazone Derivatives with Cu (II) Ions on Their Antitumor Activity against Melanoma Cells
Source: Int J Mol Sci. 2021 Mar 18;22(6):3104. doi: 10.3390/ijms22063104 (PMC8002893; doi:10.3390/ijms22063104)
Supplement: Supplementary file 1 [file ijms-22-03104-s001.pdf]

<u>**Supplementary material for publication on line**</u>

Influence of complexation of thiosemicarbazone derivatives with Cu (II) ions on their antitumor activity against melanoma cells

Monika Pitucha [1,*], Agnieszka Korga-Plewko[2], Agnieszka Czylkowska[3], Bartłomiej Rogalewicz[3], Monika Drozd[1] Magdalena Iwan [4], Joanna Kubik[2], Ewelina Humeniuk[2], Grzegorz Adamczuk[2], Zbigniew Karczmarzyk[5], Emilia Fornal[6], Waldemar Wysocki[5], Paulina Bartnik[5]

[1] Independent Radiopharmacy Unit, Faculty of Pharmacy, Medical University of Lublin, PL-20093 Lublin , Poland

[2] Independent Medical Biology Unit, Faculty of Pharmacy, Medical University of Lublin, PL-20093 Lublin, Poland; agnieszkakorga@umlub.pl

[3] Institute of General and Ecological Chemistry, Faculty of Chemistry, Lodz University of Technology, Zeromskiego 116, 90-924 Lodz, Poland; 211150@edu.p.lodz.pl

[4] Chair and Department of Toxicology, Faculty of Pharmacy, Medical University of Lublin, PL-20093 Lublin, Poland; magda.iwan@umlub.pl

[5] Faculty of Science, Siedlce University of Natural Sciences and Humanities, 3 Maja 54, 08-110 Siedlce, Poland

[6] Chair and Department of Pathophysiology, Faculty of Medicine, Medical University of Lublin, PL-20090 Lublin

* Correspondence: monika.pitucha@umlub.pl; Tel.: 48-81-448-72-40 (MP)

X-ray structure determination

CIF for T2

```
data_shelx

_audit_creation_method           'SHELXL-2014/7'
_shelx_SHELXL_version_number     '2014/7'
_chemical_name_systematic         ?
_chemical_name_common             ?
_chemical_melting_point           ?
_chemical_formula_moiety          'C9 H10 Br N3 S'
_chemical_formula_sum             'C9 H10 Br N3 S'
_chemical_formula_weight          272.17

loop_
 _atom_type_symbol
 _atom_type_description
 _atom_type_scat_dispersion_real
 _atom_type_scat_dispersion_imag
 _atom_type_scat_source
 'C'  'C'   0.0033   0.0016
 'International Tables Vol C Tables 4.2.6.8 and 6.1.1.4'
 'H'  'H'   0.0000   0.0000
```

'International Tables Vol C Tables 4.2.6.8 and 6.1.1.4'
'N' 'N' 0.0061 0.0033
'International Tables Vol C Tables 4.2.6.8 and 6.1.1.4'
'S' 'S' 0.1246 0.1234
'International Tables Vol C Tables 4.2.6.8 and 6.1.1.4'
'Br' 'Br' -0.2901 2.4595
'International Tables Vol C Tables 4.2.6.8 and 6.1.1.4'

_space_group_crystal_system orthorhombic
_space_group_IT_number 61
_space_group_name_H-M_alt 'P b c a'
_space_group_name_Hall '-P 2ac 2ab'

_shelx_space_group_comment
;
The symmetry employed for this shelxl refinement is uniquely defined
by the following loop, which should always be used as a source of
symmetry information in preference to the above space-group names.
They are only intended as comments.
;

loop_
_space_group_symop_operation_xyz
'x, y, z'
'-x+1/2, -y, z+1/2'
'-x, y+1/2, -z+1/2'
'x+1/2, -y+1/2, -z'
'-x, -y, -z'
'x-1/2, y, -z-1/2'
'x, -y-1/2, z-1/2'
'-x-1/2, y-1/2, z'

_cell_length_a 13.5800(15)
_cell_length_b 8.5494(8)
_cell_length_c 19.0926(18)
_cell_angle_alpha 90
_cell_angle_beta 90
_cell_angle_gamma 90
_cell_volume 2216.7(4)
_cell_formula_units_Z 8
_cell_measurement_temperature 296(2)
_cell_measurement_reflns_used 2646
_cell_measurement_theta_min 2.6100
_cell_measurement_theta_max 26.1330

_exptl_crystal_description plate
_exptl_crystal_colour colourless
_exptl_crystal_density_meas ?
_exptl_crystal_density_method ?
_exptl_crystal_density_diffrn 1.631
_exptl_crystal_F_000 1088
_exptl_transmission_factor_min ?
_exptl_transmission_factor_max ?
_exptl_crystal_size_max 0.5
_exptl_crystal_size_mid 0.5
_exptl_crystal_size_min 0.1
_exptl_absorpt_coefficient_mu 3.862

_shelx_estimated_absorpt_T_min   ?
_shelx_estimated_absorpt_T_max   ?
_exptl_absorpt_correction_T_min   0.00545
_exptl_absorpt_correction_T_max   1.00000
_exptl_absorpt_correction_type   multi-scan
_exptl_absorpt_process_details
;
CrysAlisPro 1.171.39.46 (Rigaku Oxford Diffraction, 2018)
Empirical absorption correction using spherical harmonics,
 implemented in SCALE3 ABSPACK scaling algorithm.
;
_exptl_absorpt_special_details   ?
_diffrn_ambient_temperature   296(2)
_diffrn_radiation_wavelength   0.71073
_diffrn_radiation_type   MoK\a
_diffrn_source   ?
_diffrn_measurement_device_type   'KM4 CCD four-circle diffractometer'
_diffrn_measurement_method   ?
_diffrn_detector_area_resol_mean ?
_diffrn_reflns_number   12588
_diffrn_reflns_av_unetI/netI   0.0550
_diffrn_reflns_av_R_equivalents   0.0649
_diffrn_reflns_limit_h_min   -17
_diffrn_reflns_limit_h_max   17
_diffrn_reflns_limit_k_min   -10
_diffrn_reflns_limit_k_max   10
_diffrn_reflns_limit_l_min   -19
_diffrn_reflns_limit_l_max   23
_diffrn_reflns_theta_min   2.133
_diffrn_reflns_theta_max   28.098
_diffrn_reflns_theta_full   25.242
_diffrn_measured_fraction_theta_max   0.900
_diffrn_measured_fraction_theta_full 1.000
_diffrn_reflns_Laue_measured_fraction_max   0.900
_diffrn_reflns_Laue_measured_fraction_full   1.000
_diffrn_reflns_point_group_measured_fraction_max   0.900
_diffrn_reflns_point_group_measured_fraction_full 1.000
_reflns_number_total   2431
_reflns_number_gt   1713
_reflns_threshold_expression   'I > 2\s(I)'
_reflns_Friedel_coverage   0.000
_reflns_Friedel_fraction_max   .
_reflns_Friedel_fraction_full   .

_reflns_special_details
;
 Reflections were merged by SHELXL according to the crystal
 class for the calculation of statistics and refinement.

 _reflns_Friedel_fraction is defined as the number of unique
 Friedel pairs measured divided by the number that would be
 possible theoretically, ignoring centric projections and
 systematic absences.
;

_computing_data_collection
;

```
CrysAlisPro, Agilent Technologies,
Version 1.171.37.35h (release 09-02-2015 CrysAlis171 .NET)
(compiled Feb  9 2015,16:26:32)
;
_computing_cell_refinement
;
CrysAlisPro, Agilent Technologies,
Version 1.171.37.35h (release 09-02-2015 CrysAlis171 .NET)
(compiled Feb  9 2015,16:26:32)
;
_computing_data_reduction
;
CrysAlisPro, Agilent Technologies,
Version 1.171.37.35h (release 09-02-2015 CrysAlis171 .NET)
(compiled Feb  9 2015,16:26:32)
;
_computing_structure_solution     'SHELXS-2013/1 (Sheldrick, 2013)'
_computing_structure_refinement 'SHELXL-2014/7 (Sheldrick, 2014)'
_computing_molecular_graphics     ?
_computing_publication_material   ?
_refine_special_details           ?
_refine_ls_structure_factor_coef  Fsqd
_refine_ls_matrix_type            full
_refine_ls_weighting_scheme       calc
_refine_ls_weighting_details
'w=1/[\s^2^(Fo^2^)+(0.0805P)^2^] where P=(Fo^2^+2Fc^2^)/3'
_atom_sites_solution_primary      refxyz
_atom_sites_solution_secondary    ?
_atom_sites_solution_hydrogens    mixed
_refine_ls_hydrogen_treatment     mixed
_refine_ls_extinction_method      'SHELXL-2014/7 (Sheldrick 2014'
_refine_ls_extinction_coef        0.0020(6)
_refine_ls_extinction_expression
 'Fc^*^=kFc[1+0.001xFc^2^\l^3^/sin(2\q)]^-1/4^'
_refine_ls_number_reflns          2431
_refine_ls_number_parameters      135
_refine_ls_number_restraints      0
_refine_ls_R_factor_all           0.0759
_refine_ls_R_factor_gt            0.0489
_refine_ls_wR_factor_ref          0.1477
_refine_ls_wR_factor_gt           0.1286
_refine_ls_goodness_of_fit_ref    1.009
_refine_ls_restrained_S_all       1.009
_refine_ls_shift/su_max           0.001
_refine_ls_shift/su_mean          0.000

loop_
 _atom_site_label
 _atom_site_type_symbol
 _atom_site_fract_x
 _atom_site_fract_y
 _atom_site_fract_z
 _atom_site_U_iso_or_equiv
 _atom_site_adp_type
 _atom_site_occupancy
 _atom_site_site_symmetry_order
 _atom_site_calc_flag
```

_atom_site_refinement_flags_posn
_atom_site_refinement_flags_adp
_atom_site_refinement_flags_occupancy
_atom_site_disorder_assembly
_atom_site_disorder_group
C2 C 0.6286(2) 0.1361(4) 0.5668(2) 0.0355(8) Uani 1 1 d . . . . .
C6 C 0.5734(3) 0.3942(4) 0.4322(2) 0.0445(9) Uani 1 1 d . . . . .
H6 H 0.5320 0.3270 0.4077 0.067 Uiso 1 1 calc R U . . .
C7 C 0.7393(4) 0.1713(5) 0.6660(2) 0.0650(13) Uani 1 1 d . . . . .
H7A H 0.6908 0.1313 0.6979 0.097 Uiso 1 1 calc R U . . .
H7B H 0.7727 0.2582 0.6872 0.097 Uiso 1 1 calc R U . . .
H7C H 0.7861 0.0906 0.6553 0.097 Uiso 1 1 calc R U . . .
C21 C 0.5917(3) 0.5502(4) 0.4042(2) 0.0380(9) Uani 1 1 d . . . . .
C22 C 0.6431(2) 0.6627(4) 0.4409(2) 0.0420(9) Uani 1 1 d . . . . .
H22 H 0.6670 0.6396 0.4853 0.063 Uiso 1 1 calc R U . . .
C23 C 0.6597(3) 0.8085(4) 0.4128(2) 0.0433(9) Uani 1 1 d . . . . .
H23 H 0.6946 0.8835 0.4379 0.065 Uiso 1 1 calc R U . . .
C24 C 0.6240(2) 0.8416(4) 0.3471(2) 0.0404(9) Uani 1 1 d . . . . .
C25 C 0.5725(3) 0.7340(5) 0.3093(2) 0.0535(11) Uani 1 1 d . . . . .
H25 H 0.5490 0.7583 0.2649 0.080 Uiso 1 1 calc R U . . .
C26 C 0.5559(3) 0.5885(5) 0.3382(2) 0.0514(10) Uani 1 1 d . . . . .
H26 H 0.5202 0.5147 0.3130 0.077 Uiso 1 1 calc R U . . .
N1 N 0.6915(3) 0.2222(4) 0.60225(18) 0.0472(8) Uani 1 1 d . . . . .
H1 H 0.703(4) 0.304(6) 0.586(3) 0.071 Uiso 1 1 d . U . . .
N4 N 0.5902(2) 0.1965(3) 0.50833(18) 0.0435(8) Uani 1 1 d . . . . .
H4 H 0.546(3) 0.150(5) 0.490(2) 0.065 Uiso 1 1 d . U . . .
N5 N 0.6121(2) 0.3472(3) 0.48877(17) 0.0386(7) Uani 1 1 d . . . . .
S3 S 0.59704(7) -0.04697(10) 0.59064(6) 0.0465(3) Uani 1 1 d . . . . .
Br27 Br 0.64839(3) 1.04319(5) 0.30901(2) 0.0548(2) Uani 1 1 d . . . . .

loop_
_atom_site_aniso_label
_atom_site_aniso_U_11
_atom_site_aniso_U_22
_atom_site_aniso_U_33
_atom_site_aniso_U_23
_atom_site_aniso_U_13
_atom_site_aniso_U_12
C2 0.0300(16) 0.0337(19) 0.043(2) -0.0003(15) -0.0009(16) 0.0040(14)
C6 0.0403(19) 0.036(2) 0.058(3) 0.0004(18) -0.0064(18) -0.0028(15)
C7 0.084(3) 0.058(3) 0.053(3) 0.009(2) -0.021(3) -0.021(2)
C21 0.0338(18) 0.036(2) 0.044(2) 0.0017(15) -0.0036(17) 0.0000(14)
C22 0.044(2) 0.041(2) 0.041(2) 0.0001(17) -0.0047(17) 0.0002(15)
C23 0.045(2) 0.0322(19) 0.052(3) -0.0025(17) -0.0034(17) -0.0040(15)
C24 0.0344(17) 0.0375(19) 0.049(2) 0.0065(17) 0.0033(18) -0.0012(14)
C25 0.061(3) 0.051(2) 0.048(3) 0.0087(19) -0.015(2) -0.005(2)
C26 0.057(2) 0.049(2) 0.048(2) 0.001(2) -0.019(2) -0.0117(18)
N1 0.055(2) 0.0360(17) 0.050(2) 0.0054(15) -0.0085(17) -0.0097(15)
N4 0.0383(16) 0.0337(16) 0.059(2) 0.0055(15) -0.0096(15) -0.0071(12)
N5 0.0383(15) 0.0313(15) 0.0464(19) 0.0046(13) -0.0020(15) -0.0025(12)
S3 0.0461(6) 0.0321(5) 0.0613(7) 0.0075(4) -0.0076(5) -0.0041(4)
Br27 0.0630(4) 0.0426(3) 0.0589(4) 0.01154(18) 0.0052(2) -0.00537(17)

_geom_special_details
;
All esds (except the esd in the dihedral angle between two l.s. planes)
are estimated using the full covariance matrix. The cell esds are taken

into account individually in the estimation of esds in distances, angles
and torsion angles; correlations between esds in cell parameters are only
used when they are defined by crystal symmetry. An approximate (isotropic)
treatment of cell esds is used for estimating esds involving l.s. planes.
;

```
loop_
 _geom_bond_atom_site_label_1
 _geom_bond_atom_site_label_2
 _geom_bond_distance
 _geom_bond_site_symmetry_2
 _geom_bond_publ_flag
C2 N1 1.316(5) . ?
C2 N4 1.335(5) . ?
C2 S3 1.686(4) . ?
C6 N5 1.266(5) . ?
C6 C21 1.458(5) . ?
C6 H6 0.9300 . ?
C7 N1 1.446(5) . ?
C7 H7A 0.9600 . ?
C7 H7B 0.9600 . ?
C7 H7C 0.9600 . ?
C21 C22 1.379(5) . ?
C21 C26 1.391(5) . ?
C22 C23 1.375(6) . ?
C22 H22 0.9300 . ?
C23 C24 1.374(5) . ?
C23 H23 0.9300 . ?
C24 C25 1.362(5) . ?
C24 Br27 1.900(3) . ?
C25 C26 1.379(6) . ?
C25 H25 0.9300 . ?
C26 H26 0.9300 . ?
N1 H1 0.78(5) . ?
N4 N5 1.373(4) . ?
N4 H4 0.80(4) . ?

loop_
 _geom_angle_atom_site_label_1
 _geom_angle_atom_site_label_2
 _geom_angle_atom_site_label_3
 _geom_angle
 _geom_angle_site_symmetry_1
 _geom_angle_site_symmetry_3
 _geom_angle_publ_flag
N1 C2 N4 117.9(3) . . ?
N1 C2 S3 123.0(3) . . ?
N4 C2 S3 119.1(3) . . ?
N5 C6 C21 122.2(3) . . ?
N5 C6 H6 118.9 . . ?
C21 C6 H6 118.9 . . ?
N1 C7 H7A 109.5 . . ?
N1 C7 H7B 109.5 . . ?
H7A C7 H7B 109.5 . . ?
N1 C7 H7C 109.5 . . ?
H7A C7 H7C 109.5 . . ?
H7B C7 H7C 109.5 . . ?
```

C22 C21 C26 118.2(3) . . ?
C22 C21 C6 122.6(4) . . ?
C26 C21 C6 119.3(3) . . ?
C23 C22 C21 121.2(4) . . ?
C23 C22 H22 119.4 . . ?
C21 C22 H22 119.4 . . ?
C24 C23 C22 118.9(3) . . ?
C24 C23 H23 120.5 . . ?
C22 C23 H23 120.5 . . ?
C25 C24 C23 121.8(3) . . ?
C25 C24 Br27 119.9(3) . . ?
C23 C24 Br27 118.3(3) . . ?
C24 C25 C26 118.7(4) . . ?
C24 C25 H25 120.6 . . ?
C26 C25 H25 120.6 . . ?
C25 C26 C21 121.2(4) . . ?
C25 C26 H26 119.4 . . ?
C21 C26 H26 119.4 . . ?
C2 N1 C7 123.8(3) . . ?
C2 N1 H1 116(4) . . ?
C7 N1 H1 120(4) . . ?
C2 N4 N5 120.4(3) . . ?
C2 N4 H4 118(3) . . ?
N5 N4 H4 120(3) . . ?
C6 N5 N4 116.1(3) . . ?

loop_
 _geom_torsion_atom_site_label_1
 _geom_torsion_atom_site_label_2
 _geom_torsion_atom_site_label_3
 _geom_torsion_atom_site_label_4
 _geom_torsion
 _geom_torsion_site_symmetry_1
 _geom_torsion_site_symmetry_2
 _geom_torsion_site_symmetry_3
 _geom_torsion_site_symmetry_4
 _geom_torsion_publ_flag
N5 C6 C21 C22 -7.3(6) . . . . ?
N5 C6 C21 C26 172.9(4) . . . . ?
C26 C21 C22 C23 -0.6(6) . . . . ?
C6 C21 C22 C23 179.6(3) . . . . ?
C21 C22 C23 C24 0.1(5) . . . . ?
C22 C23 C24 C25 0.2(6) . . . . ?
C22 C23 C24 Br27 -179.6(3) . . . . ?
C23 C24 C25 C26 0.2(6) . . . . ?
Br27 C24 C25 C26 179.9(3) . . . . ?
C24 C25 C26 C21 -0.7(7) . . . . ?
C22 C21 C26 C25 1.0(6) . . . . ?
C6 C21 C26 C25 -179.2(4) . . . . ?
N4 C2 N1 C7 -179.5(4) . . . . ?
S3 C2 N1 C7 -1.1(6) . . . . ?
N1 C2 N4 N5 -3.7(5) . . . . ?
S3 C2 N4 N5 177.9(3) . . . . ?
C21 C6 N5 N4 -178.7(3) . . . . ?
C2 N4 N5 C6 179.2(3) . . . . ?

_refine_diff_density_max   0.675

_refine_diff_density_min   -0.457
_refine_diff_density_rms   0.107

_shelx_res_file
;

```
    shelx.res created by SHELXL-2014/7


TITL T2_20191007_03 in Pbca
CELL  0.71073  13.5800   8.5494  19.0926   90.000   90.000   90.000
ZERR     8.00   0.0015   0.0008   0.0018    0.000    0.000    0.000
LATT   1
SYMM  1/2 - X, - Y, 1/2 + Z
SYMM  - X, 1/2 + Y, 1/2 - Z
SYMM  1/2 + X, 1/2 - Y, - Z
SFAC  C    H    N    S    BR
UNIT  72 80 24 8 8
MERG   2
FMAP   2
PLAN   -30
ACTA
BOND   $H
CONF
L.S.  30
WGHT    0.080500
EXTI    0.001960
FVAR       2.47847
MOLE    1
C2    1    0.628599    0.136142    0.566764    11.00000    0.03004    0.03371 =
        0.04288   -0.00034   -0.00089    0.00396
C6    1    0.573400    0.394177    0.432235    11.00000    0.04033    0.03561 =
        0.05770    0.00042   -0.00637   -0.00281
AFIX  43
H6    2    0.531971    0.327005    0.407748    11.00000   -1.50000
AFIX   0
C7    1    0.739275    0.171311    0.665992    11.00000    0.08381    0.05812 =
        0.05304    0.00926   -0.02125   -0.02098
AFIX 137
H7A   2    0.690776    0.131273    0.697879    11.00000   -1.50000
H7B   2    0.772686    0.258247    0.687160    11.00000   -1.50000
H7C   2    0.786069    0.090630    0.655257    11.00000   -1.50000
AFIX   0
C21   1    0.591673    0.550154    0.404239    11.00000    0.03380    0.03596 =
        0.04431    0.00171   -0.00362    0.00003
C22   1    0.643125    0.662699    0.440858    11.00000    0.04368    0.04113 =
        0.04129    0.00013   -0.00471    0.00020
AFIX  43
H22   2    0.667025    0.639558    0.485313    11.00000   -1.50000
AFIX   0
C23   1    0.659718    0.808515    0.412813    11.00000    0.04542    0.03219 =
        0.05233   -0.00254   -0.00341   -0.00401
AFIX  43
H23   2    0.694576    0.883537    0.437901    11.00000   -1.50000
AFIX   0
C24   1    0.623969    0.841576    0.347133    11.00000    0.03444    0.03752 =
        0.04933    0.00652    0.00332   -0.00125
```

```
C25   1    0.572496    0.734044    0.309309    11.00000    0.06066    0.05145 =
        0.04846    0.00874   -0.01545   -0.00540
AFIX  43
H25   2    0.548972    0.758294    0.264871    11.00000   -1.50000
AFIX   0
C26   1    0.555921    0.588490    0.338169    11.00000    0.05670    0.04922 =
        0.04818    0.00135   -0.01900   -0.01165
AFIX  43
H26   2    0.520195    0.514657    0.312992    11.00000   -1.50000
AFIX   0
N1    3    0.691525    0.222195    0.602253    11.00000    0.05524    0.03596 =
        0.05031    0.00542   -0.00846   -0.00966
H1    2    0.703428    0.304255    0.586235    11.00000   -1.50000
N4    3    0.590234    0.196545    0.508332    11.00000    0.03826    0.03370 =
        0.05856    0.00552   -0.00962   -0.00714
H4    2    0.545851    0.150487    0.489752    11.00000   -1.50000
N5    3    0.612054    0.347160    0.488766    11.00000    0.03826    0.03127 =
        0.04640    0.00456   -0.00202   -0.00253
S3    4    0.597036   -0.046970    0.590640    11.00000    0.04613    0.03205 =
        0.06130    0.00747   -0.00762   -0.00415
BR27  5    0.648392    1.043191    0.309010    11.00000    0.06302    0.04261 =
        0.05891    0.01154    0.00523   -0.00537
HKLF    4

REM  T2_20191007_03 in Pbca
REM R1 =  0.0489 for    1713 Fo > 4sig(Fo)  and  0.0759 for all    2431 data
REM    135 parameters refined using      0 restraints

END

WGHT      0.0806      0.0000

REM Highest difference peak  0.675,  deepest hole -0.457,  1-sigma level  0.107
Q1    1   0.7393  1.0172  0.3084  11.00000  0.05    0.67
Q2    1   0.6866  1.0520  0.3563  11.00000  0.05    0.51
Q3    1   0.5556  1.0251  0.3087  11.00000  0.05    0.51
Q4    1   0.6481  0.8915  0.3087  11.00000  0.05    0.47
Q5    1   0.7321  0.9108  0.3058  11.00000  0.05    0.41
Q6    1   0.7632  1.1983  0.3082  11.00000  0.05    0.39
Q7    1   0.6503  1.1956  0.3104  11.00000  0.05    0.38
Q8    1   0.6022  1.0703  0.3587  11.00000  0.05    0.38
Q9    1   0.5229 -0.0122  0.5885  11.00000  0.05    0.38
Q10   1   0.6944  1.0479  0.2581  11.00000  0.05    0.37
Q11   1   0.6588  0.7615  0.3090  11.00000  0.05    0.36
Q12   1   0.6988 -0.0535  0.5932  11.00000  0.05    0.35
Q13   1   0.6437 -0.0471  0.6403  11.00000  0.05    0.35
Q14   1   0.5615  0.9315  0.3067  11.00000  0.05    0.35
Q15   1   0.6023  1.0274  0.2580  11.00000  0.05    0.31
Q16   1   0.4765  0.5989  0.4016  11.00000  0.05    0.30
Q17   1   0.7105  0.8007  0.3010  11.00000  0.05    0.30
Q18   1   0.4563  0.5559  0.3193  11.00000  0.05    0.29
Q19   1   0.5565  0.7469  0.1882  11.00000  0.05    0.28
Q20   1   0.7359  0.0590  0.6829  11.00000  0.05    0.28
Q21   1   0.5843  1.2311  0.3045  11.00000  0.05    0.27
Q22   1   0.6666  1.3203  0.3094  11.00000  0.05    0.27
Q23   1   0.7618  0.3288  0.5913  11.00000  0.05    0.27
Q24   1   0.6815  1.2592  0.3004  11.00000  0.05    0.26
```

```
Q25   1   0.4334  0.7941  0.3078  11.00000  0.05    0.25
Q26   1   0.4826  0.0672  0.5842  11.00000  0.05    0.25
Q27   1   0.7406  0.2973  0.5732  11.00000  0.05    0.25
Q28   1   0.4815  0.4325  0.3137  11.00000  0.05    0.25
Q29   1   0.6620  0.9586  0.2489  11.00000  0.05    0.24
Q30   1   0.5563 -0.0359  0.6415  11.00000  0.05    0.24
;
```

CIF for T3

```
data_shelx

_audit_creation_method            'SHELXL-2014/7'
_shelx_SHELXL_version_number      '2014/7'
_chemical_name_systematic         ?
_chemical_name_common             ?
_chemical_melting_point           ?
_chemical_formula_moiety          'C11 H15 N3 O2 S'
_chemical_formula_sum             'C11 H15 N3 O2 S'
_chemical_formula_weight          253.32

loop_
 _atom_type_symbol
 _atom_type_description
 _atom_type_scat_dispersion_real
 _atom_type_scat_dispersion_imag
 _atom_type_scat_source
 'C'  'C'   0.0033   0.0016
 'International Tables Vol C Tables 4.2.6.8 and 6.1.1.4'
 'H'  'H'   0.0000   0.0000
 'International Tables Vol C Tables 4.2.6.8 and 6.1.1.4'
 'N'  'N'   0.0061   0.0033
 'International Tables Vol C Tables 4.2.6.8 and 6.1.1.4'
 'O'  'O'   0.0106   0.0060
 'International Tables Vol C Tables 4.2.6.8 and 6.1.1.4'
 'S'  'S'   0.1246   0.1234
 'International Tables Vol C Tables 4.2.6.8 and 6.1.1.4'

_space_group_crystal_system       monoclinic
_space_group_IT_number            14
_space_group_name_H-M_alt         'P 21/c'
_space_group_name_Hall            '-P 2ybc'

_shelx_space_group_comment
;
The symmetry employed for this shelxl refinement is uniquely defined
by the following loop, which should always be used as a source of
symmetry information in preference to the above space-group names.
They are only intended as comments.
;

loop_
 _space_group_symop_operation_xyz
 'x, y, z'
 '-x, y+1/2, -z+1/2'
 '-x, -y, -z'
```

'x, -y-1/2, z-1/2'

_cell_length_a 8.5409(7)
_cell_length_b 8.1329(5)
_cell_length_c 18.8922(12)
_cell_angle_alpha 90
_cell_angle_beta 102.099(7)
_cell_angle_gamma 90
_cell_volume 1283.15(16)
_cell_formula_units_Z 4
_cell_measurement_temperature 296(2)
_cell_measurement_reflns_used 4130
_cell_measurement_theta_min 2.2510
_cell_measurement_theta_max 27.8070

_exptl_crystal_description block
_exptl_crystal_colour colourless
_exptl_crystal_density_meas ?
_exptl_crystal_density_method ?
_exptl_crystal_density_diffrn 1.311
_exptl_crystal_F_000 536
_exptl_transmission_factor_min ?
_exptl_transmission_factor_max ?
_exptl_crystal_size_max 0.5
_exptl_crystal_size_mid 0.5
_exptl_crystal_size_min 0.3
_exptl_absorpt_coefficient_mu 0.247
_shelx_estimated_absorpt_T_min ?
_shelx_estimated_absorpt_T_max ?
_exptl_absorpt_correction_T_min 0.75249
_exptl_absorpt_correction_T_max 1.00000
_exptl_absorpt_correction_type multi-scan
_exptl_absorpt_process_details
;
CrysAlisPro, Agilent Technologies,
Version 1.171.37.35h (release 09-02-2015 CrysAlis171 .NET)
(compiled Feb 9 2015,16:26:32)
Empirical absorption correction using spherical harmonics,
implemented in SCALE3 ABSPACK scaling algorithm.
;
_exptl_absorpt_special_details ?
_diffrn_ambient_temperature 296(2)
_diffrn_radiation_wavelength 0.71073
_diffrn_radiation_type MoK\a
_diffrn_source ?
_diffrn_measurement_device_type 'KM4 CCD four-circle diffractometer'
_diffrn_measurement_method ?
_diffrn_detector_area_resol_mean ?
_diffrn_reflns_number 8182
_diffrn_reflns_av_unetI/netI 0.0156
_diffrn_reflns_av_R_equivalents 0.0177
_diffrn_reflns_limit_h_min -10
_diffrn_reflns_limit_h_max 10
_diffrn_reflns_limit_k_min -9
_diffrn_reflns_limit_k_max 6
_diffrn_reflns_limit_l_min -22
_diffrn_reflns_limit_l_max 22

_diffrn_reflns_theta_min 2.205
_diffrn_reflns_theta_max 27.778
_diffrn_reflns_theta_full 25.242
_diffrn_measured_fraction_theta_max 0.879
_diffrn_measured_fraction_theta_full 1.000
_diffrn_reflns_Laue_measured_fraction_max 0.879
_diffrn_reflns_Laue_measured_fraction_full 1.000
_diffrn_reflns_point_group_measured_fraction_max 0.879
_diffrn_reflns_point_group_measured_fraction_full 1.000
_reflns_number_total 2666
_reflns_number_gt 2366
_reflns_threshold_expression 'I > 2\s(I)'
_reflns_Friedel_coverage 0.000
_reflns_Friedel_fraction_max .
_reflns_Friedel_fraction_full .

_reflns_special_details
;
Reflections were merged by SHELXL according to the crystal
class for the calculation of statistics and refinement.

_reflns_Friedel_fraction is defined as the number of unique
Friedel pairs measured divided by the number that would be
possible theoretically, ignoring centric projections and
systematic absences.
;

_computing_data_collection
;
CrysAlisPro, Agilent Technologies,
Version 1.171.37.35h (release 09-02-2015 CrysAlis171 .NET)
(compiled Feb 9 2015,16:26:32)
;
_computing_cell_refinement
;
CrysAlisPro, Agilent Technologies,
Version 1.171.37.35h (release 09-02-2015 CrysAlis171 .NET)
(compiled Feb 9 2015,16:26:32)
;
_computing_data_reduction
;
CrysAlisPro, Agilent Technologies,
Version 1.171.37.35h (release 09-02-2015 CrysAlis171 .NET)
(compiled Feb 9 2015,16:26:32)
;
_computing_structure_solution 'SHELXS-2013/1 (Sheldrick, 2013)'
_computing_structure_refinement 'SHELXL-2014/7 (Sheldrick, 2014)'
_computing_molecular_graphics ?
_computing_publication_material ?
_refine_special_details ?
_refine_ls_structure_factor_coef Fsqd
_refine_ls_matrix_type full
_refine_ls_weighting_scheme calc
_refine_ls_weighting_details
'w=1/[\s^2^(Fo^2^)+(0.0426P)^2^+0.3621P] where P=(Fo^2^+2Fc^2^)/3'
_atom_sites_solution_primary refxyz
_atom_sites_solution_secondary ?

_atom_sites_solution_hydrogens mixed
_refine_ls_hydrogen_treatment mixed
_refine_ls_extinction_method 'SHELXL-2014/7 (Sheldrick 2014'
_refine_ls_extinction_coef 0.0142(17)
_refine_ls_extinction_expression
'Fc^*^=kFc[1+0.001xFc^2^\l^3^/sin(2\q)]^-1/4^'
_refine_ls_number_reflns 2666
_refine_ls_number_parameters 164
_refine_ls_number_restraints 0
_refine_ls_R_factor_all 0.0362
_refine_ls_R_factor_gt 0.0313
_refine_ls_wR_factor_ref 0.0881
_refine_ls_wR_factor_gt 0.0846
_refine_ls_goodness_of_fit_ref 1.045
_refine_ls_restrained_S_all 1.045
_refine_ls_shift/su_max 0.000
_refine_ls_shift/su_mean 0.000

loop_
_atom_site_label
_atom_site_type_symbol
_atom_site_fract_x
_atom_site_fract_y
_atom_site_fract_z
_atom_site_U_iso_or_equiv
_atom_site_adp_type
_atom_site_occupancy
_atom_site_site_symmetry_order
_atom_site_calc_flag
_atom_site_refinement_flags_posn
_atom_site_refinement_flags_adp
_atom_site_refinement_flags_occupancy
_atom_site_disorder_assembly
_atom_site_disorder_group
C2 C 0.99910(15) -0.12039(15) 0.11931(7) 0.0311(3) Uani 1 1 d . . . . .
C6 C 0.64184(15) 0.10073(18) 0.07411(7) 0.0376(3) Uani 1 1 d . . . . .
H6 H 0.6523 0.1226 0.0270 0.056 Uiso 1 1 calc R U . . .
C7 C 1.1177(2) -0.2616(2) 0.23209(8) 0.0546(4) Uani 1 1 d . . . . .
H7A H 1.1537 -0.3494 0.2056 0.082 Uiso 1 1 calc R U . . .
H7B H 1.0787 -0.3061 0.2722 0.082 Uiso 1 1 calc R U . . .
H7C H 1.2051 -0.1881 0.2498 0.082 Uiso 1 1 calc R U . . .
C21 C 0.50276(16) 0.16718(16) 0.09795(7) 0.0352(3) Uani 1 1 d . . . . .
C22 C 0.47854(15) 0.14654(16) 0.16873(7) 0.0332(3) Uani 1 1 d . . . . .
H22 H 0.5534 0.0895 0.2028 0.050 Uiso 1 1 calc R U . . .
C23 C 0.34378(15) 0.21090(16) 0.18766(7) 0.0336(3) Uani 1 1 d . . . . .
C24 C 0.22811(16) 0.29395(17) 0.13577(7) 0.0361(3) Uani 1 1 d . . . . .
C25 C 0.25324(18) 0.3157(2) 0.06673(8) 0.0461(4) Uani 1 1 d . . . . .
H25 H 0.1784 0.3723 0.0325 0.069 Uiso 1 1 calc R U . . .
C26 C 0.39027(18) 0.2532(2) 0.04822(8) 0.0459(4) Uani 1 1 d . . . . .
H26 H 0.4069 0.2693 0.0016 0.069 Uiso 1 1 calc R U . . .
C28 C 0.4277(2) 0.1368(3) 0.31217(8) 0.0589(5) Uani 1 1 d . . . . .
H28A H 0.4518 0.0253 0.3016 0.088 Uiso 1 1 calc R U . . .
H28B H 0.3893 0.1400 0.3564 0.088 Uiso 1 1 calc R U . . .
H28C H 0.5228 0.2025 0.3173 0.088 Uiso 1 1 calc R U . . .
C29 C -0.03031(18) 0.4177(2) 0.10612(9) 0.0511(4) Uani 1 1 d . . . . .
H29A H 0.0074 0.5164 0.0873 0.077 Uiso 1 1 calc R U . . .
H29B H -0.1190 0.4435 0.1281 0.077 Uiso 1 1 calc R U . . .

H29C H -0.0642 0.3408 0.0674 0.077 Uiso 1 1 calc R U . . .
N1 N 0.99009(15) -0.17262(15) 0.18497(6) 0.0397(3) Uani 1 1 d . . . . .
H1 H 0.910(2) -0.151(2) 0.1999(11) 0.060 Uiso 1 1 d . U . . .
N4 N 0.87451(13) -0.03239(15) 0.08246(6) 0.0384(3) Uani 1 1 d . . . . .
H4 H 0.875(2) 0.003(2) 0.0399(10) 0.058 Uiso 1 1 d . U . . .
N5 N 0.74994(12) 0.01429(14) 0.11432(6) 0.0343(3) Uani 1 1 d . . . . .
O27 O 0.30829(12) 0.19967(15) 0.25478(5) 0.0484(3) Uani 1 1 d . . . . .
O28 O 0.09617(12) 0.34676(13) 0.15915(6) 0.0470(3) Uani 1 1 d . . . . .
S3 S 1.15525(4) -0.16089(5) 0.07998(2) 0.04145(14) Uani 1 1 d . . . . .

loop_
 _atom_site_aniso_label
 _atom_site_aniso_U_11
 _atom_site_aniso_U_22
 _atom_site_aniso_U_33
 _atom_site_aniso_U_23
 _atom_site_aniso_U_13
 _atom_site_aniso_U_12
C2 0.0310(6) 0.0328(6) 0.0308(6) -0.0020(5) 0.0095(5) -0.0009(5)
C6 0.0331(7) 0.0505(8) 0.0315(7) -0.0005(6) 0.0118(5) 0.0045(6)
C7 0.0624(10) 0.0661(10) 0.0351(8) 0.0077(7) 0.0096(7) 0.0237(8)
C21 0.0305(6) 0.0428(7) 0.0335(7) -0.0023(5) 0.0096(5) 0.0028(5)
C22 0.0282(6) 0.0394(7) 0.0320(7) -0.0015(5) 0.0061(5) 0.0022(5)
C23 0.0320(6) 0.0388(7) 0.0314(6) -0.0038(5) 0.0099(5) 0.0003(5)
C24 0.0309(6) 0.0381(7) 0.0408(7) -0.0033(6) 0.0109(6) 0.0054(5)
C25 0.0408(8) 0.0585(9) 0.0392(8) 0.0082(6) 0.0090(6) 0.0170(7)
C26 0.0433(8) 0.0626(10) 0.0343(7) 0.0063(6) 0.0134(6) 0.0122(7)
C28 0.0537(10) 0.0899(13) 0.0341(8) 0.0095(8) 0.0118(7) 0.0140(9)
C29 0.0365(8) 0.0547(9) 0.0623(10) 0.0063(8) 0.0109(7) 0.0139(7)
N1 0.0397(7) 0.0502(7) 0.0322(6) 0.0050(5) 0.0145(5) 0.0101(5)
N4 0.0339(6) 0.0546(7) 0.0302(6) 0.0059(5) 0.0146(5) 0.0108(5)
N5 0.0299(5) 0.0434(6) 0.0321(6) -0.0029(5) 0.0124(4) 0.0034(5)
O27 0.0401(6) 0.0754(7) 0.0326(5) 0.0034(5) 0.0142(4) 0.0150(5)
O28 0.0379(5) 0.0587(6) 0.0478(6) 0.0048(5) 0.0164(5) 0.0182(5)
S3 0.0340(2) 0.0526(2) 0.0424(2) 0.00832(15) 0.01854(15) 0.00904(14)

_geom_special_details
;
 All esds (except the esd in the dihedral angle between two l.s. planes)
 are estimated using the full covariance matrix. The cell esds are taken
 into account individually in the estimation of esds in distances, angles
 and torsion angles; correlations between esds in cell parameters are only
 used when they are defined by crystal symmetry. An approximate (isotropic)
 treatment of cell esds is used for estimating esds involving l.s. planes.
;

loop_
 _geom_bond_atom_site_label_1
 _geom_bond_atom_site_label_2
 _geom_bond_distance
 _geom_bond_site_symmetry_2
 _geom_bond_publ_flag
C2 N1 1.3285(17) . ?
C2 N4 1.3487(17) . ?
C2 S3 1.6896(13) . ?
C6 N5 1.2766(17) . ?
C6 C21 1.4595(18) . ?

C6 H6 0.9300 . ?
C7 N1 1.4479(18) . ?
C7 H7A 0.9600 . ?
C7 H7B 0.9600 . ?
C7 H7C 0.9600 . ?
C21 C26 1.3846(19) . ?
C21 C22 1.4053(18) . ?
C22 C23 1.3781(18) . ?
C22 H22 0.9300 . ?
C23 O27 1.3671(16) . ?
C23 C24 1.4098(19) . ?
C24 O28 1.3631(16) . ?
C24 C25 1.377(2) . ?
C25 C26 1.386(2) . ?
C25 H25 0.9300 . ?
C26 H26 0.9300 . ?
C28 O27 1.4192(18) . ?
C28 H28A 0.9600 . ?
C28 H28B 0.9600 . ?
C28 H28C 0.9600 . ?
C29 O28 1.4317(18) . ?
C29 H29A 0.9600 . ?
C29 H29B 0.9600 . ?
C29 H29C 0.9600 . ?
N1 H1 0.813(19) . ?
N4 N5 1.3809(14) . ?
N4 H4 0.854(19) . ?

loop_
 _geom_angle_atom_site_label_1
 _geom_angle_atom_site_label_2
 _geom_angle_atom_site_label_3
 _geom_angle
 _geom_angle_site_symmetry_1
 _geom_angle_site_symmetry_3
 _geom_angle_publ_flag
N1 C2 N4 117.25(11) . . ?
N1 C2 S3 123.63(10) . . ?
N4 C2 S3 119.11(9) . . ?
N5 C6 C21 123.66(12) . . ?
N5 C6 H6 118.2 . . ?
C21 C6 H6 118.2 . . ?
N1 C7 H7A 109.5 . . ?
N1 C7 H7B 109.5 . . ?
H7A C7 H7B 109.5 . . ?
N1 C7 H7C 109.5 . . ?
H7A C7 H7C 109.5 . . ?
H7B C7 H7C 109.5 . . ?
C26 C21 C22 119.10(12) . . ?
C26 C21 C6 118.14(12) . . ?
C22 C21 C6 122.75(12) . . ?
C23 C22 C21 119.94(12) . . ?
C23 C22 H22 120.0 . . ?
C21 C22 H22 120.0 . . ?
O27 C23 C22 125.10(12) . . ?
O27 C23 C24 114.69(11) . . ?
C22 C23 C24 120.21(12) . . ?

O28 C24 C25 124.75(12) . . ?
O28 C24 C23 115.60(12) . . ?
C25 C24 C23 119.65(12) . . ?
C24 C25 C26 119.94(13) . . ?
C24 C25 H25 120.0 . . ?
C26 C25 H25 120.0 . . ?
C21 C26 C25 121.11(13) . . ?
C21 C26 H26 119.4 . . ?
C25 C26 H26 119.4 . . ?
O27 C28 H28A 109.5 . . ?
O27 C28 H28B 109.5 . . ?
H28A C28 H28B 109.5 . . ?
O27 C28 H28C 109.5 . . ?
H28A C28 H28C 109.5 . . ?
H28B C28 H28C 109.5 . . ?
O28 C29 H29A 109.5 . . ?
O28 C29 H29B 109.5 . . ?
H29A C29 H29B 109.5 . . ?
O28 C29 H29C 109.5 . . ?
H29A C29 H29C 109.5 . . ?
H29B C29 H29C 109.5 . . ?
C2 N1 C7 123.39(12) . . ?
C2 N1 H1 118.2(14) . . ?
C7 N1 H1 118.3(14) . . ?
C2 N4 N5 121.16(11) . . ?
C2 N4 H4 120.0(12) . . ?
N5 N4 H4 118.6(12) . . ?
C6 N5 N4 114.79(11) . . ?
C23 O27 C28 117.97(11) . . ?
C24 O28 C29 117.00(12) . . ?

loop_
_geom_torsion_atom_site_label_1
_geom_torsion_atom_site_label_2
_geom_torsion_atom_site_label_3
_geom_torsion_atom_site_label_4
_geom_torsion
_geom_torsion_site_symmetry_1
_geom_torsion_site_symmetry_2
_geom_torsion_site_symmetry_3
_geom_torsion_site_symmetry_4
_geom_torsion_publ_flag
N5 C6 C21 C26 -177.27(14) . . . . ?
N5 C6 C21 C22 2.4(2) . . . . ?
C26 C21 C22 C23 0.3(2) . . . . ?
C6 C21 C22 C23 -179.32(13) . . . . ?
C21 C22 C23 O27 -179.46(13) . . . . ?
C21 C22 C23 C24 1.6(2) . . . . ?
O27 C23 C24 O28 -1.78(18) . . . . ?
C22 C23 C24 O28 177.31(12) . . . . ?
O27 C23 C24 C25 178.55(13) . . . . ?
C22 C23 C24 C25 -2.4(2) . . . . ?
O28 C24 C25 C26 -178.37(14) . . . . ?
C23 C24 C25 C26 1.3(2) . . . . ?
C22 C21 C26 C25 -1.4(2) . . . . ?
C6 C21 C26 C25 178.24(14) . . . . ?
C24 C25 C26 C21 0.6(2) . . . . ?

N4 C2 N1 C7 -177.01(14) . . . . ?
S3 C2 N1 C7 4.0(2) . . . . ?
N1 C2 N4 N5 5.86(19) . . . . ?
S3 C2 N4 N5 -175.08(10) . . . . ?
C21 C6 N5 N4 -177.95(12) . . . . ?
C2 N4 N5 C6 178.25(13) . . . . ?
C22 C23 O27 C28 8.9(2) . . . . ?
C24 C23 O27 C28 -172.08(14) . . . . ?
C25 C24 O28 C29 5.4(2) . . . . ?
C23 C24 O28 C29 -174.28(12) . . . . ?

```
_refine_diff_density_max    0.219
_refine_diff_density_min   -0.159
_refine_diff_density_rms    0.034

_shelx_res_file
;

    shelx.res created by SHELXL-2014/7


TITL T3_20191008 in P2(1)/c
CELL  0.71073   8.5409   8.1329  18.8922   90.000  102.099   90.000
ZERR     4.00   0.0007   0.0005   0.0012    0.000    0.007    0.000
LATT   1
SYMM  - X, 1/2 + Y, 1/2 - Z
SFAC  C    H    N    O    S
UNIT  44 60 12 8 4
MERG   2
FMAP   2
PLAN   20
ACTA
BOND   $H
CONF
L.S. 40
WGHT    0.042600    0.362100
EXTI    0.014162
FVAR       6.08435
MOLE    1
C2    1    0.999099   -0.120394    0.119315    11.00000    0.03104    0.03278 =
         0.03078   -0.00204    0.00947   -0.00088
C6    1    0.641836    0.100731    0.074110    11.00000    0.03307    0.05051 =
         0.03152   -0.00047    0.01182    0.00452
AFIX  43
H6    2    0.652287    0.122583    0.027002    11.00000   -1.50000
AFIX   0
C7    1    1.117663   -0.261567    0.232090    11.00000    0.06237    0.06610 =
         0.03506    0.00772    0.00955    0.02366
AFIX 137
H7A   2    1.153710   -0.349450    0.205557    11.00000   -1.50000
H7B   2    1.078739   -0.306097    0.272203    11.00000   -1.50000
H7C   2    1.205127   -0.188129    0.249751    11.00000   -1.50000
AFIX   0
C21   1    0.502762    0.167179    0.097949    11.00000    0.03046    0.04284 =
         0.03352   -0.00231    0.00959    0.00281
C22   1    0.478541    0.146542    0.168727    11.00000    0.02816    0.03938 =
         0.03201   -0.00153    0.00609    0.00216
```

```
AFIX  43
H22   2    0.553388    0.089506    0.202759    11.00000   -1.50000
AFIX   0
C23   1    0.343779    0.210897    0.187661    11.00000    0.03196    0.03879 =
        0.03138   -0.00380    0.00986    0.00028
C24   1    0.228111    0.293953    0.135767    11.00000    0.03090    0.03811 =
        0.04075   -0.00332    0.01085    0.00539
C25   1    0.253236    0.315732    0.066726    11.00000    0.04082    0.05854 =
        0.03919    0.00820    0.00895    0.01700
AFIX  43
H25   2    0.178350    0.372329    0.032541    11.00000   -1.50000
AFIX   0
C26   1    0.390267    0.253214    0.048219    11.00000    0.04326    0.06255 =
        0.03431    0.00635    0.01338    0.01216
AFIX  43
H26   2    0.406905    0.269337    0.001619    11.00000   -1.50000
AFIX   0
C28   1    0.427714    0.136765    0.312169    11.00000    0.05374    0.08994 =
        0.03410    0.00954    0.01182    0.01398
AFIX 137
H28A  2    0.451836    0.025259    0.301640    11.00000   -1.50000
H28B  2    0.389313    0.139969    0.356406    11.00000   -1.50000
H28C  2    0.522775    0.202516    0.317283    11.00000   -1.50000
AFIX   0
C29   1   -0.030307    0.417699    0.106122    11.00000    0.03646    0.05474 =
        0.06234    0.00635    0.01086    0.01389
AFIX 137
H29A  2    0.007395    0.516352    0.087317    11.00000   -1.50000
H29B  2   -0.118956    0.443527    0.128101    11.00000   -1.50000
H29C  2   -0.064168    0.340787    0.067392    11.00000   -1.50000
AFIX   0
N1    3    0.990085   -0.172617    0.184969    11.00000    0.03968    0.05023 =
        0.03222    0.00497    0.01451    0.01014
H1    2    0.909968   -0.150916    0.199939    11.00000   -1.50000
N4    3    0.874511   -0.032386    0.082455    11.00000    0.03394    0.05458 =
        0.03015    0.00595    0.01462    0.01075
H4    2    0.875478    0.002624    0.039936    11.00000   -1.50000
N5    3    0.749940    0.014286    0.114316    11.00000    0.02994    0.04337 =
        0.03210   -0.00287    0.01236    0.00341
O27   4    0.308287    0.199673    0.254777    11.00000    0.04009    0.07540 =
        0.03262    0.00340    0.01422    0.01500
O28   4    0.096170    0.346761    0.159149    11.00000    0.03788    0.05868 =
        0.04783    0.00484    0.01639    0.01818
S3    5    1.155254   -0.160892    0.079984    11.00000    0.03397    0.05264 =
        0.04238    0.00832    0.01854    0.00904
HKLF    4

REM  T3_20191008 in P2(1)/c
REM R1 =  0.0313 for    2366 Fo > 4sig(Fo)  and  0.0362 for all    2666 data
REM    164 parameters refined using      0 restraints

END

WGHT      0.0424      0.3629

REM Highest difference peak  0.219,  deepest hole -0.159,  1-sigma level  0.034
Q1    1   0.4927  0.1595  0.1325  11.00000  0.05    0.22
```

```
Q2    1   1.0874 -0.1043  0.1085  11.00000  0.05    0.20
Q3    1   0.5510  0.1229  0.0843  11.00000  0.05    0.19
Q4    1   0.4294  0.1742  0.0695  11.00000  0.05    0.18
Q5    1   0.3886  0.1550  0.1742  11.00000  0.05    0.18
Q6    1   0.2311  0.2859  0.1019  11.00000  0.05    0.17
Q7    1   0.3100  0.2990  0.1685  11.00000  0.05    0.17
Q8    1   0.2589  0.3260  0.1092  11.00000  0.05    0.16
Q9    1   0.4690  0.2316  0.0827  11.00000  0.05    0.16
Q10   1   0.4187  0.2131  0.1838  11.00000  0.05    0.16
Q11   1   0.3236  0.2715  0.0606  11.00000  0.05    0.15
Q12   1   0.3435  0.3116  0.0683  11.00000  0.05    0.14
Q13   1   0.6600  0.1877  0.0228  11.00000  0.05    0.14
Q14   1   0.9991 -0.1430  0.1495  11.00000  0.05    0.14
Q15   1   1.2655 -0.1819  0.1230  11.00000  0.05    0.14
Q16   1   1.2364 -0.2546  0.1108  11.00000  0.05    0.14
Q17   1   0.3341  0.1989  0.2100  11.00000  0.05    0.13
Q18   1   0.3390  0.2816  0.2475  11.00000  0.05    0.13
Q19   1   1.0569 -0.1689  0.0389  11.00000  0.05    0.13
Q20   1   0.4457  0.3191  0.0009  11.00000  0.05    0.13
;
```

CIF for T5

```
data_shelx

_audit_creation_method            'SHELXL-2014/7'
_shelx_SHELXL_version_number      '2014/7'
_chemical_name_systematic         ?
_chemical_name_common             ?
_chemical_melting_point           ?
_chemical_formula_moiety          'C9 H9 Cl2 N3 S'
_chemical_formula_sum             'C9 H9 Cl2 N3 S'
_chemical_formula_weight          262.15

loop_
 _atom_type_symbol
 _atom_type_description
 _atom_type_scat_dispersion_real
 _atom_type_scat_dispersion_imag
 _atom_type_scat_source
 'C'  'C'   0.0033   0.0016
 'International Tables Vol C Tables 4.2.6.8 and 6.1.1.4'
 'H'  'H'   0.0000   0.0000
 'International Tables Vol C Tables 4.2.6.8 and 6.1.1.4'
 'N'  'N'   0.0061   0.0033
 'International Tables Vol C Tables 4.2.6.8 and 6.1.1.4'
 'S'  'S'   0.1246   0.1234
 'International Tables Vol C Tables 4.2.6.8 and 6.1.1.4'
 'Cl'  'Cl'   0.1484   0.1585
 'International Tables Vol C Tables 4.2.6.8 and 6.1.1.4'

_space_group_crystal_system       monoclinic
_space_group_IT_number            15
_space_group_name_H-M_alt         'C 2/c'
_space_group_name_Hall            '-C 2yc'
```

_shelx_space_group_comment
;
The symmetry employed for this shelxl refinement is uniquely defined
by the following loop, which should always be used as a source of
symmetry information in preference to the above space-group names.
They are only intended as comments.
;

loop_
 _space_group_symop_operation_xyz
 'x, y, z'
 '-x, y, -z+1/2'
 'x+1/2, y+1/2, z'
 '-x+1/2, y+1/2, -z+1/2'
 '-x, -y, -z'
 'x, -y, z-1/2'
 '-x+1/2, -y+1/2, -z'
 'x+1/2, -y+1/2, z-1/2'

_cell_length_a   12.8992(10)
_cell_length_b   10.0640(8)
_cell_length_c   18.4826(13)
_cell_angle_alpha   90
_cell_angle_beta   96.754(7)
_cell_angle_gamma   90
_cell_volume   2382.7(3)
_cell_formula_units_Z   8
_cell_measurement_temperature   296(2)
_cell_measurement_reflns_used   754
_cell_measurement_theta_min   3.1440
_cell_measurement_theta_max   27.3160

_exptl_crystal_description   block
_exptl_crystal_colour   colourless
_exptl_crystal_density_meas   ?
_exptl_crystal_density_method   ?
_exptl_crystal_density_diffrn   1.462
_exptl_crystal_F_000   1072
_exptl_transmission_factor_min   ?
_exptl_transmission_factor_max   ?
_exptl_crystal_size_max   0.5
_exptl_crystal_size_mid   0.4
_exptl_crystal_size_min   0.3
_exptl_absorpt_coefficient_mu   0.690
_shelx_estimated_absorpt_T_min   ?
_shelx_estimated_absorpt_T_max   ?
_exptl_absorpt_correction_T_min   0.24257
_exptl_absorpt_correction_T_max   1.00000
_exptl_absorpt_correction_type   multi-scan
_exptl_absorpt_process_details
;
CrysAlisPro 1.171.40.53 (Rigaku Oxford Diffraction, 2019)
Empirical absorption correction using spherical harmonics,
 implemented in SCALE3 ABSPACK scaling algorithm.
;
_exptl_absorpt_special_details   ?

_diffrn_ambient_temperature 296(2)
_diffrn_radiation_wavelength 0.71073
_diffrn_radiation_type MoK\a
_diffrn_source ?
_diffrn_measurement_device_type 'KM4 CCD four-circle diffractometer'
_diffrn_measurement_method ?
_diffrn_detector_area_resol_mean ?
_diffrn_reflns_number 6897
_diffrn_reflns_av_unetI/netI 0.0204
_diffrn_reflns_av_R_equivalents 0.0187
_diffrn_reflns_limit_h_min -15
_diffrn_reflns_limit_h_max 15
_diffrn_reflns_limit_k_min -8
_diffrn_reflns_limit_k_max 12
_diffrn_reflns_limit_l_min -18
_diffrn_reflns_limit_l_max 22
_diffrn_reflns_theta_min 2.219
_diffrn_reflns_theta_max 28.065
_diffrn_reflns_theta_full 25.242
_diffrn_measured_fraction_theta_max 0.860
_diffrn_measured_fraction_theta_full 1.000
_diffrn_reflns_Laue_measured_fraction_max 0.860
_diffrn_reflns_Laue_measured_fraction_full 1.000
_diffrn_reflns_point_group_measured_fraction_max 0.860
_diffrn_reflns_point_group_measured_fraction_full 1.000
_reflns_number_total 2493
_reflns_number_gt 1981
_reflns_threshold_expression 'I > 2\s(I)'
_reflns_Friedel_coverage 0.000
_reflns_Friedel_fraction_max .
_reflns_Friedel_fraction_full .

_reflns_special_details
;
Reflections were merged by SHELXL according to the crystal
class for the calculation of statistics and refinement.

_reflns_Friedel_fraction is defined as the number of unique
Friedel pairs measured divided by the number that would be
possible theoretically, ignoring centric projections and
systematic absences.
;

_computing_data_collection
;
CrysAlisPro, Agilent Technologies,
Version 1.171.37.35h (release 09-02-2015 CrysAlis171 .NET)
(compiled Feb 9 2015,16:26:32)
;
_computing_cell_refinement
;
CrysAlisPro, Agilent Technologies,
Version 1.171.37.35h (release 09-02-2015 CrysAlis171 .NET)
(compiled Feb 9 2015,16:26:32)
;
_computing_data_reduction
;

CrysAlisPro, Agilent Technologies,
Version 1.171.37.35h (release 09-02-2015 CrysAlis171 .NET)
(compiled Feb 9 2015,16:26:32)
;
_computing_structure_solution 'SHELXS-2013/1 (Sheldrick, 2013)'
_computing_structure_refinement 'SHELXL-2014/7 (Sheldrick, 2014)'
_computing_molecular_graphics ?
_computing_publication_material ?
_refine_special_details ?
_refine_ls_structure_factor_coef Fsqd
_refine_ls_matrix_type full
_refine_ls_weighting_scheme calc
_refine_ls_weighting_details
'w=1/[\s^2^(Fo^2^)+(0.0665P)^2^+2.3540P] where P=(Fo^2^+2Fc^2^)/3'
_atom_sites_solution_primary refxyz
_atom_sites_solution_secondary ?
_atom_sites_solution_hydrogens mixed
_refine_ls_hydrogen_treatment mixed
_refine_ls_extinction_method none
_refine_ls_extinction_coef .
_refine_ls_number_reflns 2493
_refine_ls_number_parameters 143
_refine_ls_number_restraints 0
_refine_ls_R_factor_all 0.0568
_refine_ls_R_factor_gt 0.0439
_refine_ls_wR_factor_ref 0.1379
_refine_ls_wR_factor_gt 0.1266
_refine_ls_goodness_of_fit_ref 1.066
_refine_ls_restrained_S_all 1.066
_refine_ls_shift/su_max 0.001
_refine_ls_shift/su_mean 0.000

loop_
_atom_site_label
_atom_site_type_symbol
_atom_site_fract_x
_atom_site_fract_y
_atom_site_fract_z
_atom_site_U_iso_or_equiv
_atom_site_adp_type
_atom_site_occupancy
_atom_site_site_symmetry_order
_atom_site_calc_flag
_atom_site_refinement_flags_posn
_atom_site_refinement_flags_adp
_atom_site_refinement_flags_occupancy
_atom_site_disorder_assembly
_atom_site_disorder_group
C2 C 0.16570(19) 0.9349(3) 0.74732(13) 0.0479(6) Uani 1 1 d . . . . .
C6 C 0.07636(19) 0.8279(3) 0.90895(13) 0.0500(6) Uani 1 1 d . . . . .
H6 H 0.0247 0.8922 0.9098 0.075 Uiso 1 1 calc R U . . .
C7 C 0.3129(3) 0.8474(4) 0.68877(18) 0.0766(9) Uani 1 1 d . . . . .
H7A H 0.2744 0.8420 0.6411 0.115 Uiso 1 1 calc R U . . .
H7B H 0.3593 0.7729 0.6962 0.115 Uiso 1 1 calc R U . . .
H7C H 0.3526 0.9283 0.6928 0.115 Uiso 1 1 calc R U . . .
C21 C 0.09089(18) 0.7301(2) 0.96791(13) 0.0456(5) Uani 1 1 d . . . . .
C22 C 0.16299(19) 0.6274(3) 0.96739(13) 0.0474(6) Uani 1 1 d . . . . .

```
H22 H 0.2045 0.6206 0.9296 0.071 Uiso 1 1 calc R U . . .
C23 C 0.1726(2) 0.5352(3) 1.02344(14) 0.0514(6) Uani 1 1 d . . . . .
C24 C 0.1105(2) 0.5437(3) 1.07997(14) 0.0550(7) Uani 1 1 d . . . . .
C25 C 0.0411(2) 0.6462(4) 1.08106(15) 0.0660(8) Uani 1 1 d . . . . .
H25 H 0.0007 0.6537 1.1194 0.099 Uiso 1 1 calc R U . . .
C26 C 0.0306(2) 0.7386(3) 1.02553(15) 0.0615(7) Uani 1 1 d . . . . .
H26 H -0.0173 0.8074 1.0266 0.092 Uiso 1 1 calc R U . . .
N1 N 0.24033(19) 0.8462(2) 0.74377(13) 0.0563(6) Uani 1 1 d . . . . .
H1 H 0.252(3) 0.793(4) 0.7728(19) 0.085 Uiso 1 1 d . U . . .
N4 N 0.11103(18) 0.9260(2) 0.80540(13) 0.0534(6) Uani 1 1 d . . . . .
H4 H 0.064(3) 0.974(4) 0.809(2) 0.080 Uiso 1 1 d . U . . .
N5 N 0.13225(15) 0.8277(2) 0.85666(11) 0.0477(5) Uani 1 1 d . . . . .
S3 S 0.13423(6) 1.05573(7) 0.68487(4) 0.0590(2) Uani 1 1 d . . . . .
Cl23 Cl 0.26246(8) 0.40935(8) 1.02152(5) 0.0844(3) Uani 1 1 d . . . . .
Cl24 Cl 0.11927(8) 0.42480(9) 1.14854(4) 0.0825(3) Uani 1 1 d . . . . .

loop_
 _atom_site_aniso_label
 _atom_site_aniso_U_11
 _atom_site_aniso_U_22
 _atom_site_aniso_U_33
 _atom_site_aniso_U_23
 _atom_site_aniso_U_13
 _atom_site_aniso_U_12
C2 0.0512(13) 0.0475(14) 0.0443(13) 0.0008(10) 0.0036(10) -0.0135(11)
C6 0.0466(13) 0.0515(15) 0.0521(14) 0.0039(11) 0.0065(11) 0.0035(11)
C7 0.087(2) 0.080(2) 0.0699(19) 0.0020(17) 0.0361(17) 0.0058(18)
C21 0.0420(12) 0.0499(14) 0.0443(12) 0.0019(10) 0.0031(9) -0.0032(10)
C22 0.0509(13) 0.0480(14) 0.0435(13) -0.0031(11) 0.0062(10) -0.0035(11)
C23 0.0576(14) 0.0426(13) 0.0513(14) -0.0045(11) -0.0041(11) -0.0020(11)
C24 0.0588(15) 0.0628(17) 0.0414(14) 0.0043(12) -0.0031(11) -0.0187(13)
C25 0.0596(16) 0.093(2) 0.0473(15) 0.0061(15) 0.0160(12) -0.0056(16)
C26 0.0516(15) 0.074(2) 0.0603(17) 0.0052(14) 0.0135(12) 0.0072(13)
N1 0.0652(14) 0.0538(14) 0.0520(13) 0.0053(10) 0.0149(11) -0.0013(11)
N4 0.0517(12) 0.0561(14) 0.0529(13) 0.0126(10) 0.0078(10) 0.0051(10)
N5 0.0469(11) 0.0482(12) 0.0473(11) 0.0070(9) 0.0029(9) -0.0017(9)
S3 0.0660(4) 0.0533(4) 0.0566(4) 0.0129(3) 0.0023(3) -0.0137(3)
Cl23 0.1153(7) 0.0582(5) 0.0789(6) 0.0015(4) 0.0077(5) 0.0310(4)
Cl24 0.1014(6) 0.0856(6) 0.0561(5) 0.0224(4) -0.0099(4) -0.0293(5)

_geom_special_details
;
 All esds (except the esd in the dihedral angle between two l.s. planes)
 are estimated using the full covariance matrix.  The cell esds are taken
 into account individually in the estimation of esds in distances, angles
 and torsion angles; correlations between esds in cell parameters are only
 used when they are defined by crystal symmetry.  An approximate (isotropic)
 treatment of cell esds is used for estimating esds involving l.s. planes.
;

loop_
 _geom_bond_atom_site_label_1
 _geom_bond_atom_site_label_2
 _geom_bond_distance
 _geom_bond_site_symmetry_2
 _geom_bond_publ_flag
C2 N1 1.320(4) . ?
```

C2 N4 1.355(3) . ?
C2 S3 1.693(3) . ?
C6 N5 1.272(3) . ?
C6 C21 1.464(3) . ?
C6 H6 0.9300 . ?
C7 N1 1.461(4) . ?
C7 H7A 0.9600 . ?
C7 H7B 0.9600 . ?
C7 H7C 0.9600 . ?
C21 C22 1.391(4) . ?
C21 C26 1.393(4) . ?
C22 C23 1.385(4) . ?
C22 H22 0.9300 . ?
C23 C24 1.392(4) . ?
C23 Cl23 1.720(3) . ?
C24 C25 1.368(4) . ?
C24 Cl24 1.737(3) . ?
C25 C26 1.379(4) . ?
C25 H25 0.9300 . ?
C26 H26 0.9300 . ?
N1 H1 0.76(4) . ?
N4 N5 1.375(3) . ?
N4 H4 0.79(4) . ?

loop_
 _geom_angle_atom_site_label_1
 _geom_angle_atom_site_label_2
 _geom_angle_atom_site_label_3
 _geom_angle
 _geom_angle_site_symmetry_1
 _geom_angle_site_symmetry_3
 _geom_angle_publ_flag
N1 C2 N4 116.5(2) . . ?
N1 C2 S3 124.7(2) . . ?
N4 C2 S3 118.8(2) . . ?
N5 C6 C21 122.0(2) . . ?
N5 C6 H6 119.0 . . ?
C21 C6 H6 119.0 . . ?
N1 C7 H7A 109.5 . . ?
N1 C7 H7B 109.5 . . ?
H7A C7 H7B 109.5 . . ?
N1 C7 H7C 109.5 . . ?
H7A C7 H7C 109.5 . . ?
H7B C7 H7C 109.5 . . ?
C22 C21 C26 119.0(2) . . ?
C22 C21 C6 121.4(2) . . ?
C26 C21 C6 119.6(2) . . ?
C23 C22 C21 119.6(2) . . ?
C23 C22 H22 120.2 . . ?
C21 C22 H22 120.2 . . ?
C22 C23 C24 120.7(2) . . ?
C22 C23 Cl23 118.6(2) . . ?
C24 C23 Cl23 120.6(2) . . ?
C25 C24 C23 119.6(2) . . ?
C25 C24 Cl24 119.7(2) . . ?
C23 C24 Cl24 120.8(2) . . ?
C24 C25 C26 120.2(3) . . ?

C24 C25 H25 119.9 . . ?
C26 C25 H25 119.9 . . ?
C25 C26 C21 120.9(3) . . ?
C25 C26 H26 119.6 . . ?
C21 C26 H26 119.6 . . ?
C2 N1 C7 124.0(3) . . ?
C2 N1 H1 121(3) . . ?
C7 N1 H1 114(3) . . ?
C2 N4 N5 120.9(2) . . ?
C2 N4 H4 120(3) . . ?
N5 N4 H4 119(3) . . ?
C6 N5 N4 116.0(2) . . ?

loop_
 _geom_torsion_atom_site_label_1
 _geom_torsion_atom_site_label_2
 _geom_torsion_atom_site_label_3
 _geom_torsion_atom_site_label_4
 _geom_torsion
 _geom_torsion_site_symmetry_1
 _geom_torsion_site_symmetry_2
 _geom_torsion_site_symmetry_3
 _geom_torsion_site_symmetry_4
 _geom_torsion_publ_flag
N5 C6 C21 C22 -3.6(4) . . . . ?
N5 C6 C21 C26 177.1(3) . . . . ?
C26 C21 C22 C23 0.7(4) . . . . ?
C6 C21 C22 C23 -178.6(2) . . . . ?
C21 C22 C23 C24 0.4(4) . . . . ?
C21 C22 C23 Cl23 -179.92(19) . . . . ?
C22 C23 C24 C25 -1.6(4) . . . . ?
Cl23 C23 C24 C25 178.7(2) . . . . ?
C22 C23 C24 Cl24 177.68(19) . . . . ?
Cl23 C23 C24 Cl24 -2.0(3) . . . . ?
C23 C24 C25 C26 1.7(4) . . . . ?
Cl24 C24 C25 C26 -177.6(2) . . . . ?
C24 C25 C26 C21 -0.6(4) . . . . ?
C22 C21 C26 C25 -0.6(4) . . . . ?
C6 C21 C26 C25 178.6(3) . . . . ?
N4 C2 N1 C7 -174.5(3) . . . . ?
S3 C2 N1 C7 5.9(4) . . . . ?
N1 C2 N4 N5 -1.1(4) . . . . ?
S3 C2 N4 N5 178.55(18) . . . . ?
C21 C6 N5 N4 -179.3(2) . . . . ?
C2 N4 N5 C6 -179.8(2) . . . . ?

_refine_diff_density_max   0.559
_refine_diff_density_min   -0.344
_refine_diff_density_rms   0.048

_shelx_res_file
;

    shelx.res created by SHELXL-2014/7

TITL T5_20191015 in C2/c

```
CELL  0.71073  12.8992  10.0640  18.4826   90.000   96.754   90.000
ZERR    8.00   0.0010   0.0008   0.0013    0.000    0.007    0.000
LATT   7
SYMM  - X,   Y, 1/2 - Z
SFAC  C    H    N    S    CL
UNIT  72 72 24 8 16
MERG   2
FMAP   2
PLAN   -10
ACTA
BOND   $H
CONF
L.S.  30
WGHT    0.066500    2.354000
FVAR       2.52979
MOLE    1
C2    1    0.165703    0.934857    0.747320    11.00000    0.05125    0.04754 =
        0.04427    0.00079    0.00358   -0.01352
C6    1    0.076356    0.827928    0.908951    11.00000    0.04660    0.05149 =
        0.05210    0.00386    0.00645    0.00346
AFIX  43
H6    2    0.024689    0.892181    0.909764    11.00000   -1.50000
AFIX   0
C7    1    0.312882    0.847437    0.688771    11.00000    0.08681    0.07955 =
        0.06986    0.00203    0.03614    0.00584
AFIX 137
H7A   2    0.274412    0.841998    0.641137    11.00000   -1.50000
H7B   2    0.359308    0.772853    0.696217    11.00000   -1.50000
H7C   2    0.352618    0.928302    0.692811    11.00000   -1.50000
AFIX   0
C21   1    0.090886    0.730084    0.967914    11.00000    0.04203    0.04995 =
        0.04426    0.00188    0.00311   -0.00316
C22   1    0.162991    0.627359    0.967388    11.00000    0.05085    0.04797 =
        0.04348   -0.00307    0.00621   -0.00346
AFIX  43
H22   2    0.204452    0.620620    0.929649    11.00000   -1.50000
AFIX   0
C23   1    0.172617    0.535202    1.023439    11.00000    0.05765    0.04263 =
        0.05133   -0.00454   -0.00407   -0.00198
C24   1    0.110514    0.543726    1.079967    11.00000    0.05875    0.06278 =
        0.04145    0.00433   -0.00306   -0.01872
C25   1    0.041052    0.646234    1.081056    11.00000    0.05964    0.09346 =
        0.04731    0.00607    0.01597   -0.00564
AFIX  43
H25   2    0.000706    0.653685    1.119379    11.00000   -1.50000
AFIX   0
C26   1    0.030639    0.738559    1.025534    11.00000    0.05156    0.07427 =
        0.06035    0.00520    0.01354    0.00725
AFIX  43
H26   2   -0.017255    0.807403    1.026580    11.00000   -1.50000
AFIX   0
N1    3    0.240326    0.846156    0.743769    11.00000    0.06521    0.05378 =
        0.05196    0.00532    0.01486   -0.00133
H1    2    0.251674    0.792910    0.772806    11.00000   -1.50000
N4    3    0.111026    0.926031    0.805404    11.00000    0.05172    0.05610 =
        0.05291    0.01258    0.00781    0.00511
H4    2    0.063833    0.974032    0.808952    11.00000   -1.50000
```

```
N5    3    0.132246    0.827714    0.856657    11.00000    0.04695    0.04824 =
         0.04733    0.00698    0.00292   -0.00167
S3    4    0.134225    1.055731    0.684875    11.00000    0.06602    0.05328 =
         0.05664    0.01286    0.00232   -0.01371
CL23  5    0.262456    0.409351    1.021518    11.00000    0.11529    0.05822 =
         0.07886    0.00149    0.00765    0.03099
CL24  5    0.119271    0.424798    1.148541    11.00000    0.10141    0.08560 =
         0.05606    0.02240   -0.00990   -0.02930
HKLF    4

REM  T5_20191015 in C2/c
REM R1 =  0.0439 for    1981 Fo > 4sig(Fo)  and  0.0568 for all    2493 data
REM    143 parameters refined using      0 restraints

END

WGHT      0.0653      2.4399

REM Highest difference peak  0.559,  deepest hole -0.344,  1-sigma level  0.048
Q1    1   0.0130  0.5889  1.1483  11.00000  0.05    0.56
Q2    1   0.1869  0.3654  1.1163  11.00000  0.05    0.53
Q3    1   0.0606  0.5799  1.0632  11.00000  0.05    0.21
Q4    1   0.0958  1.0978  0.7081  11.00000  0.05    0.21
Q5    1   0.1240  0.6857  0.9687  11.00000  0.05    0.19
Q6    1   0.0841  0.7772  0.9407  11.00000  0.05    0.19
Q7    1   0.0612  0.4755  1.1436  11.00000  0.05    0.19
Q8    1   0.1447  0.5058  1.0579  11.00000  0.05    0.16
Q9    1   0.1017  0.5188  1.0972  11.00000  0.05    0.15
Q10   1   0.2041  0.4886  1.0169  11.00000  0.05    0.15
;
```

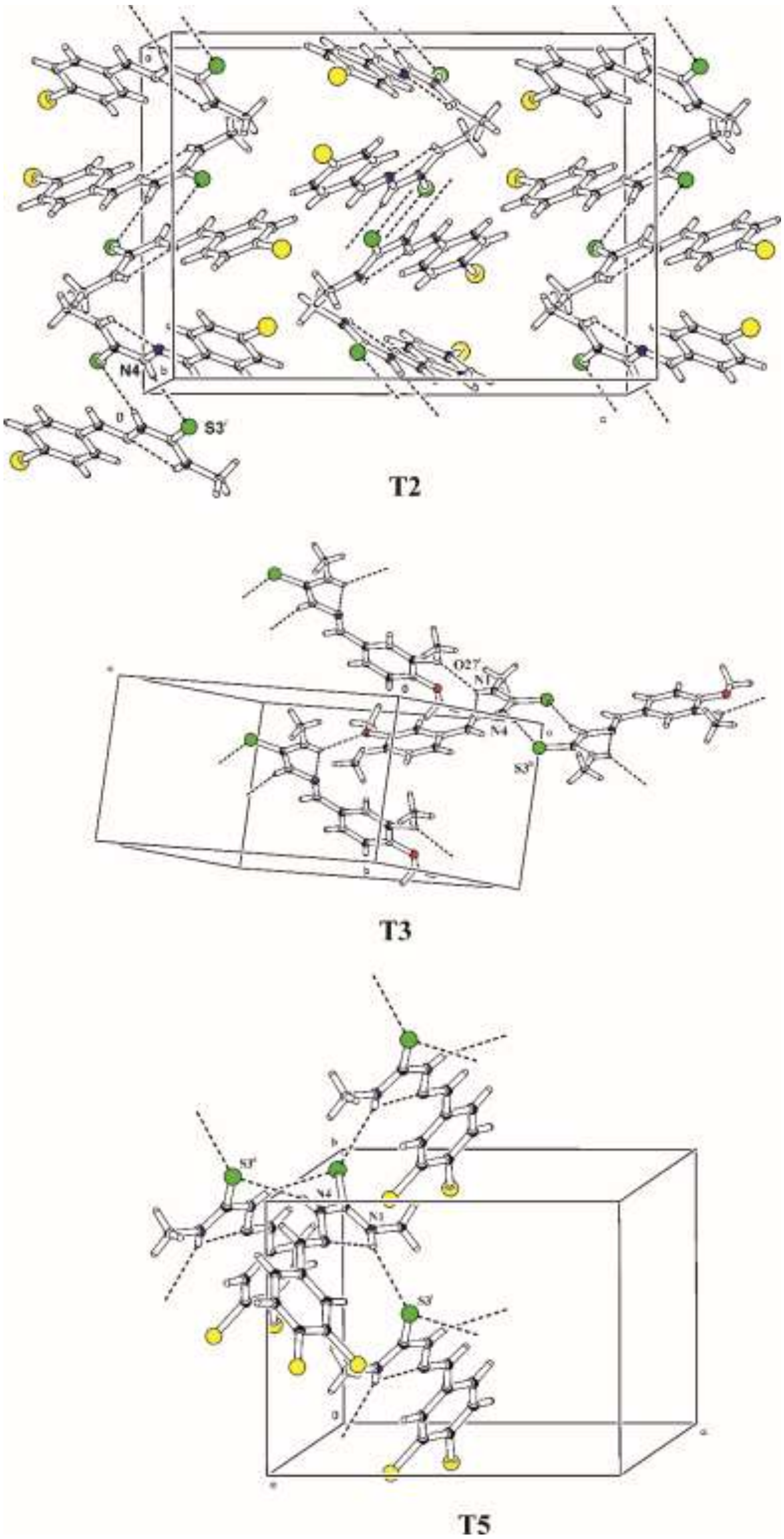

Figure 1S. The molecular packing of **T2, T3** and **T5**. Dashed lines indicate intermolecular hydrogen bonds; **T2** *i* = 1-*x*, -*y*, 1-*z*; **T3:** *i* = 1-*x*, -1/2+*y*, 1/2-*z*; *ii* = 2-*x*, -*y*, -*z*; **T5**: *i* = 1/2-*x*, -1/2+*y*, 3/2-*z*; *ii* = -*x*, *y*, 2-*z*.

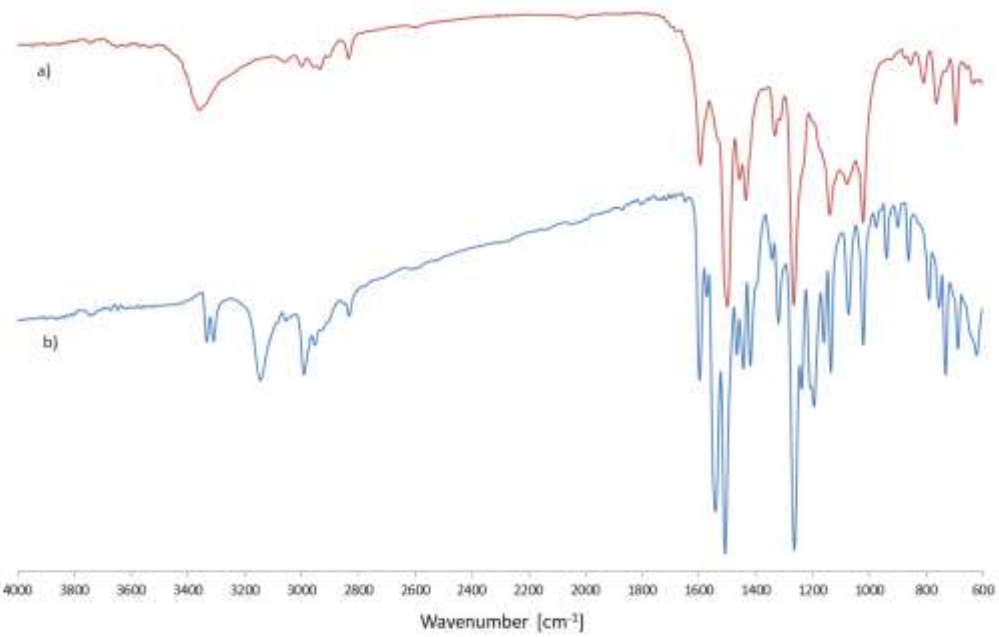

Figure 2S. Infrared spectra of a) $Cu(T1)Cl_2$ and b) T1

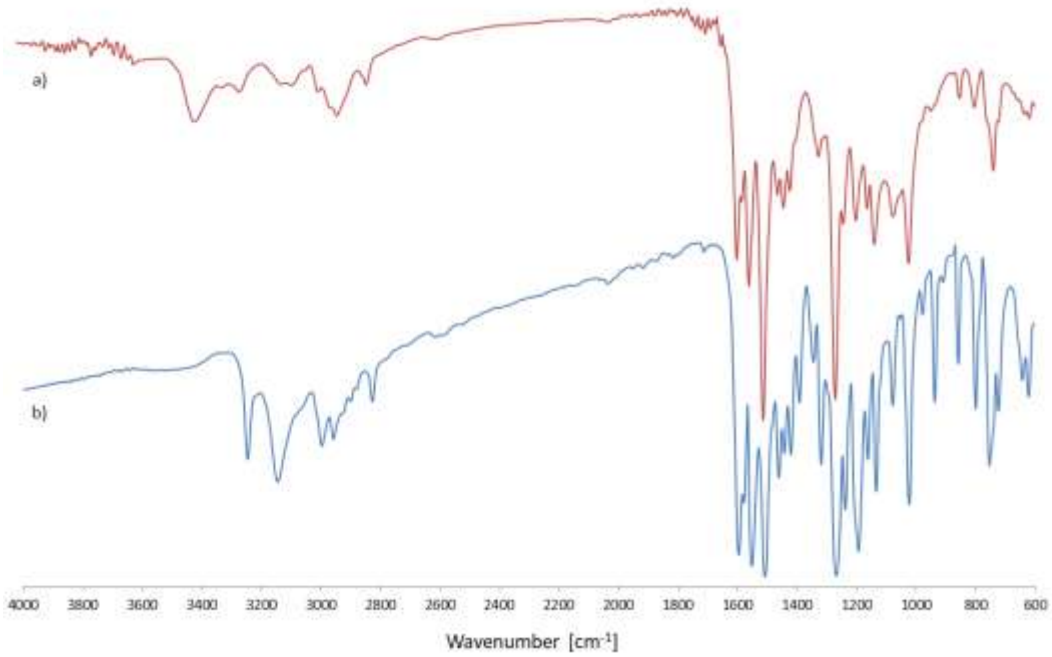

Figure 3S. Infrared spectra of a) $Cu(T10)_2Cl_2$ and b) T10

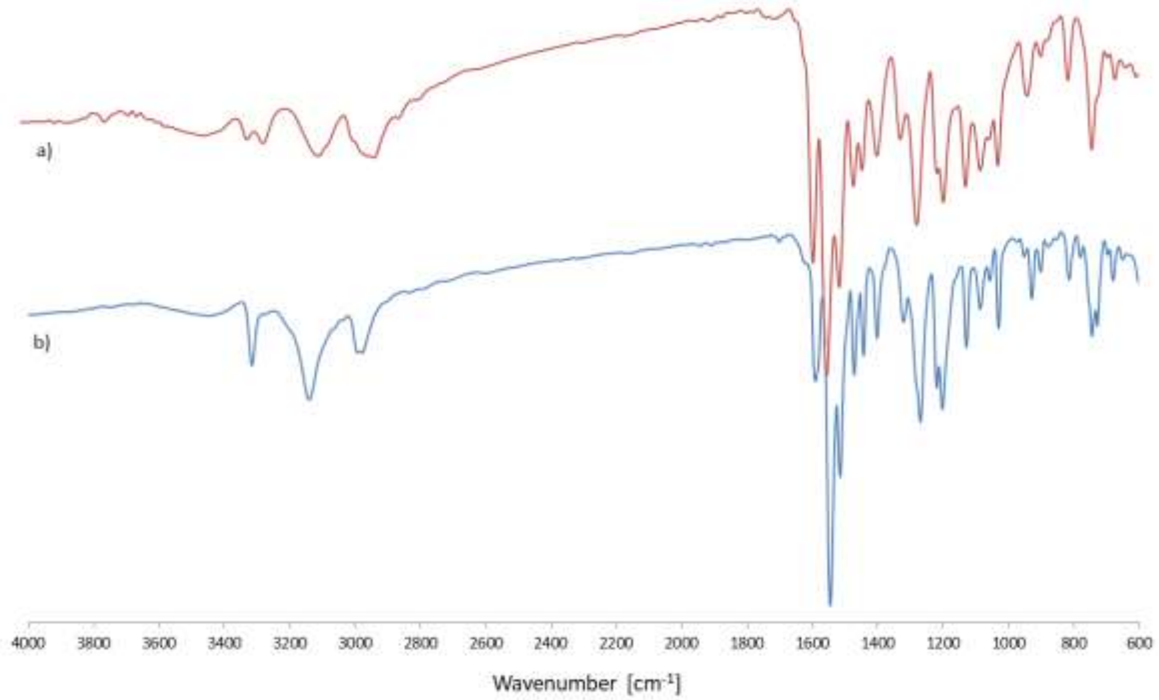

Figure 4S. Infrared spectra of a) $Cu(T12)_2Cl_2$ and b) T12

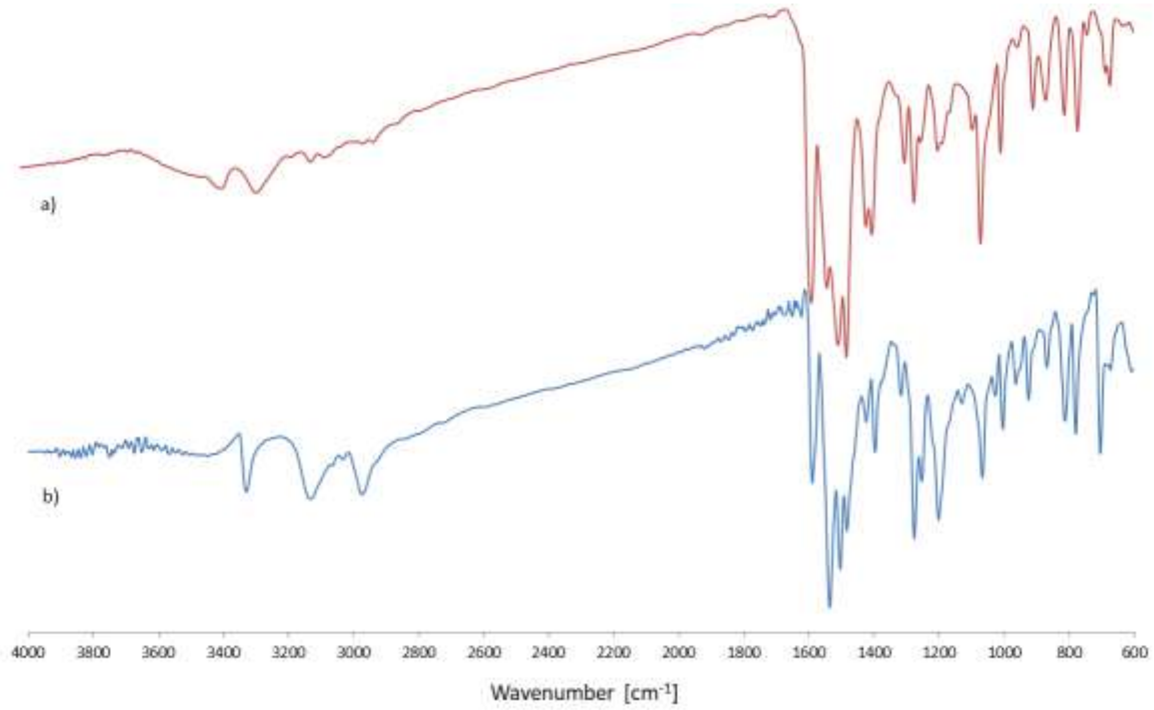

Figure 5S. Infrared spectra of a) $Cu(T13)Cl_2$ and b) T13

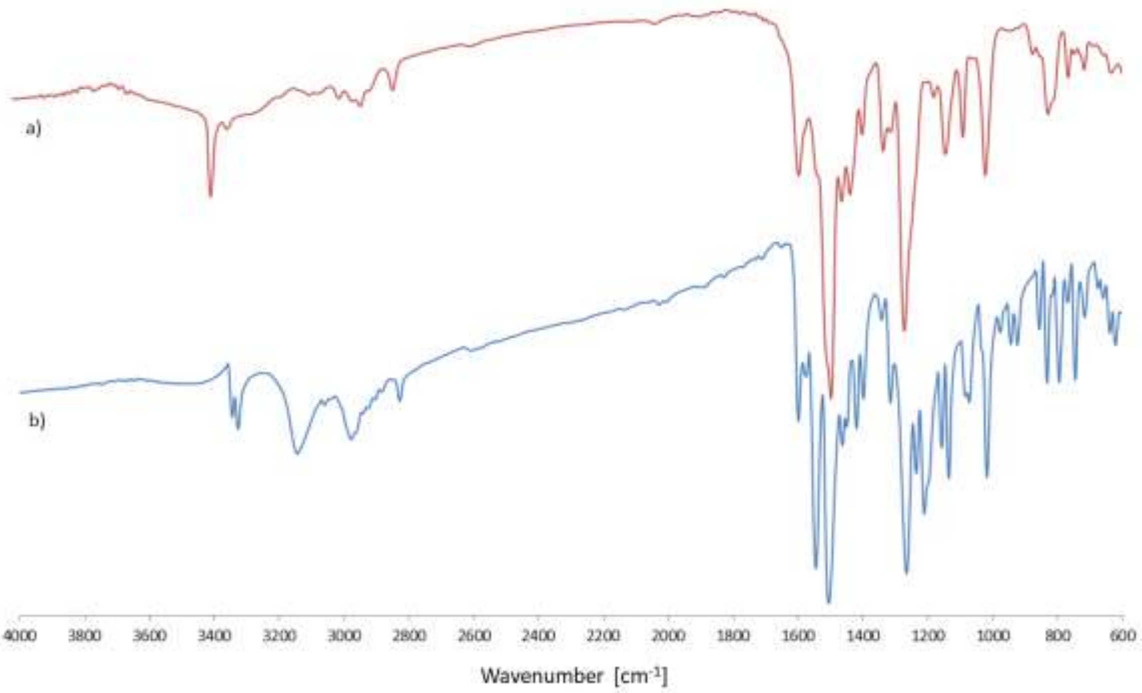

Figure 6S. Infrared spectra of a) $Cu(T16)Cl_2$ and b) T16

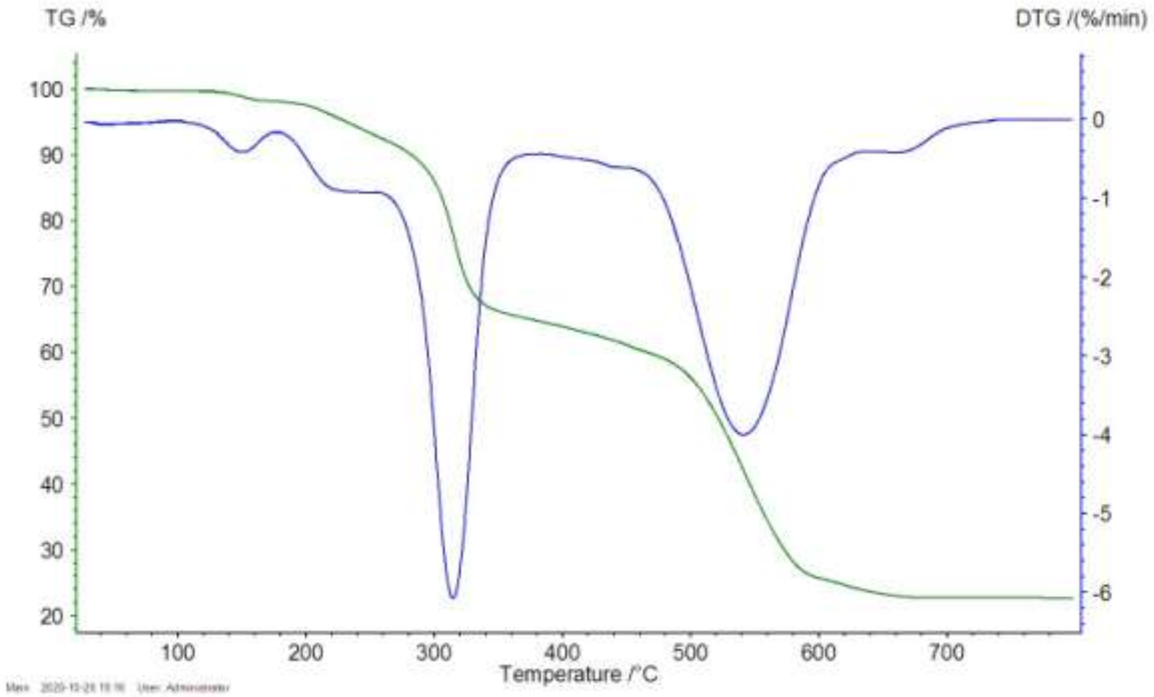

Figure 7S. TG and DTG curves of $Cu(T1)Cl_2$ in air atmosphere

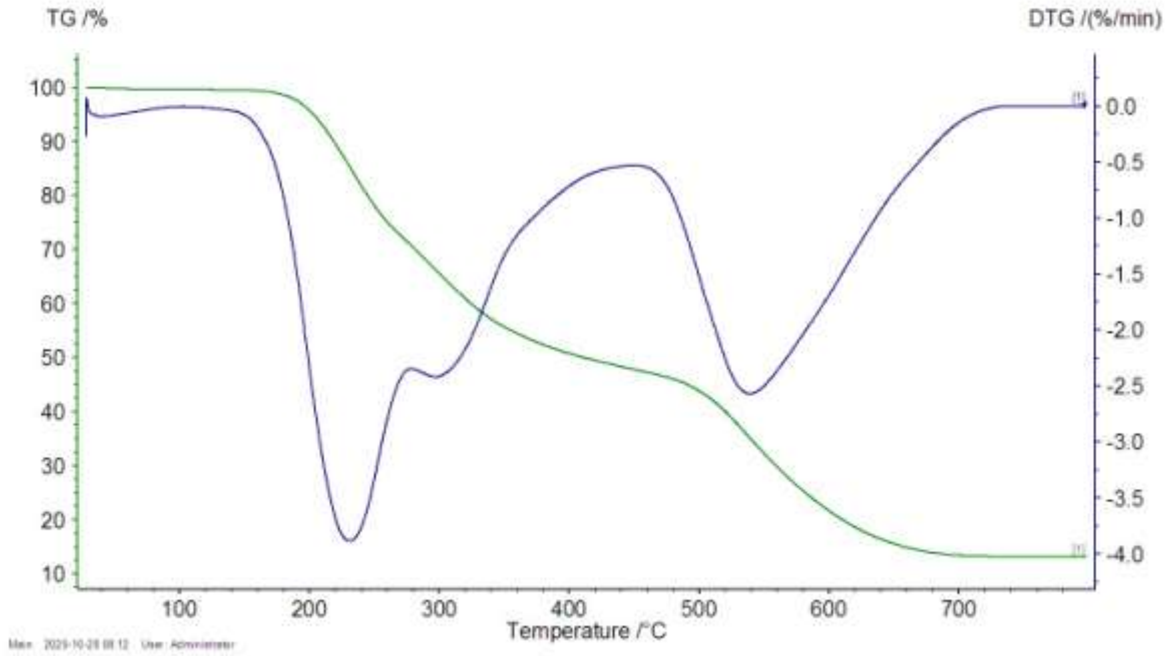

Figure 8S. TG and DTG curves of $Cu(T10)_2Cl_2$ in air atmosphere

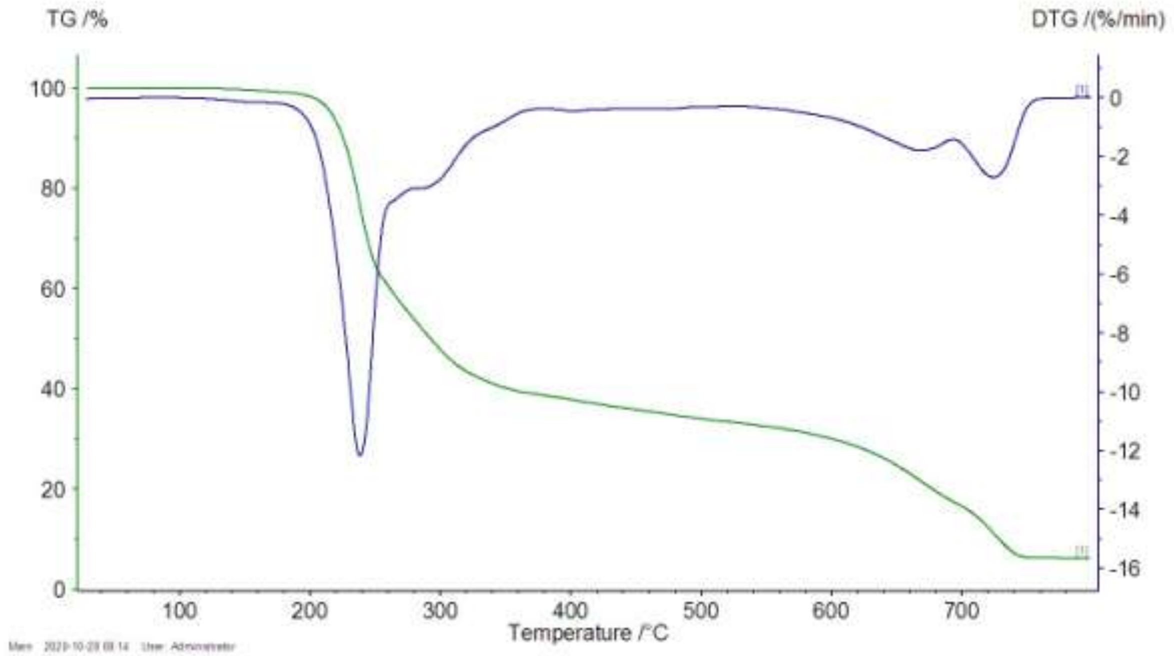

Figure 9S. TG and DTG curves of $Cu(T12)_2Cl_2$ in air atmosphere

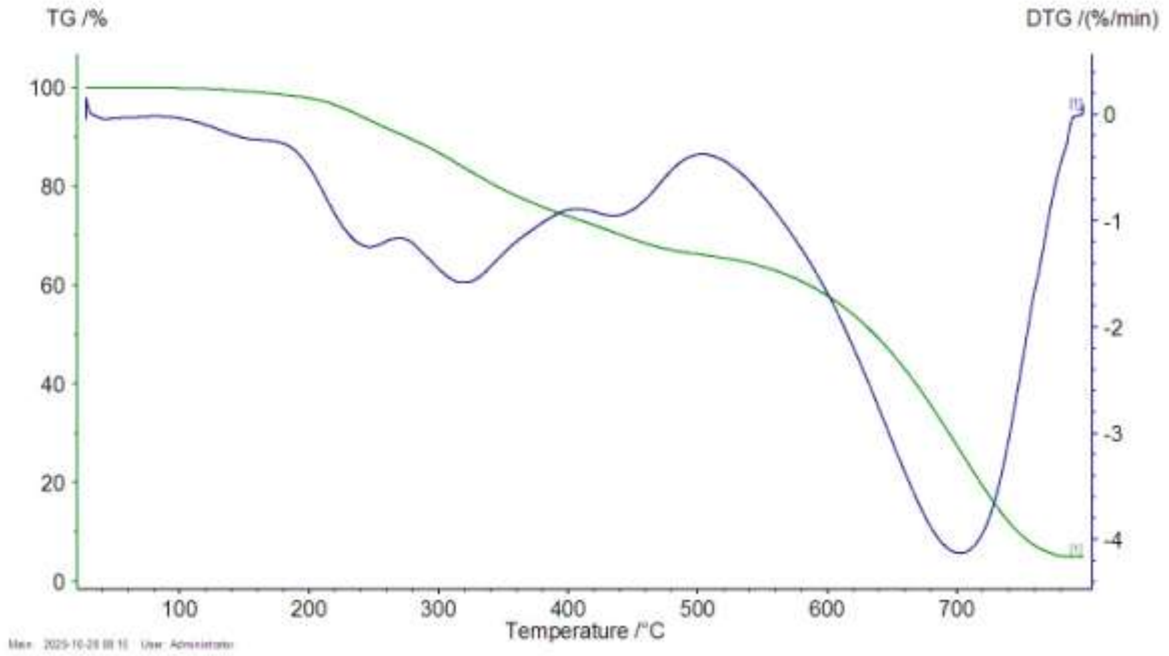

Figure 10S. TG and DTG curves of $Cu(T13)Cl_2$ in air atmosphere

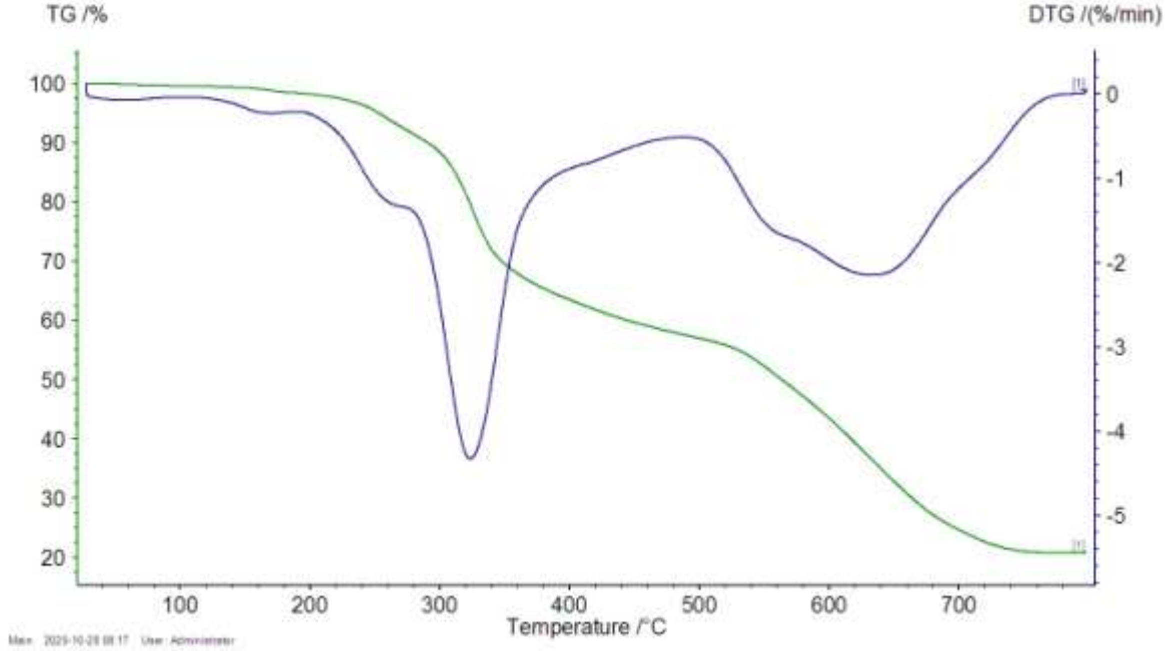

Figure 11S. TG and DTG curves of $Cu(T16)Cl_2$ in air atmosphere

Table 1S. Spectrometry mass (MS) for new compounds.

LP.	Compound structure	Compound formula, thoretical and measured monoisotopic mass, and mass difference in ppm	Theoretical monoisotopic mass of protonated ion and acquired LC/QTOF HRMS spectra
T3	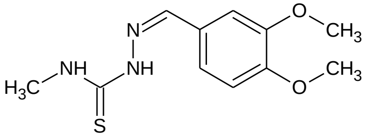<br>M=253,32	C11 H15 N3 O2S<br>Calculated monoisotopic mass: 253.0885<br><br>Measured monoisotopic mass: 253.0889<br><br>Mass error: 1.55 ppm	Theoretical monoisotopic mass of $[M+H]^+$: 254.0958<br>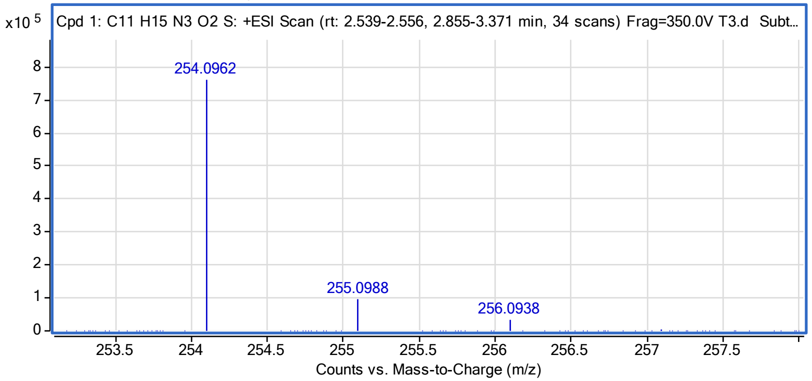
T4	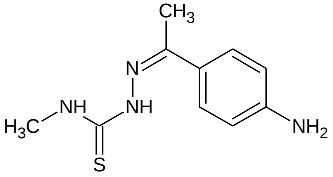<br>M=223,31	C10H14N4S<br>Calculated monoisotopic mass: 222.0939<br><br>Measured monoisotopic mass: 222.0944<br><br>Mass error: 2.14 ppm	Theoretical monoisotopic mass of $[M+H]^+$: 223.1012<br>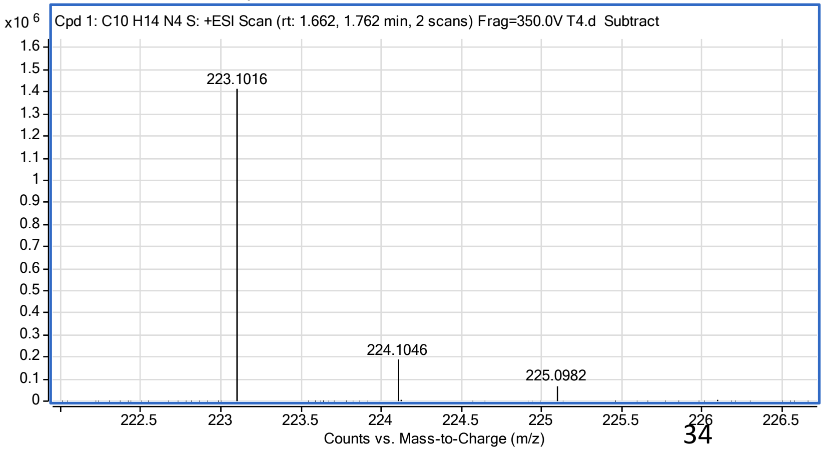

T5	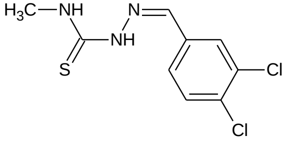<br>M=262,26	C9H9N3SCl2<br>Calculated monoisotopic mass: 260.9894<br><br>Measured monoisotopic mass: 260.9896<br><br>Mass error: 0.55 ppm	Theoretical monoisotopic mass of $[M+H]^+$: 261.9967<br>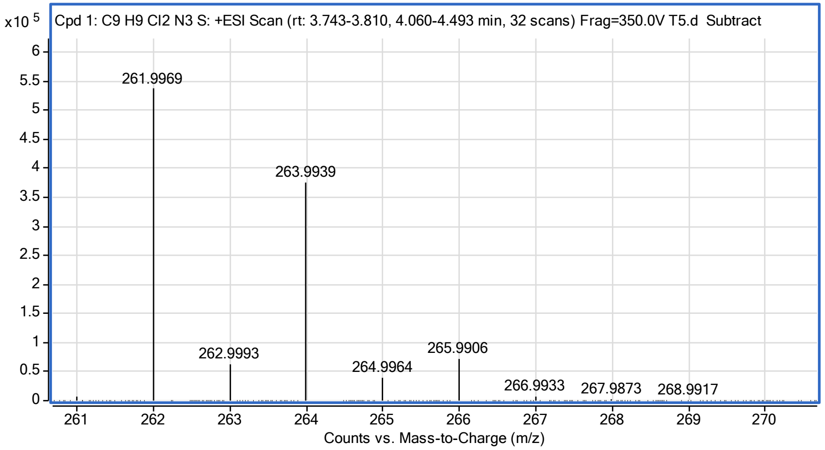
T6	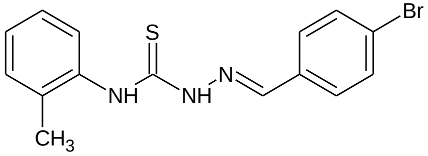<br>M=348,26	C15H14N3SBr<br>Calculated monoisotopic mass: 347.0092<br><br>Measured monoisotopic mass: 347.0096<br><br>Mass error: 1.14 ppm	Theoretical monoisotopic mass of $[M+H]^+$: 348.0165<br>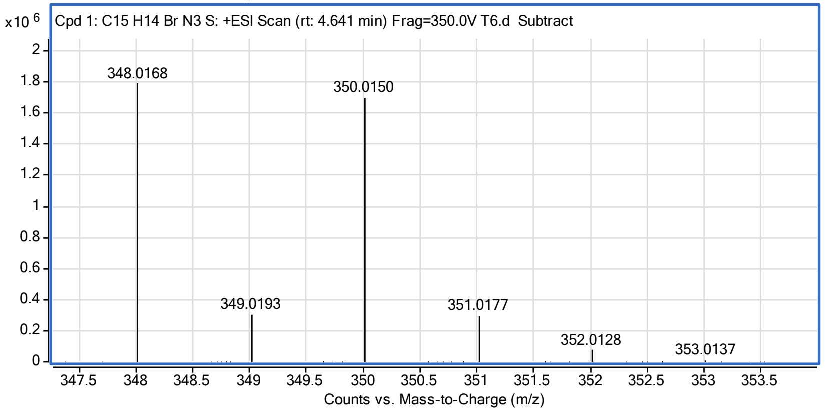

T7	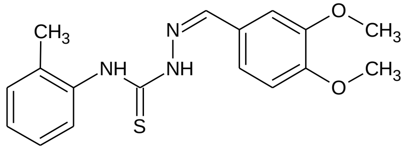<br>M=329,41	C17H19N3O2S<br>Calculated monoisotopic mass: 329.1198<br><br>Measured monoisotopic mass: 329.1202<br><br>Mass error: 1.31 ppm	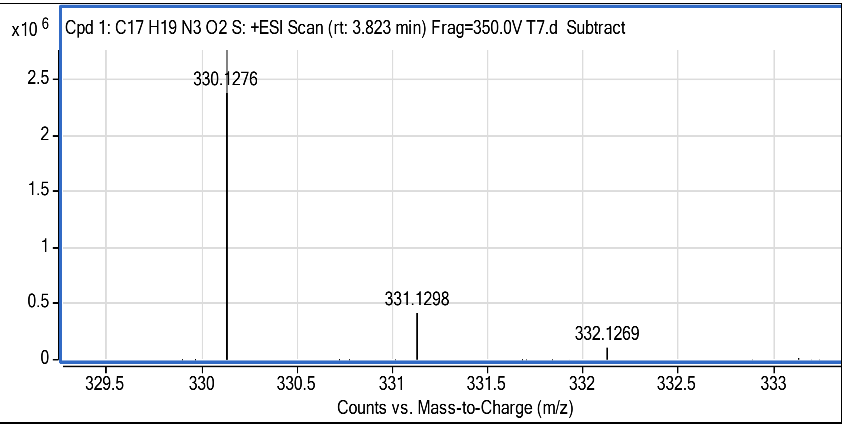
T8	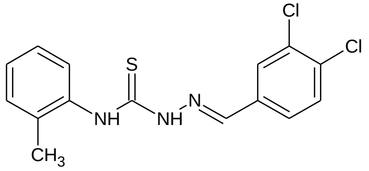<br>M=338,25	C15H13N3SCl2<br>Calculated monoisotopic mass: 337.0207<br><br>Measured monoisotopic mass: 337.0211<br><br>Mass error: 1.23 ppm	Theoretical monoisotopic mass of $[M+H]^+$: 338.0280<br>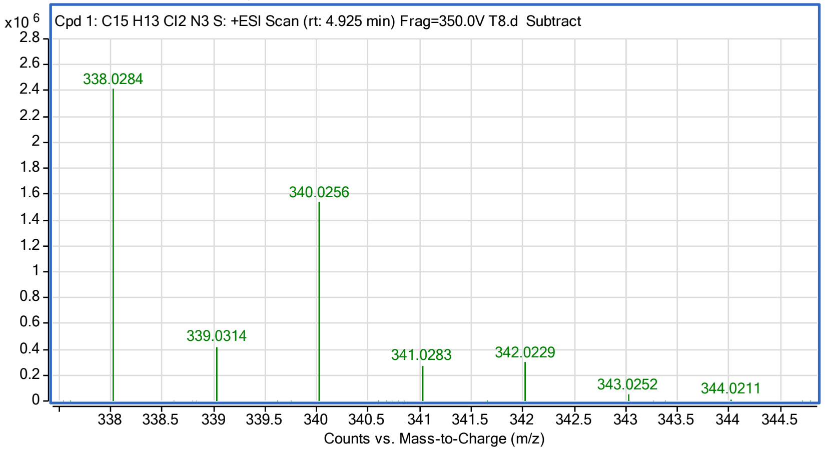
T9	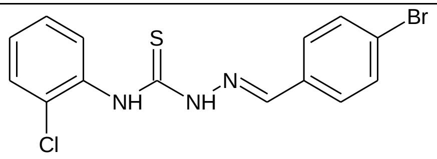<br>M=368,68	C14H11N3SClBr<br>Calculated monoisotopic mass: 366.9546<br><br>Measured monoisotopic mass: 366.9545<br><br>Mass error: -0.03 ppm	Theoretical monoisotopic mass of $[M+H]^+$: 367.9618

			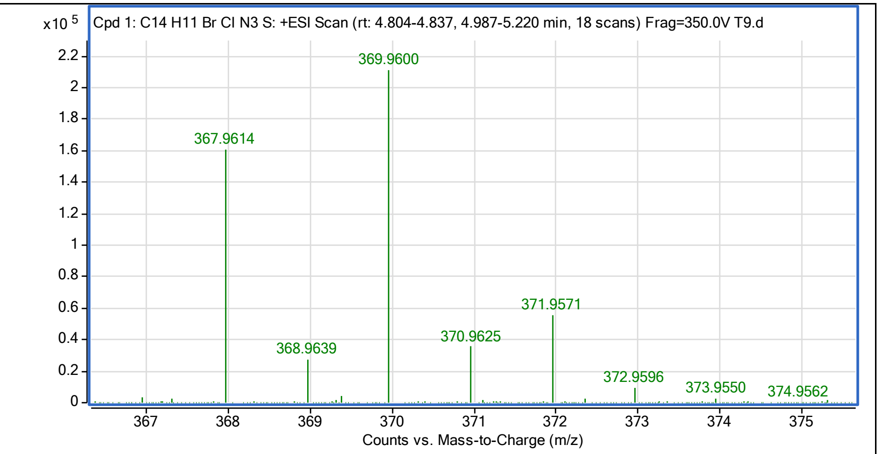
T10	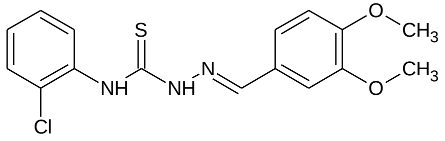<br>M=349,84	C16H16N3O2SCl<br>Calculated monoisotopic mass: 349.0652<br><br>Measured monoisotopic mass: 349.0653<br><br>Mass error: 0.35 ppm	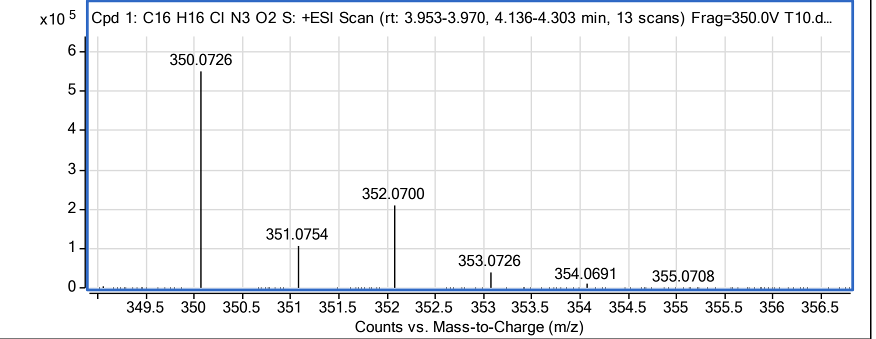
T11	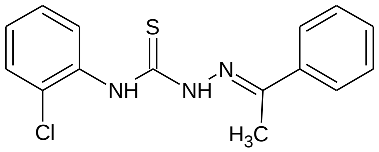<br>M=303,80	C15H14N3SCl<br>Calculated monoisotopic mass: 303.0597<br><br>Measured monoisotopic mass: 303.0601<br><br>Mass error: 1.31 ppm	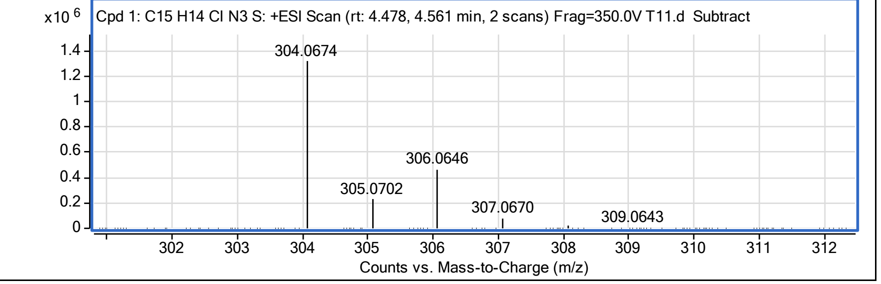

<table>
<tr>
<td>T12</td>
<td>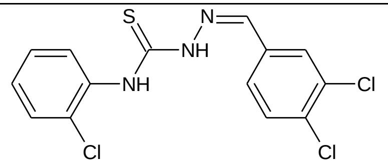

M=358,67</td>
<td>C14H10N3SCl3
Calculated monoisotopic mass: 356.9661

Measured monoisotopic mass: 356.9654

Mass error: -1.83 ppm</td>
<td>Theoretical monoisotopic mass of $[M+H]^+$: 357.9734
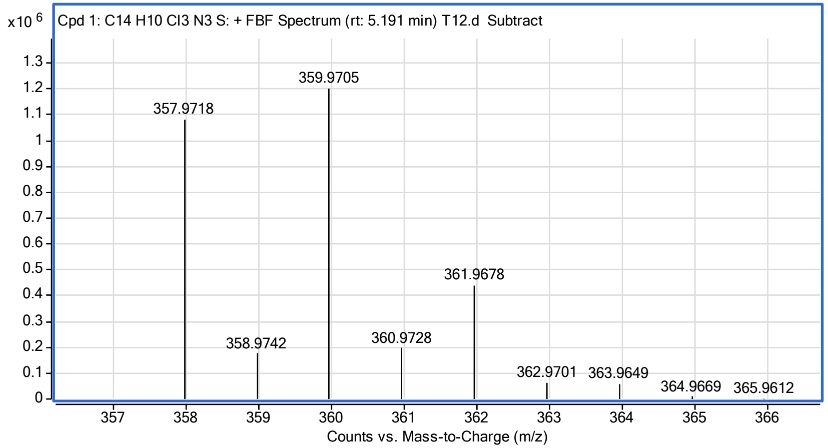
</td>
</tr>
<tr>
<td>T13</td>
<td>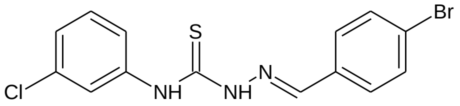

M=368,68</td>
<td>C14H11N3SClBr
Calculated monoisotopic mass: 366.9546

Measured monoisotopic mass: 366.9551

Mass error: 1.46 ppm</td>
<td>Theoretical monoisotopic mass of $[M+H]^+$: 367.9618
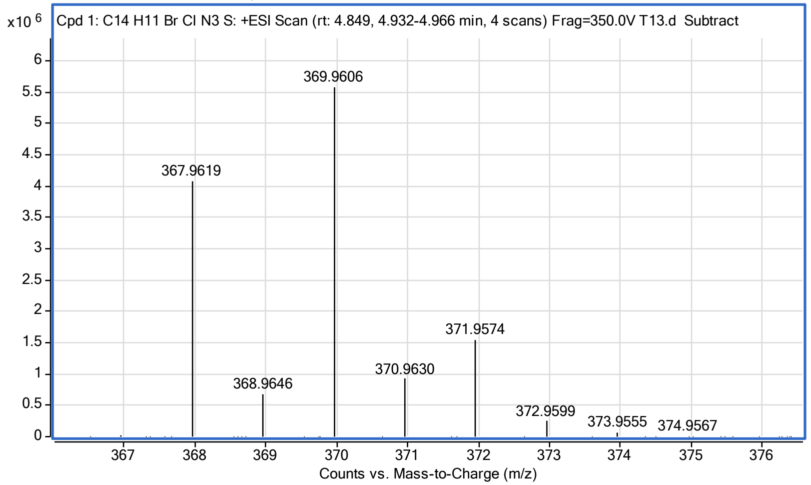
</td>
</tr>
</table>

T17	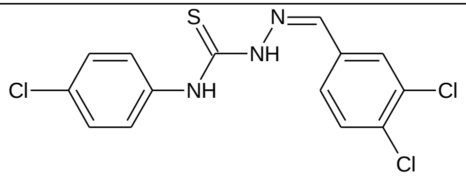<br><br>M=358,67	C14H10N3SCl3<br>Calculated monoisotopic mass: 356.9661<br><br>Measured monoisotopic mass: 356.9664<br><br>Mass error: 0.96 ppm	Theoretical monoisotopic mass of $[M+H]^+$: 357.9734<br>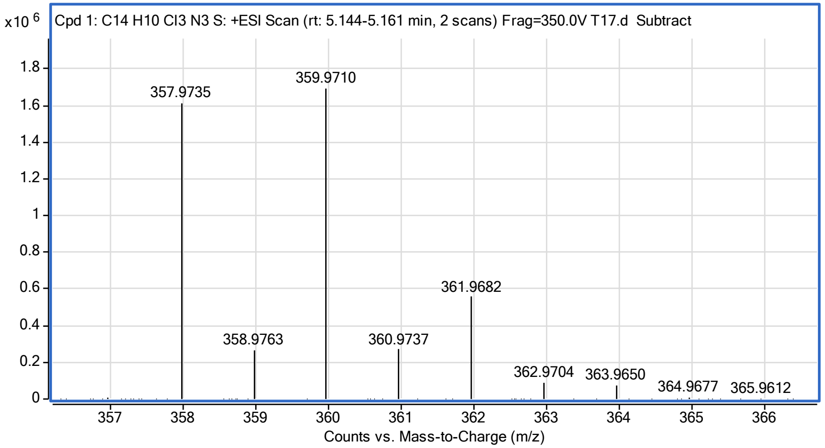

T1

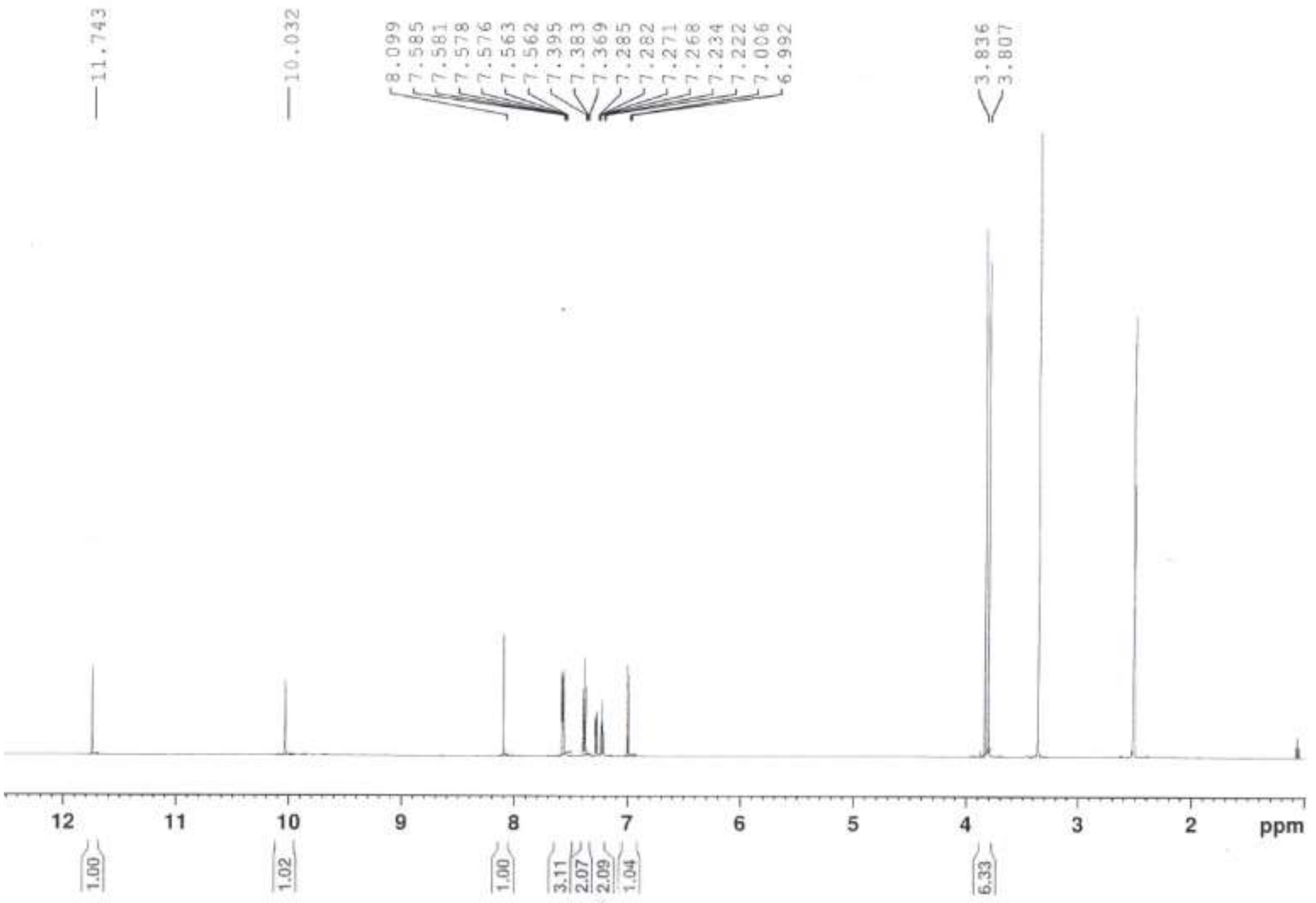

11.743
10.032
8.099
7.585
7.581
7.578
7.576
7.563
7.562
7.395
7.383
7.369
7.285
7.282
7.271
7.268
7.234
7.222
7.006
6.992
3.836
3.807
12
11
10
9
8
7
6
5
4
3
2
ppm
1.00
1.02
1.00
3.11
2.07
2.09
1.04
6.33

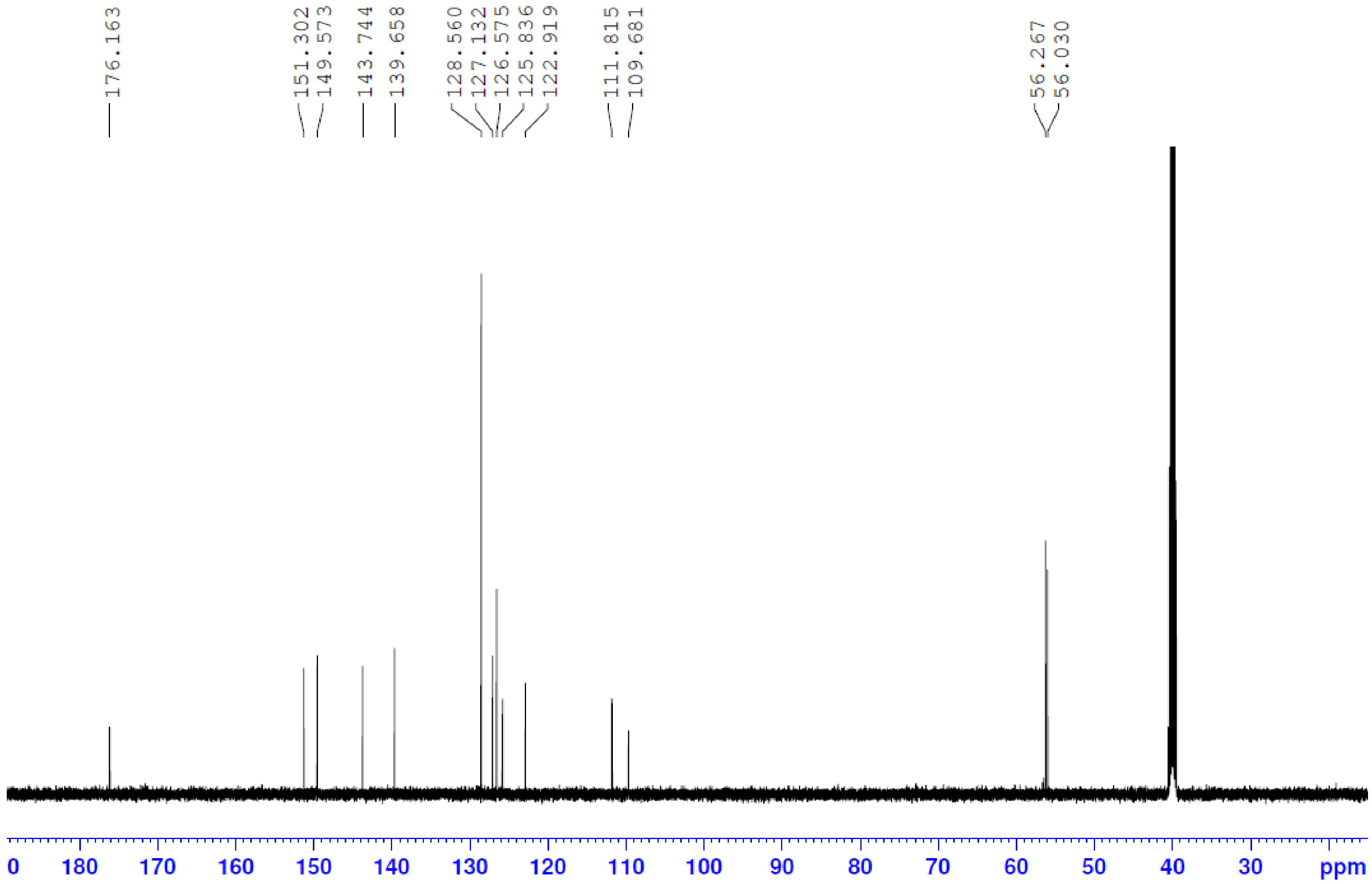
176.163
151.302
149.573
143.744
139.658
128.560
127.132
126.575
125.836
122.919
111.815
109.681
56.267
56.030
0
180
170
160
150
140
130
120
110
100
90
80
70
60
50
40
30
ppm

T2

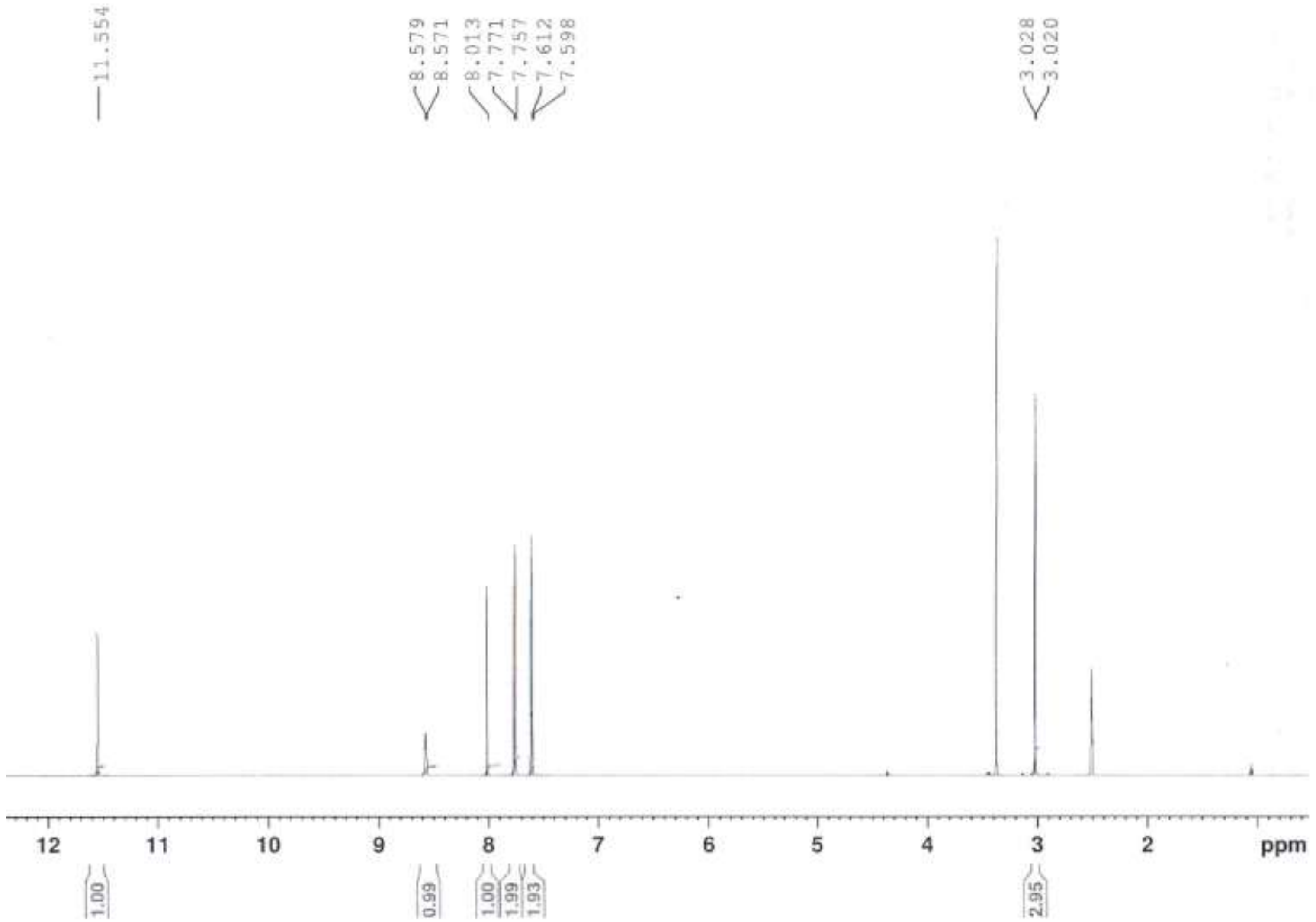
11.554
8.579
8.571
8.013
7.771
7.757
7.612
7.598
3.028
3.020
12
11
10
9
8
7
6
5
4
3
2
ppm
1.00
0.99
1.00
1.99
1.93
2.95

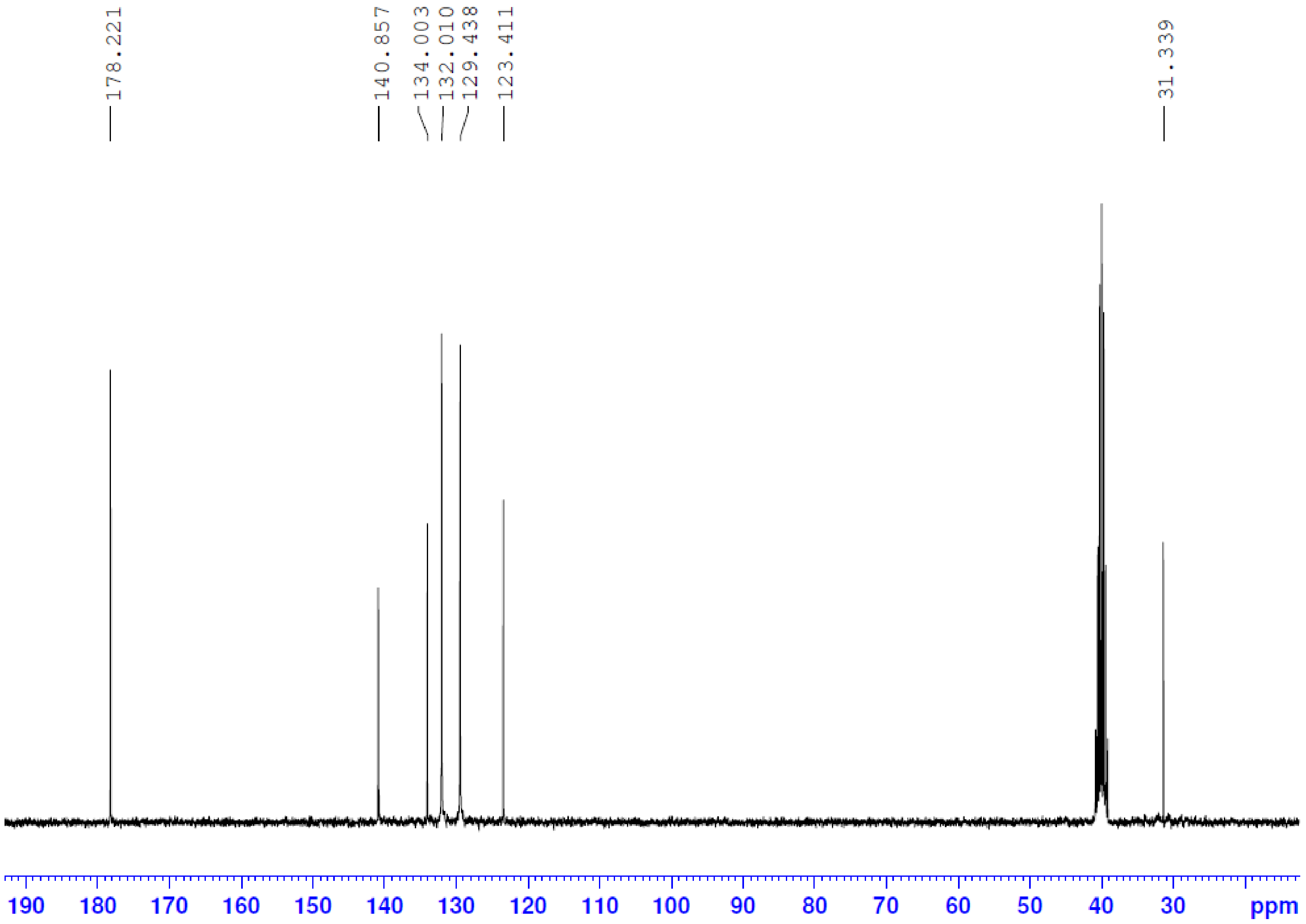
178.221
140.857
134.003
132.010
129.438
123.411
31.339
190
180
170
160
150
140
130
120
110
100
90
80
70
60
50
40
30
ppm

T3

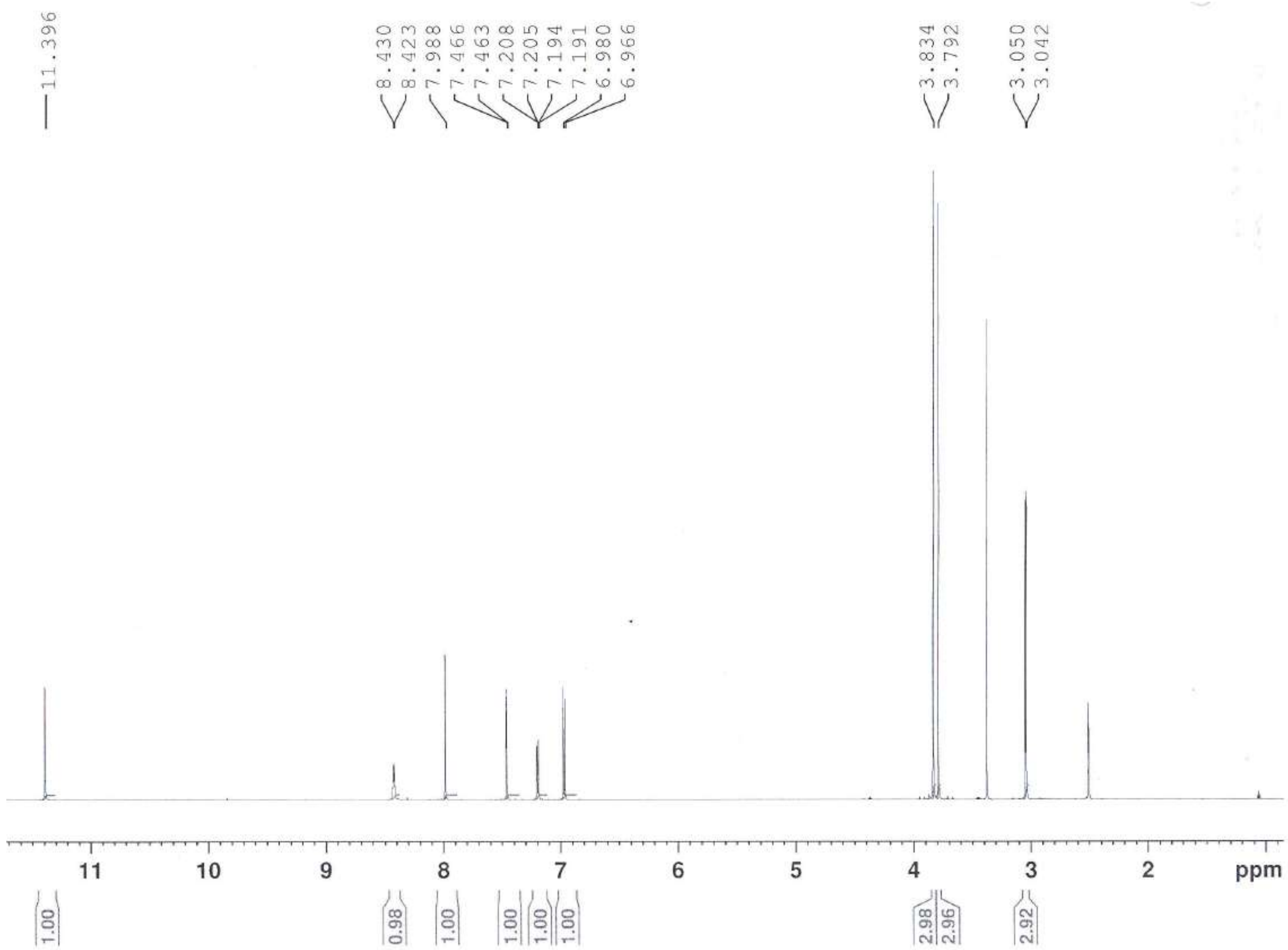
11.396
8.430
8.423
7.988
7.466
7.463
7.208
7.205
7.194
7.191
6.980
6.966
3.834
3.792
3.050
3.042
11
10
9
8
7
6
5
4
3
2
ppm
1.00
0.98
1.00
1.00
1.00
1.00
2.98
2.96
2.92

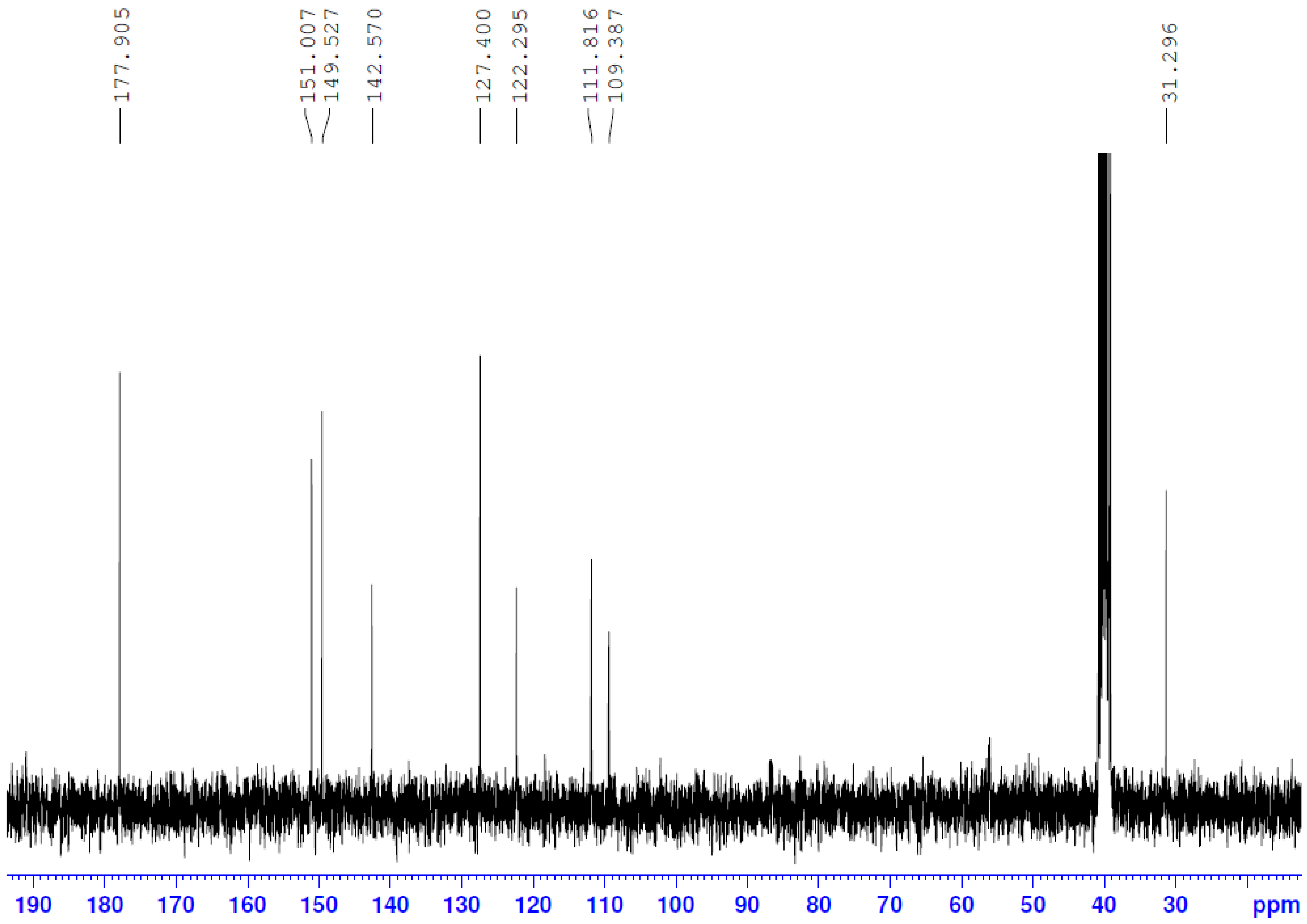

177.905
151.007
149.527
142.570
127.400
122.295
111.816
109.387
31.296
190
180
170
160
150
140
130
120
110
100
90
80
70
60
50
40
30
ppm

T4

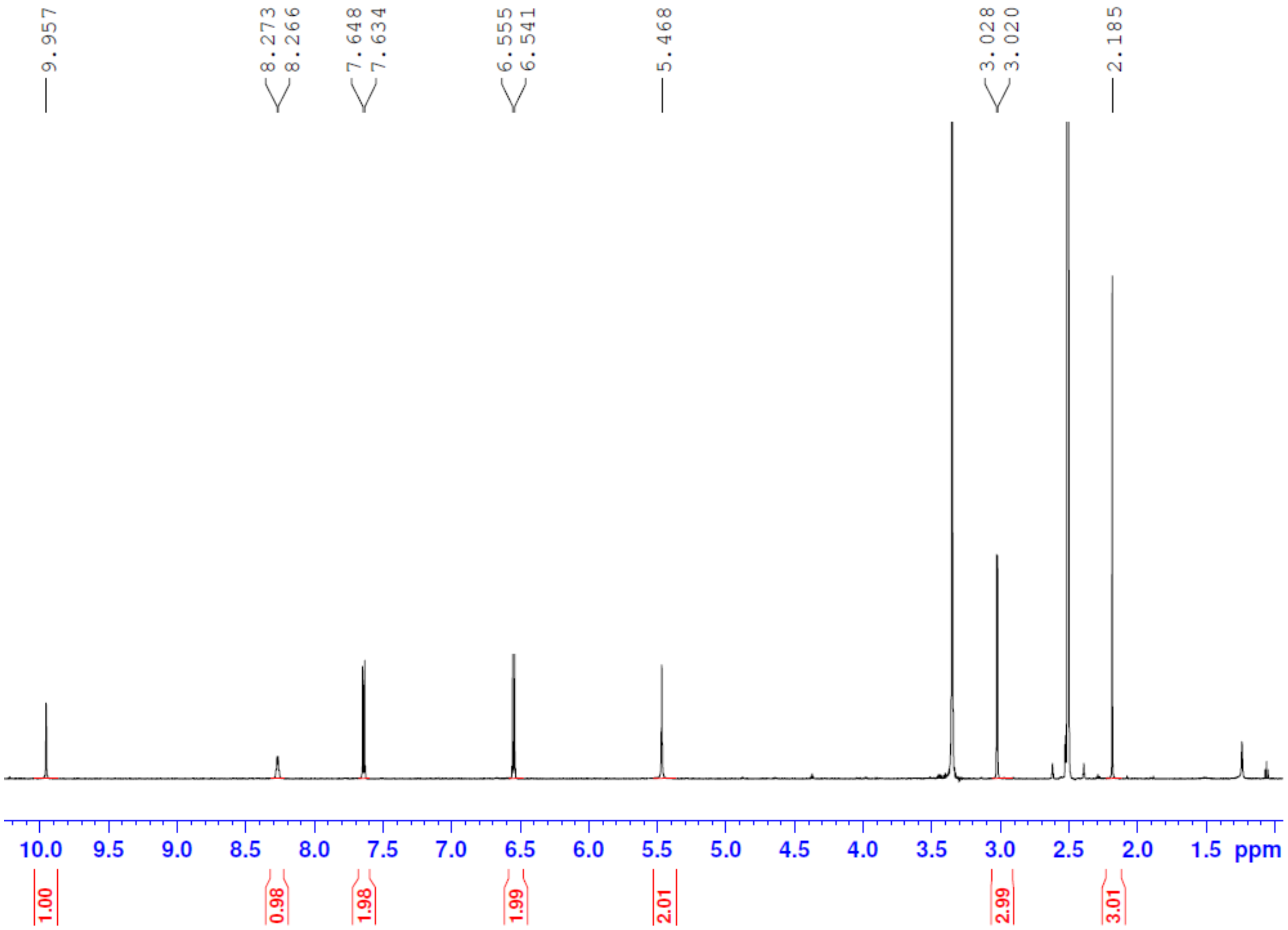
9.957
8.273
8.266
7.648
7.634
6.555
6.541
5.468
3.028
3.020
2.185
10.0 9.5 9.0 8.5 8.0 7.5 7.0 6.5 6.0 5.5 5.0 4.5 4.0 3.5 3.0 2.5 2.0 1.5 ppm
1.00
0.98
1.98
1.99
2.01
2.99
3.01

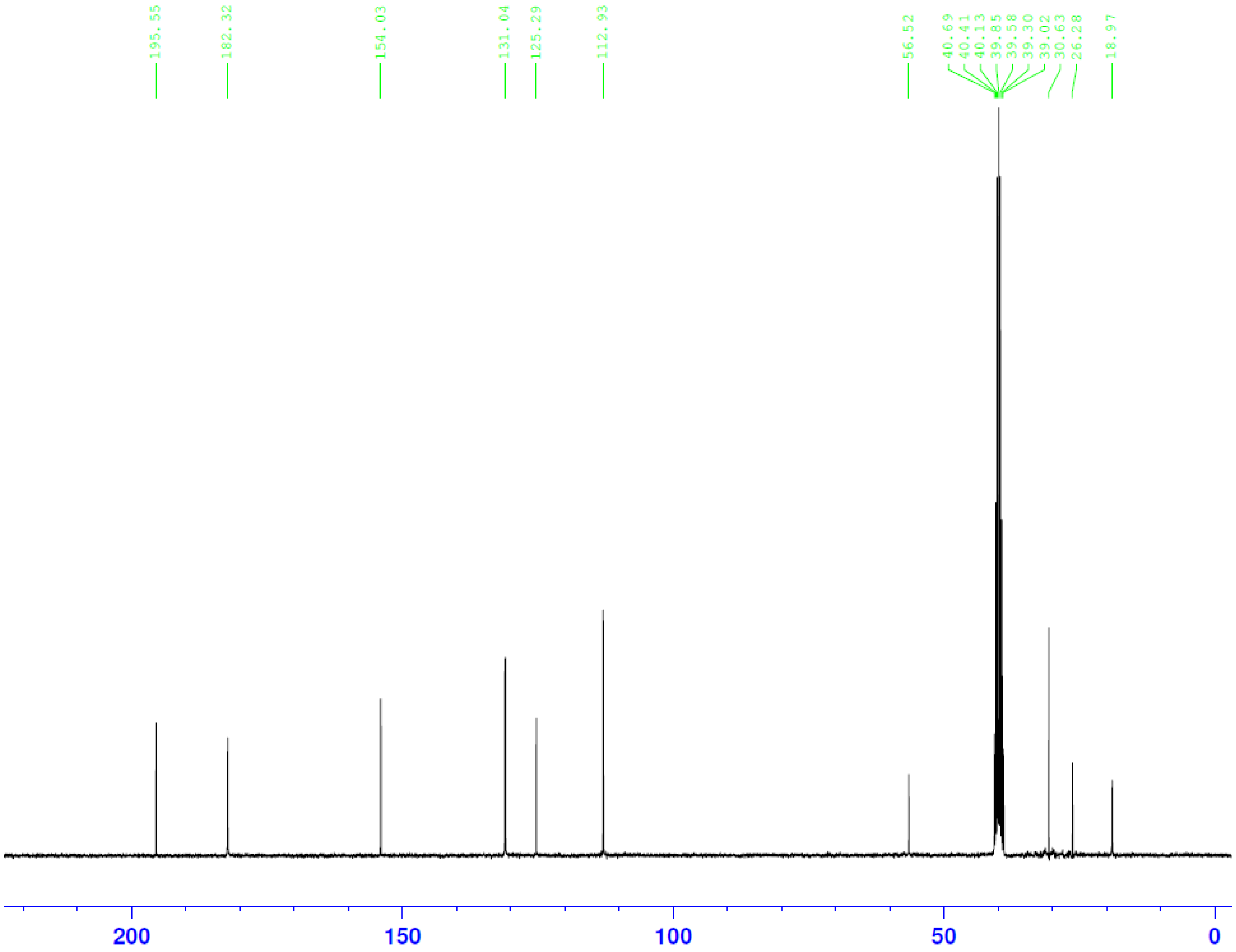
195.55
182.32
154.03
131.04
125.29
112.93
56.52
40.69
40.41
40.13
39.85
39.58
39.30
39.02
30.63
26.28
18.97
200
150
100
50
0

T5

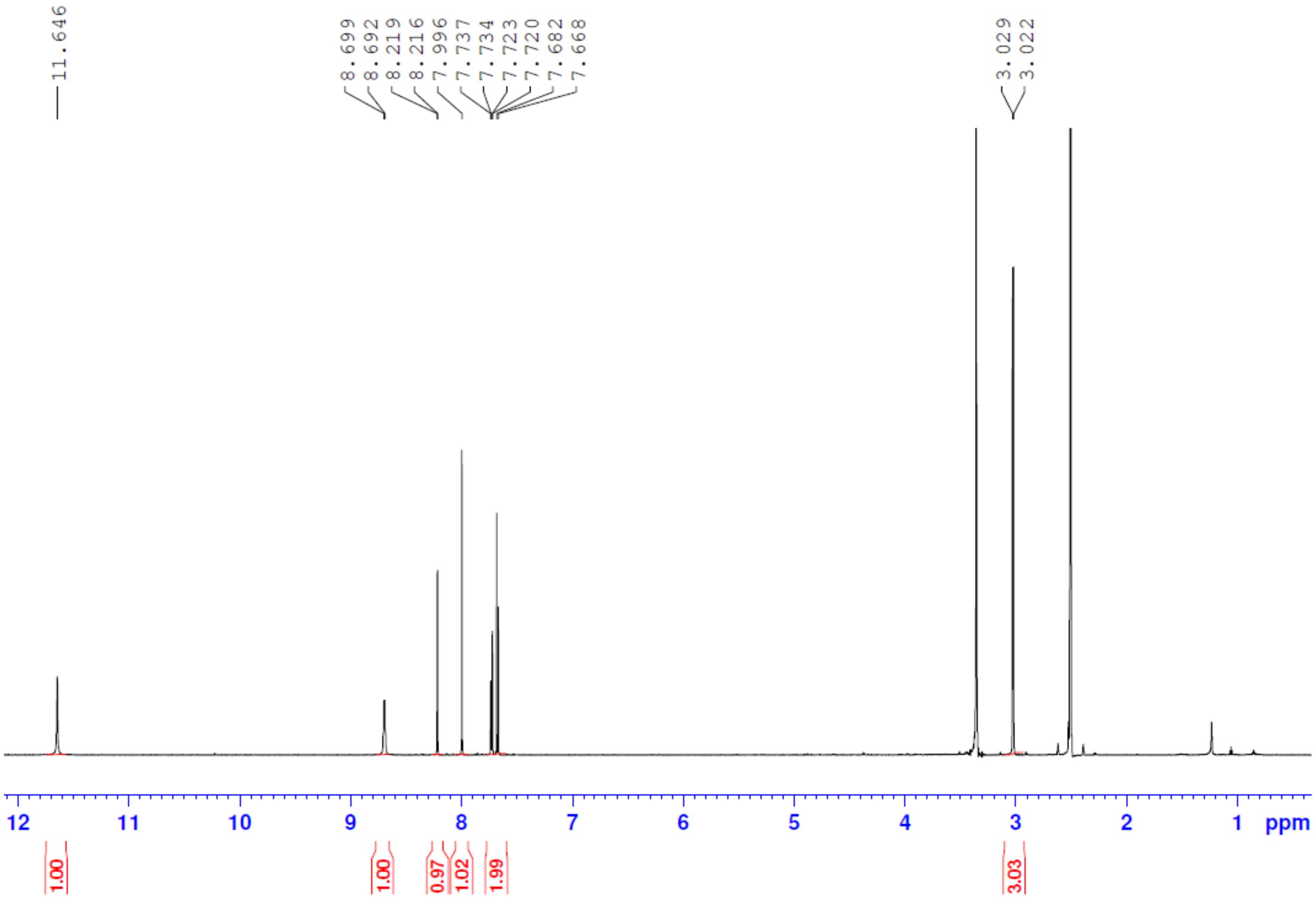
11.646
8.699
8.692
8.219
8.216
7.996
7.737
7.734
7.723
7.720
7.682
7.668
3.029
3.022
12 11 10 9 8 7 6 5 4 3 2 1 ppm
1.00
1.00
0.97
1.02
1.99
3.03

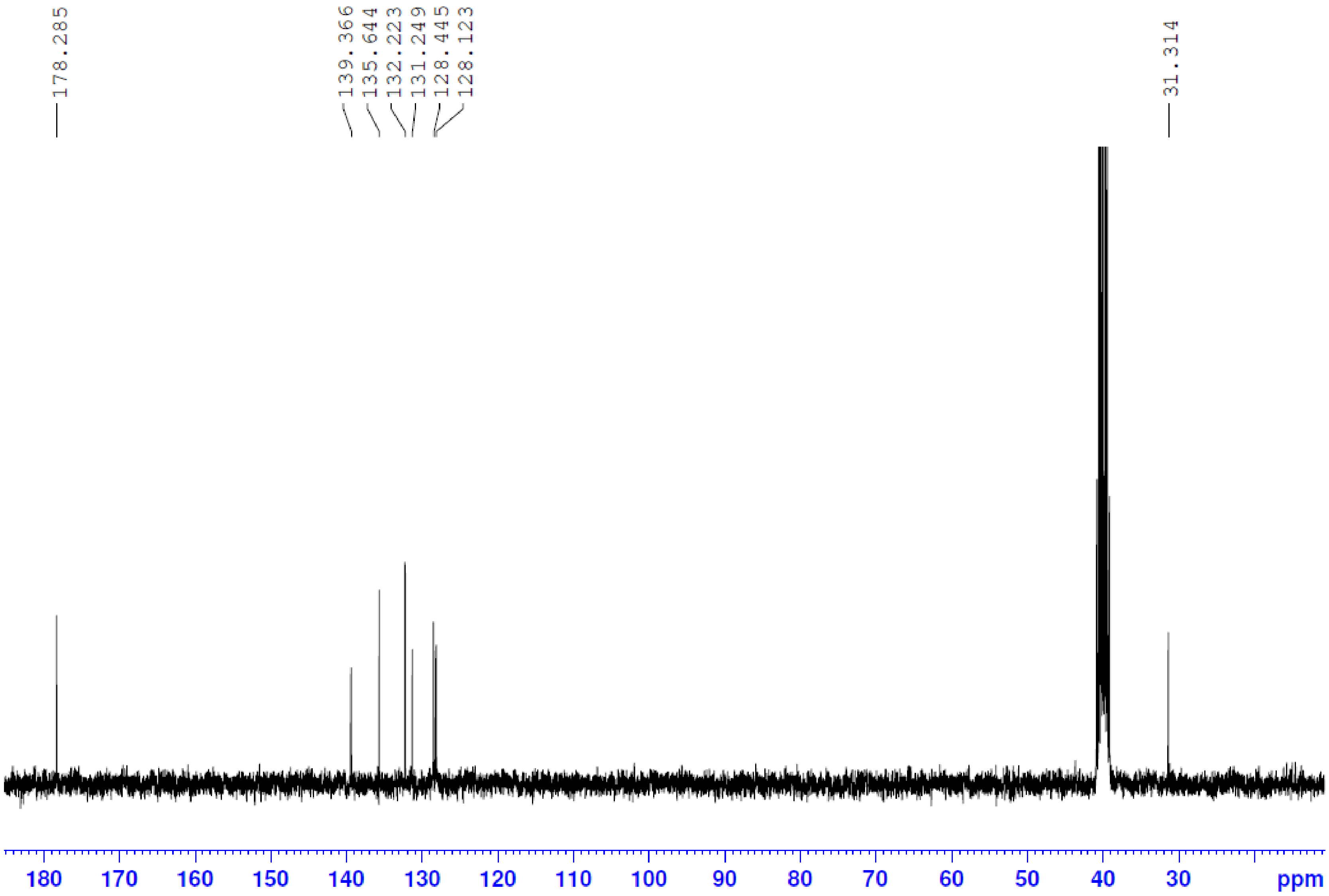
178.285
139.366
135.644
132.223
131.249
128.445
128.123
31.314
180 170 160 150 140 130 120 110 100 90 80 70 60 50 40 30 ppm

T6

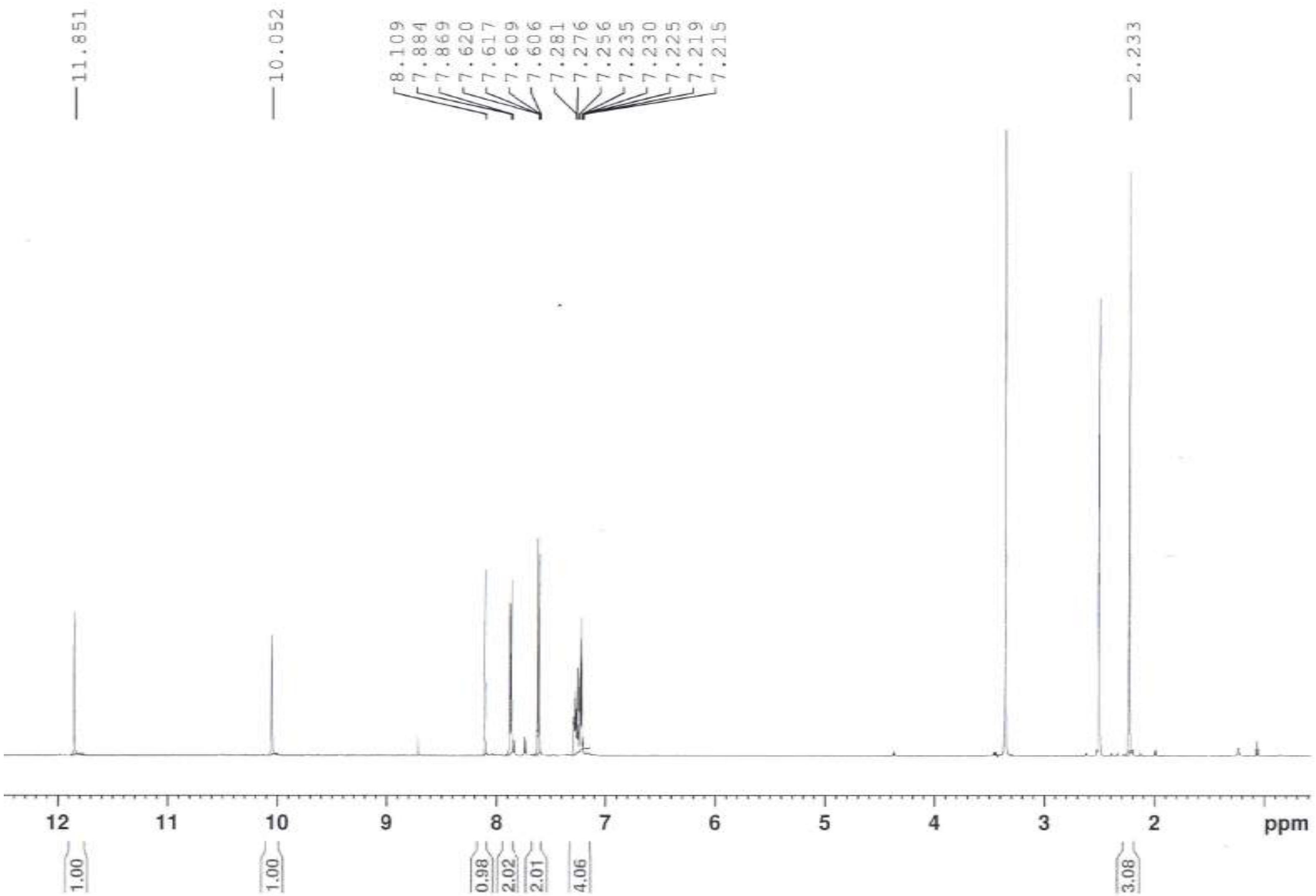
11.851
10.052
8.109
7.884
7.869
7.620
7.617
7.609
7.606
7.281
7.276
7.256
7.235
7.230
7.225
7.219
7.215
2.233
12
11
10
9
8
7
6
5
4
3
2
ppm
1.00
1.00
0.98
2.02
2.01
4.06
3.08

T7

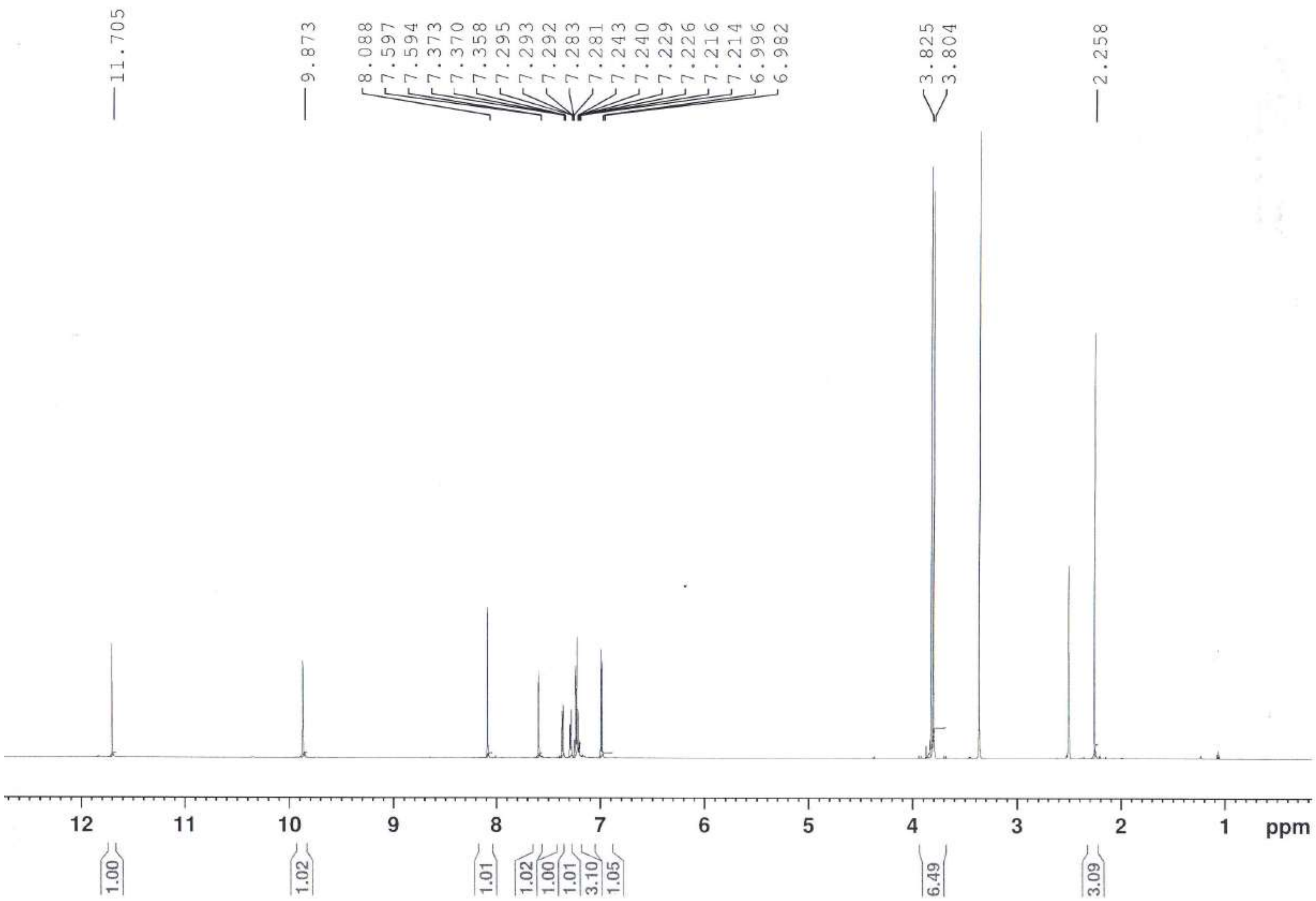

11.705
9.873
8.088
7.597
7.594
7.373
7.370
7.358
7.295
7.293
7.292
7.283
7.281
7.243
7.240
7.229
7.226
7.216
7.214
6.996
6.982
3.825
3.804
2.258
12
11
10
9
8
7
6
5
4
3
2
1
ppm
1.00
1.02
1.01
1.02
1.00
1.01
3.10
1.05
6.49
3.09

T8

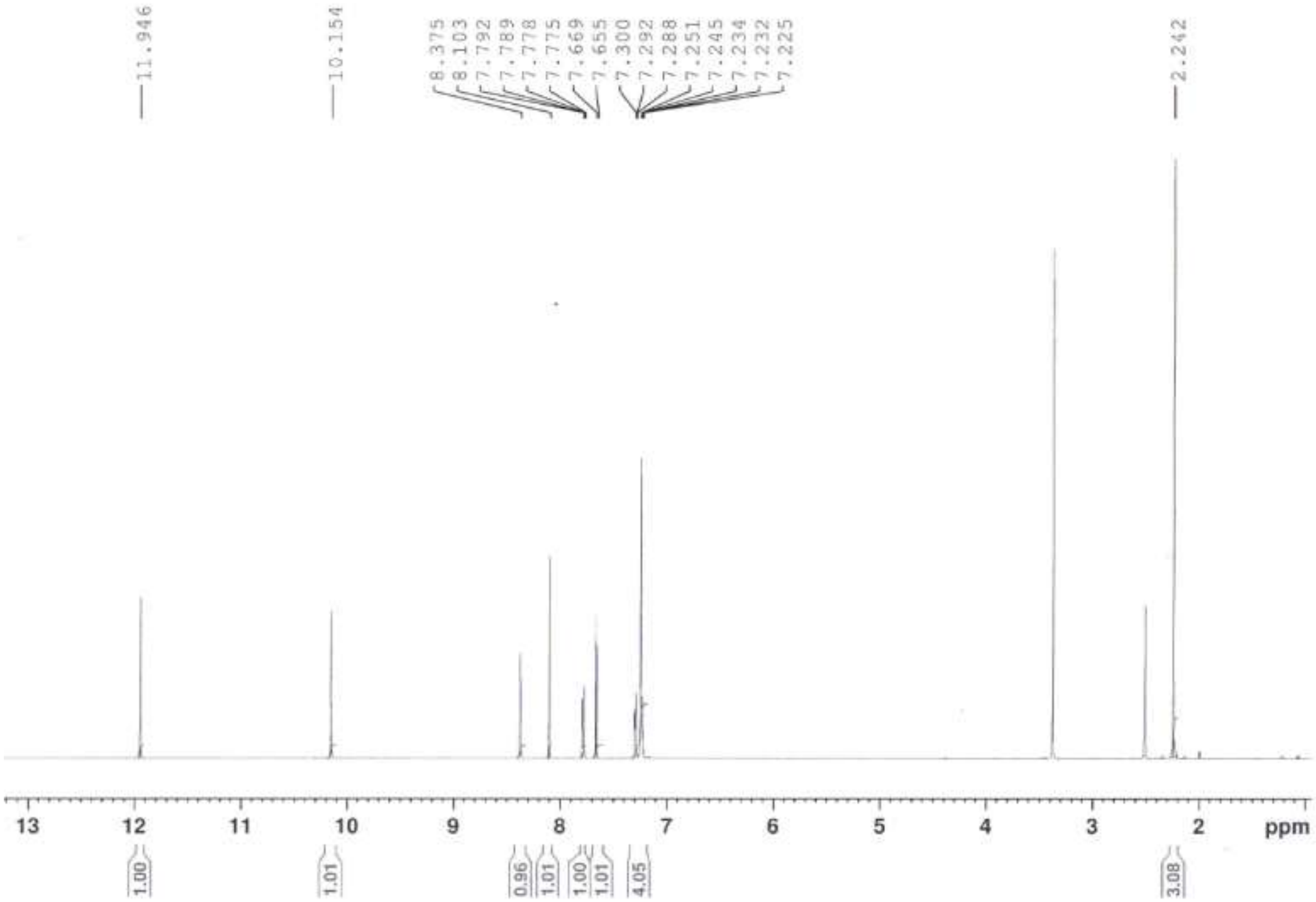
11.946
10.154
8.375
8.103
7.792
7.789
7.778
7.775
7.669
7.655
7.300
7.292
7.288
7.251
7.245
7.234
7.232
7.225
2.242
13
12
11
10
9
8
7
6
5
4
3
2
ppm
1.00
1.01
0.96
1.01
1.00
1.01
4.05
3.08

T9

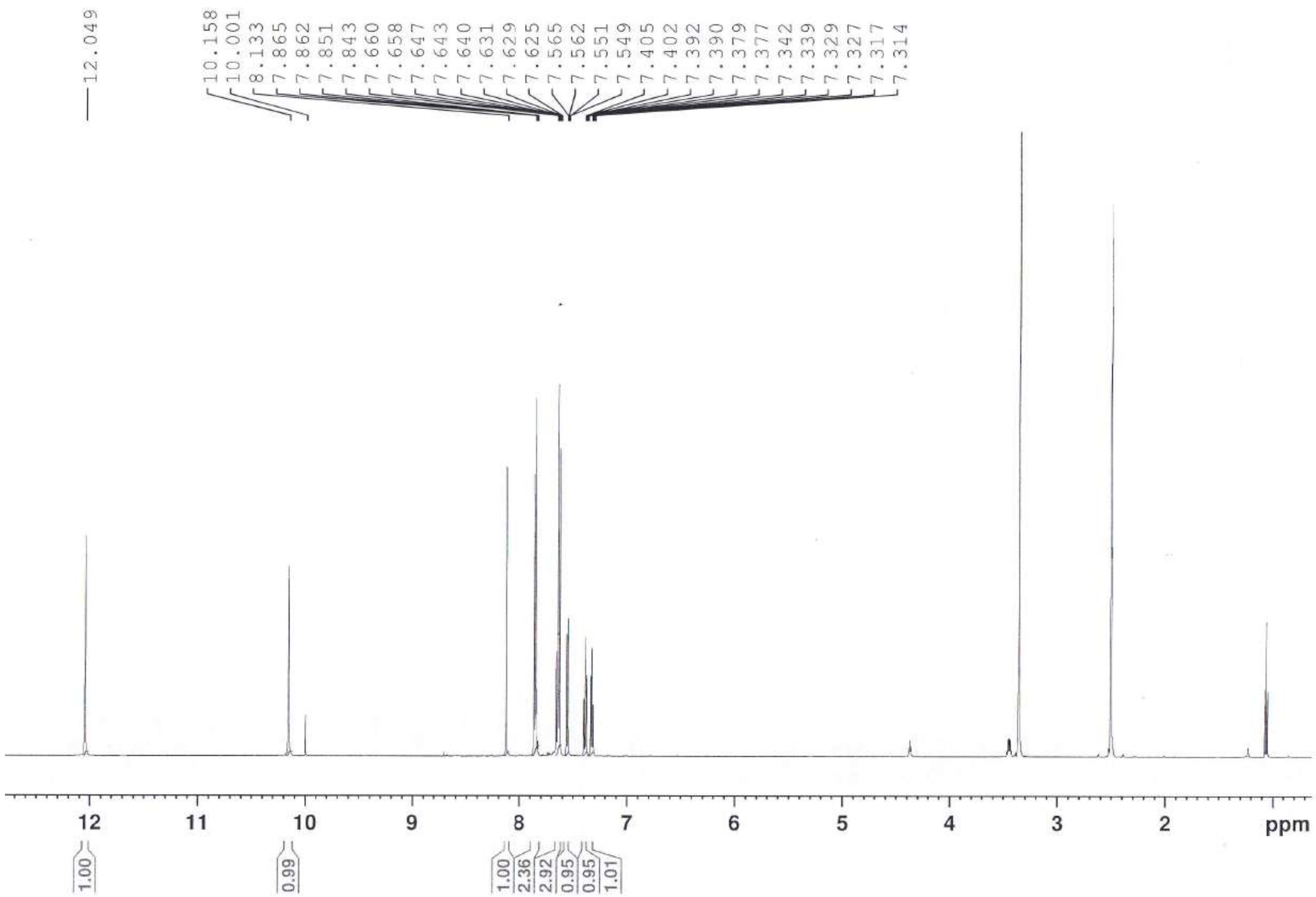
12.049
10.158
10.001
8.133
7.865
7.862
7.851
7.843
7.660
7.658
7.647
7.643
7.640
7.631
7.629
7.625
7.565
7.562
7.551
7.549
7.405
7.402
7.392
7.390
7.379
7.377
7.342
7.339
7.329
7.327
7.317
7.314
12
11
10
9
8
7
6
5
4
3
2
ppm
1.00
0.99
1.00
2.36
2.92
0.95
0.95
1.01

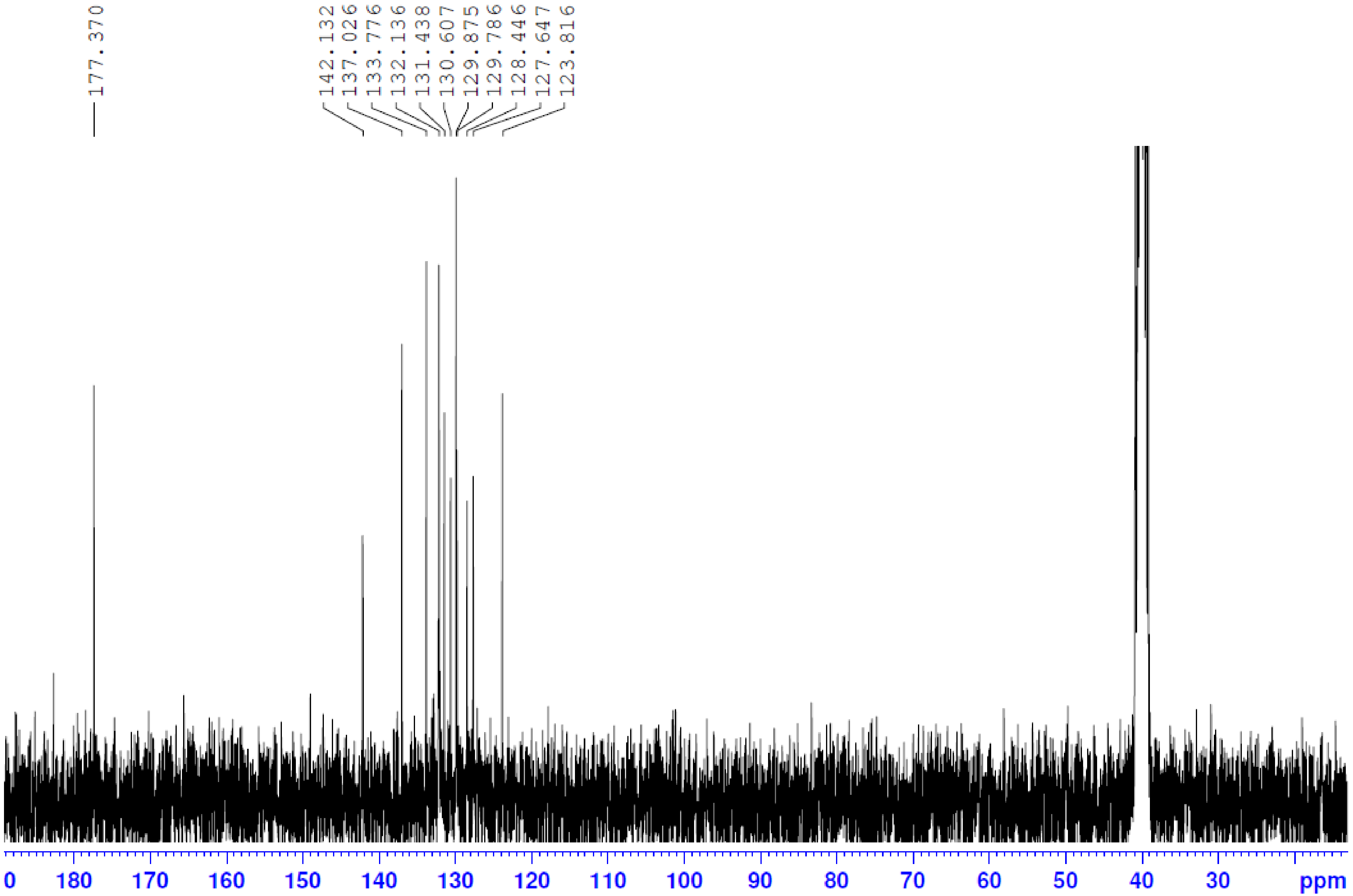
177.370
142.132
137.026
133.776
132.136
131.438
130.607
129.875
129.786
128.446
127.647
123.816
0
180
170
160
150
140
130
120
110
100
90
80
70
60
50
40
30
ppm

T10

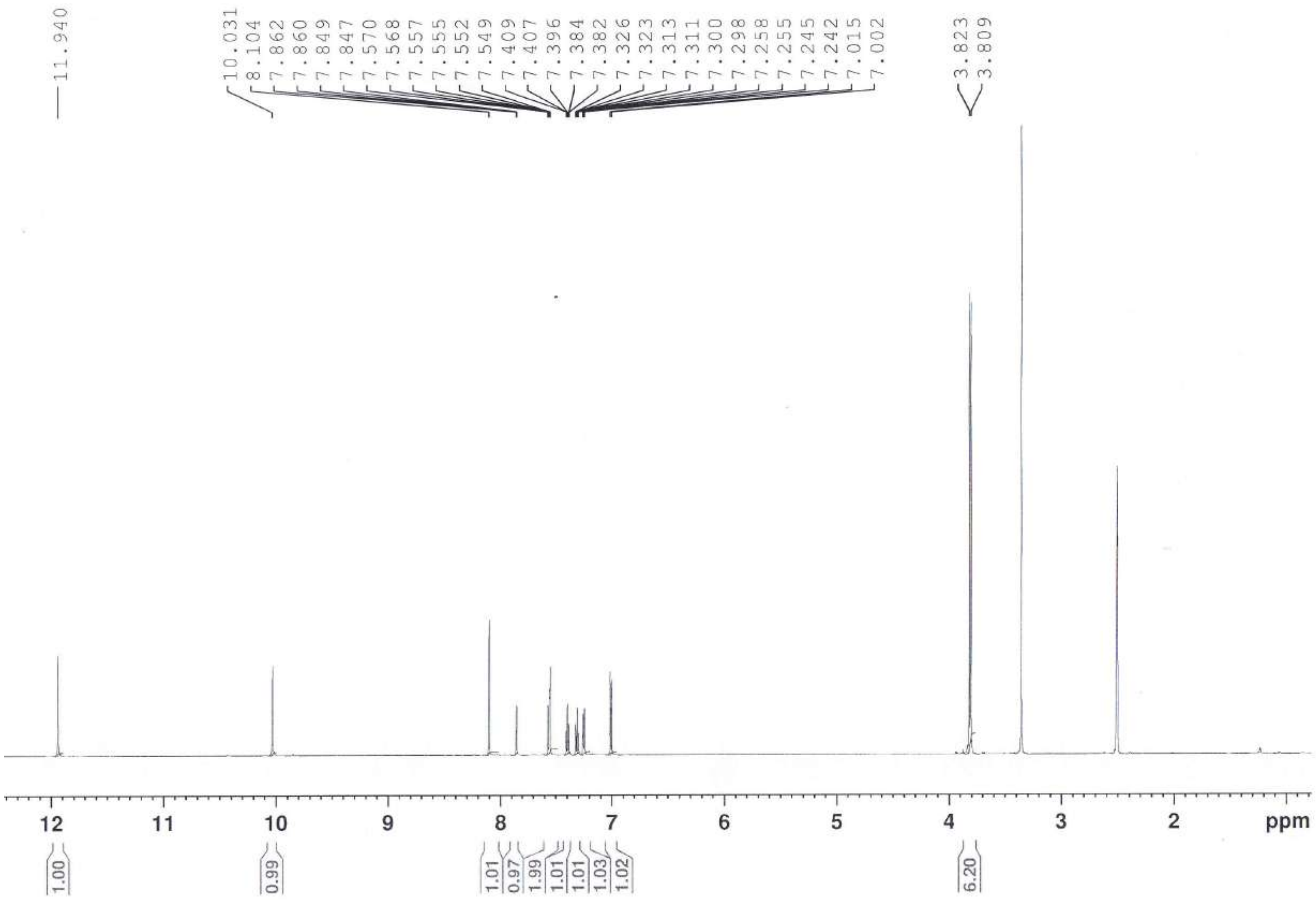
11.940
10.031
8.104
7.862
7.860
7.849
7.847
7.570
7.568
7.557
7.555
7.552
7.549
7.409
7.407
7.396
7.384
7.382
7.326
7.323
7.313
7.311
7.300
7.298
7.258
7.255
7.245
7.242
7.015
7.002
3.823
3.809
12
11
10
9
8
7
6
5
4
3
2
ppm
1.00
0.99
1.01
0.97
1.99
1.01
1.01
1.03
1.02
6.20

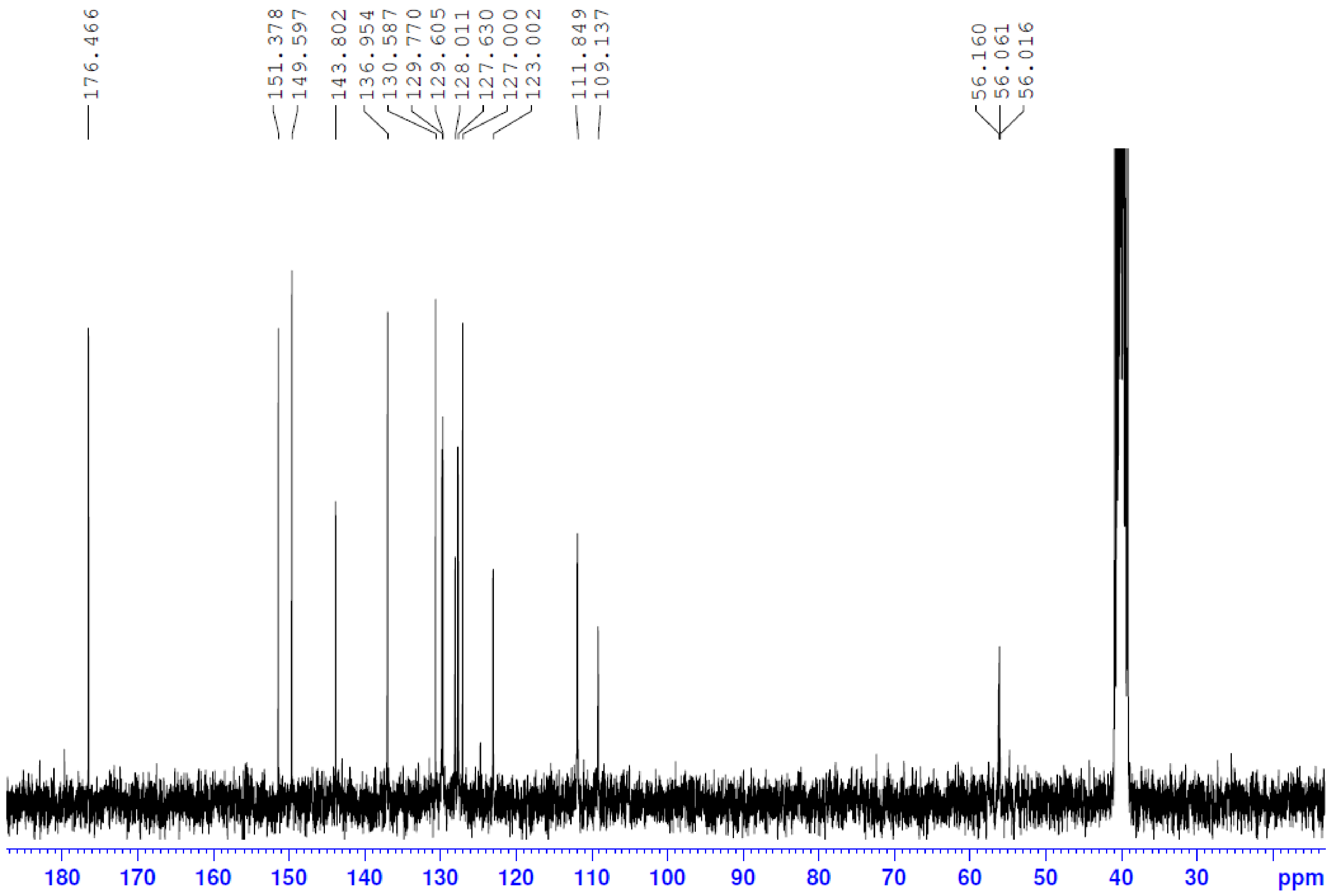
176.466
151.378
149.597
143.802
136.954
130.587
129.770
129.605
128.011
127.630
127.000
123.002
111.849
109.137
56.160
56.061
56.016
180 170 160 150 140 130 120 110 100 90 80 70 60 50 40 30 ppm

T11

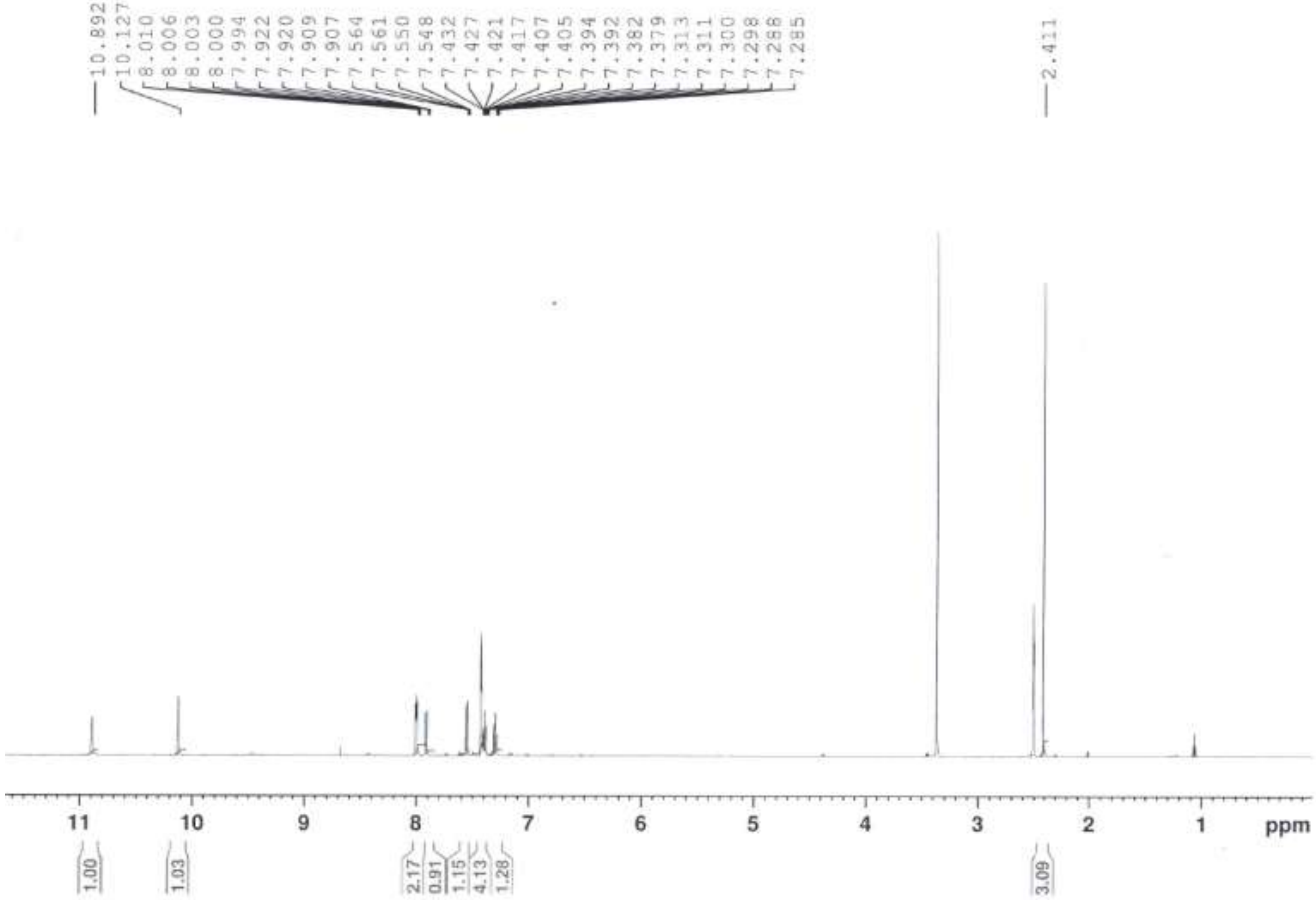
10.892
10.127
8.010
8.006
8.003
8.000
7.994
7.922
7.920
7.909
7.907
7.564
7.561
7.550
7.548
7.432
7.427
7.421
7.417
7.407
7.405
7.394
7.392
7.382
7.379
7.313
7.311
7.300
7.298
7.288
7.285
2.411
11 10 9 8 7 6 5 4 3 2 1 ppm
1.00
1.03
2.17
0.91
1.15
4.13
1.28
3.09

T12

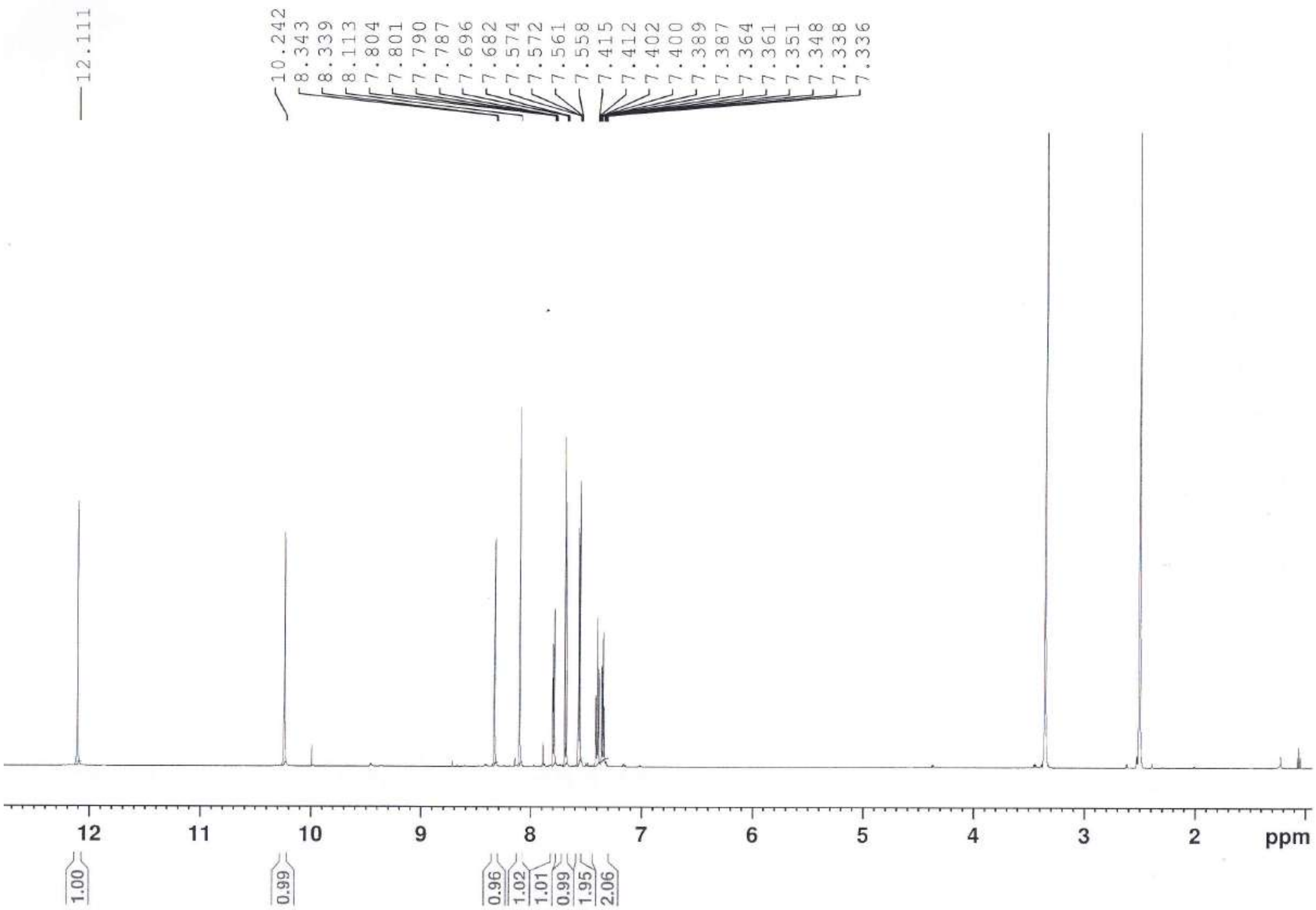

12.111
10.242
8.343
8.339
8.113
7.804
7.801
7.790
7.787
7.696
7.682
7.574
7.572
7.561
7.558
7.415
7.412
7.402
7.400
7.389
7.387
7.364
7.361
7.351
7.348
7.338
7.336
12
11
10
9
8
7
6
5
4
3
2
ppm
1.00
0.99
0.96
1.02
1.01
0.99
1.95
2.06

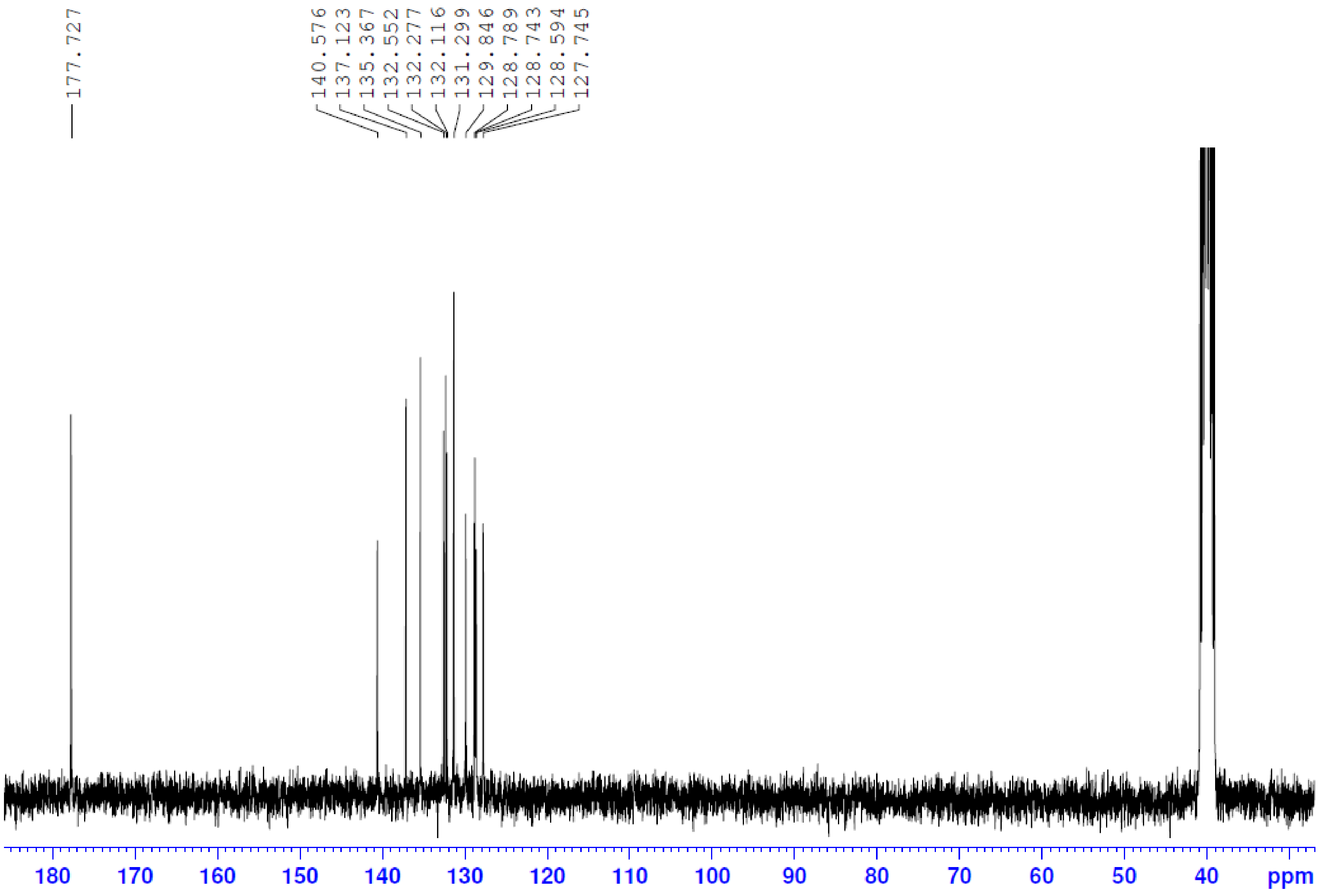
177.727
140.576
137.123
135.367
132.552
132.277
132.116
131.299
129.846
128.789
128.743
128.594
127.745
180
170
160
150
140
130
120
110
100
90
80
70
60
50
40
ppm

T13

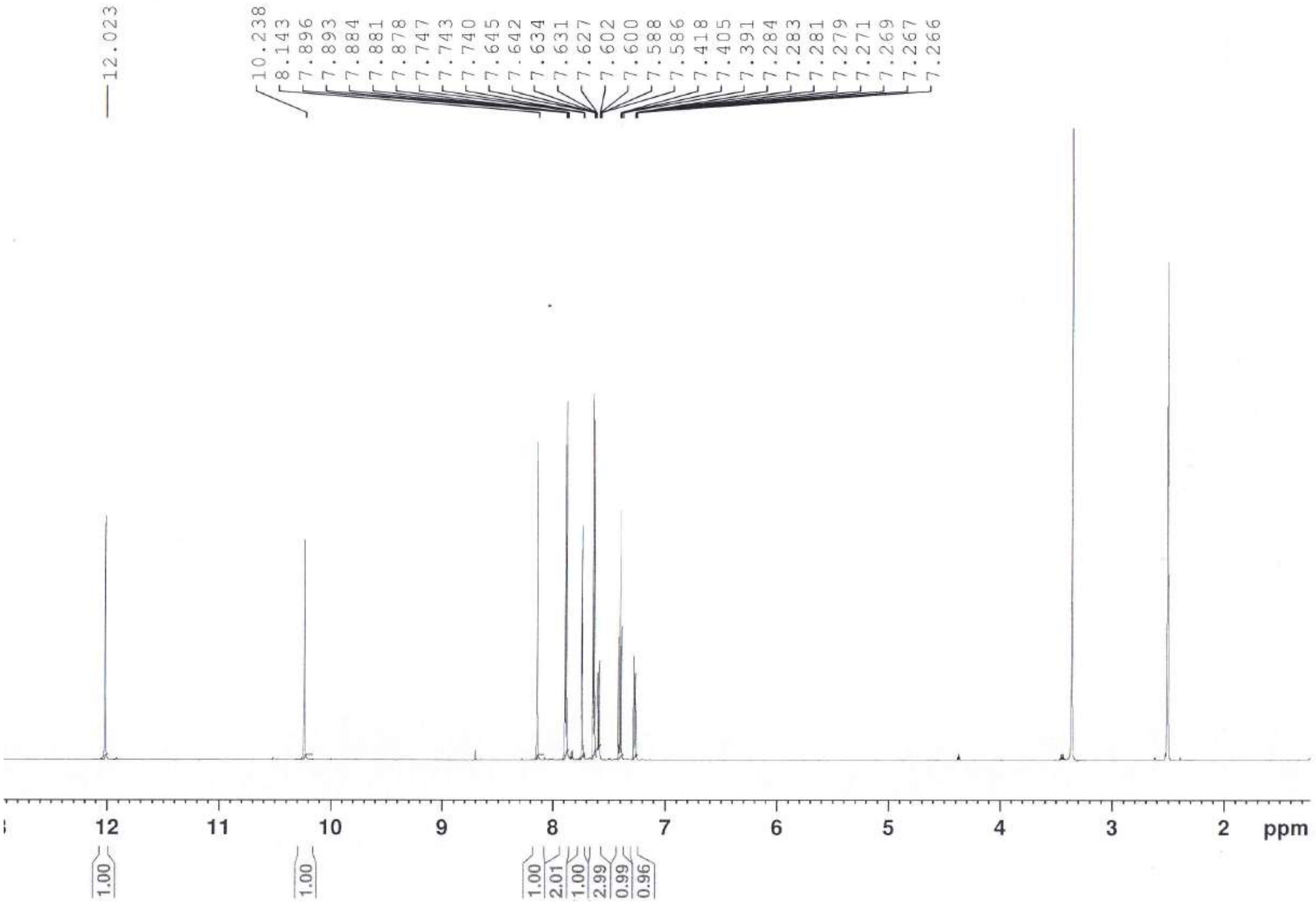
12.023
10.238
8.143
7.896
7.893
7.884
7.881
7.878
7.747
7.743
7.740
7.645
7.642
7.634
7.631
7.627
7.602
7.600
7.588
7.586
7.418
7.405
7.391
7.284
7.283
7.281
7.279
7.271
7.269
7.267
7.266
12
11
10
9
8
7
6
5
4
3
2
ppm
1.00
1.00
1.00
2.01
1.00
2.99
0.99
0.96

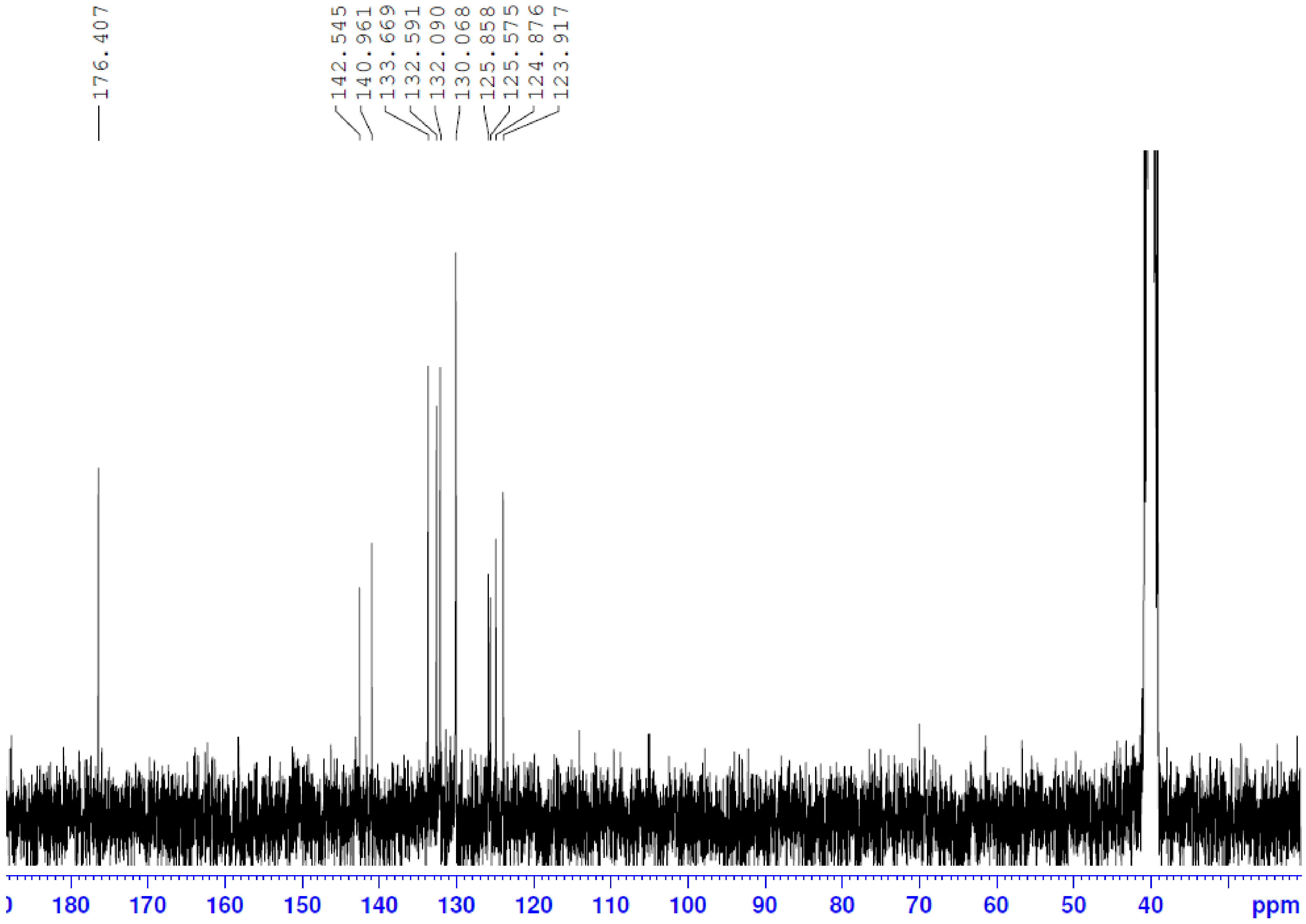
176.407
142.545
140.961
133.669
132.591
132.090
130.068
125.858
125.575
124.876
123.917
180
170
160
150
140
130
120
110
100
90
80
70
60
50
40
ppm

T14

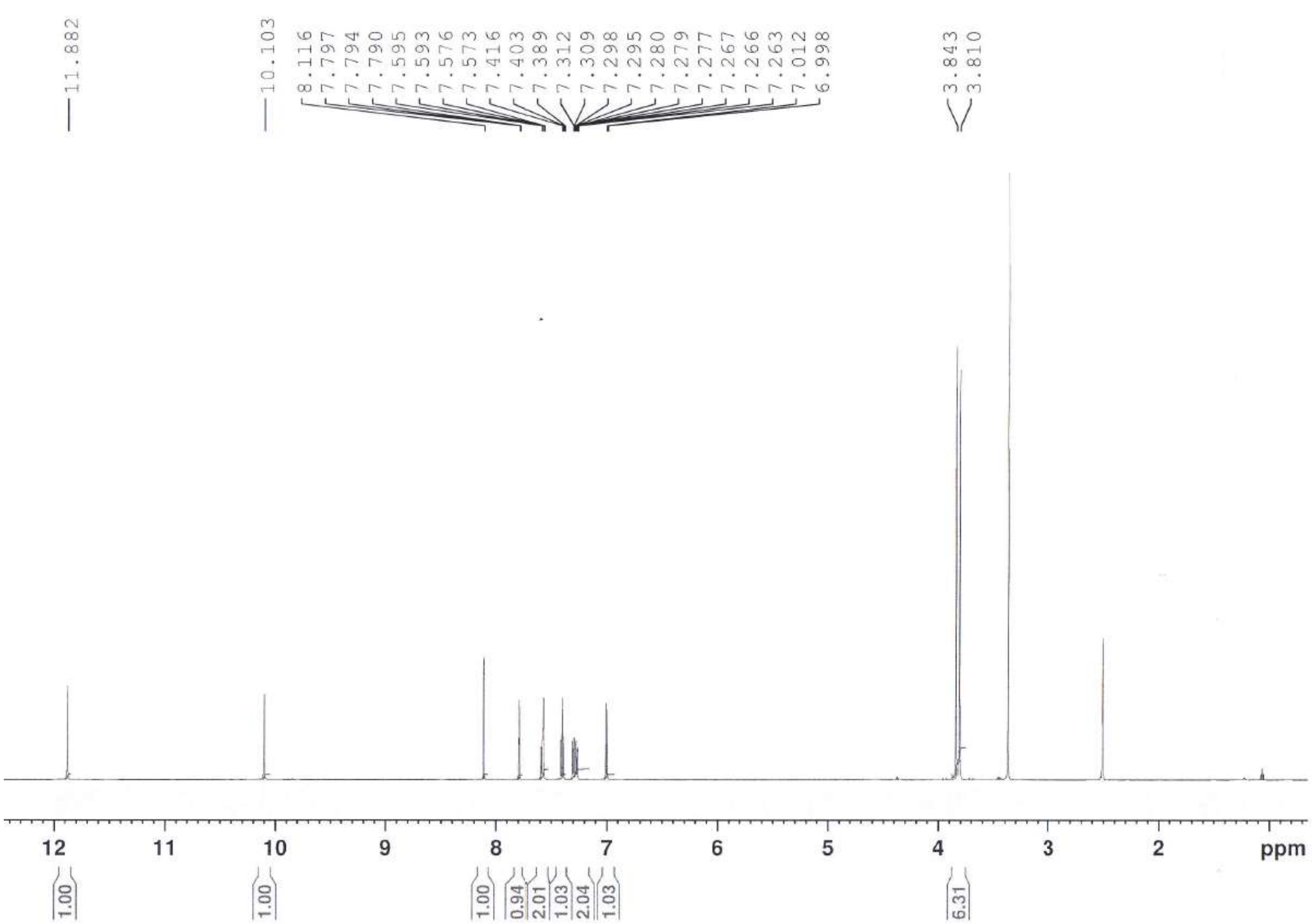

11.882
10.103
8.116
7.797
7.794
7.790
7.595
7.593
7.576
7.573
7.416
7.403
7.389
7.312
7.309
7.298
7.295
7.280
7.279
7.277
7.267
7.266
7.263
7.012
6.998
3.843
3.810
12
11
10
9
8
7
6
5
4
3
2
ppm
1.00
1.00
1.00
0.94
2.01
1.03
2.04
1.03
6.31

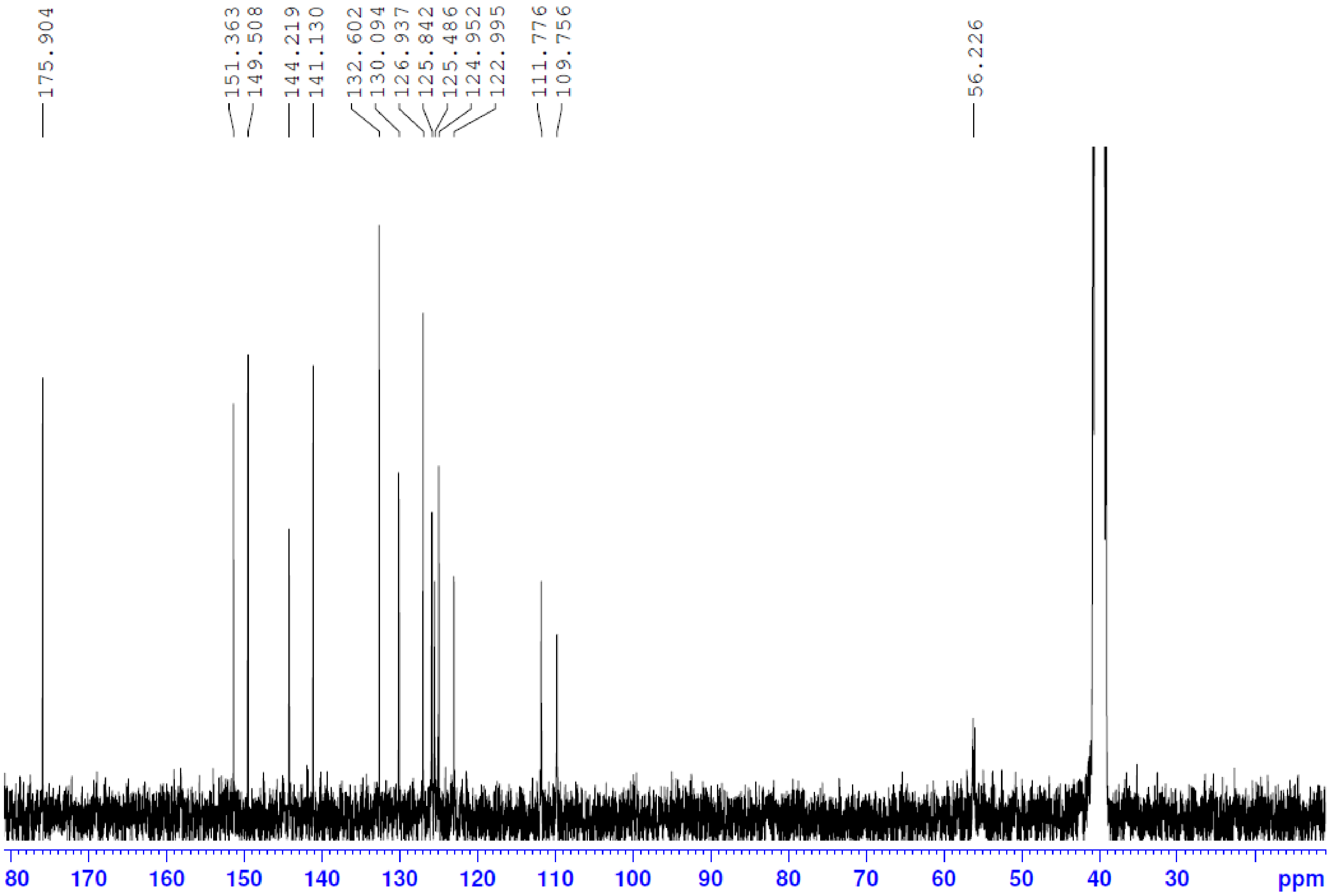
175.904
151.363
149.508
144.219
141.130
132.602
130.094
126.937
125.842
125.486
124.952
122.995
111.776
109.756
56.226
80
170
160
150
140
130
120
110
100
90
80
70
60
50
40
30
ppm

T15

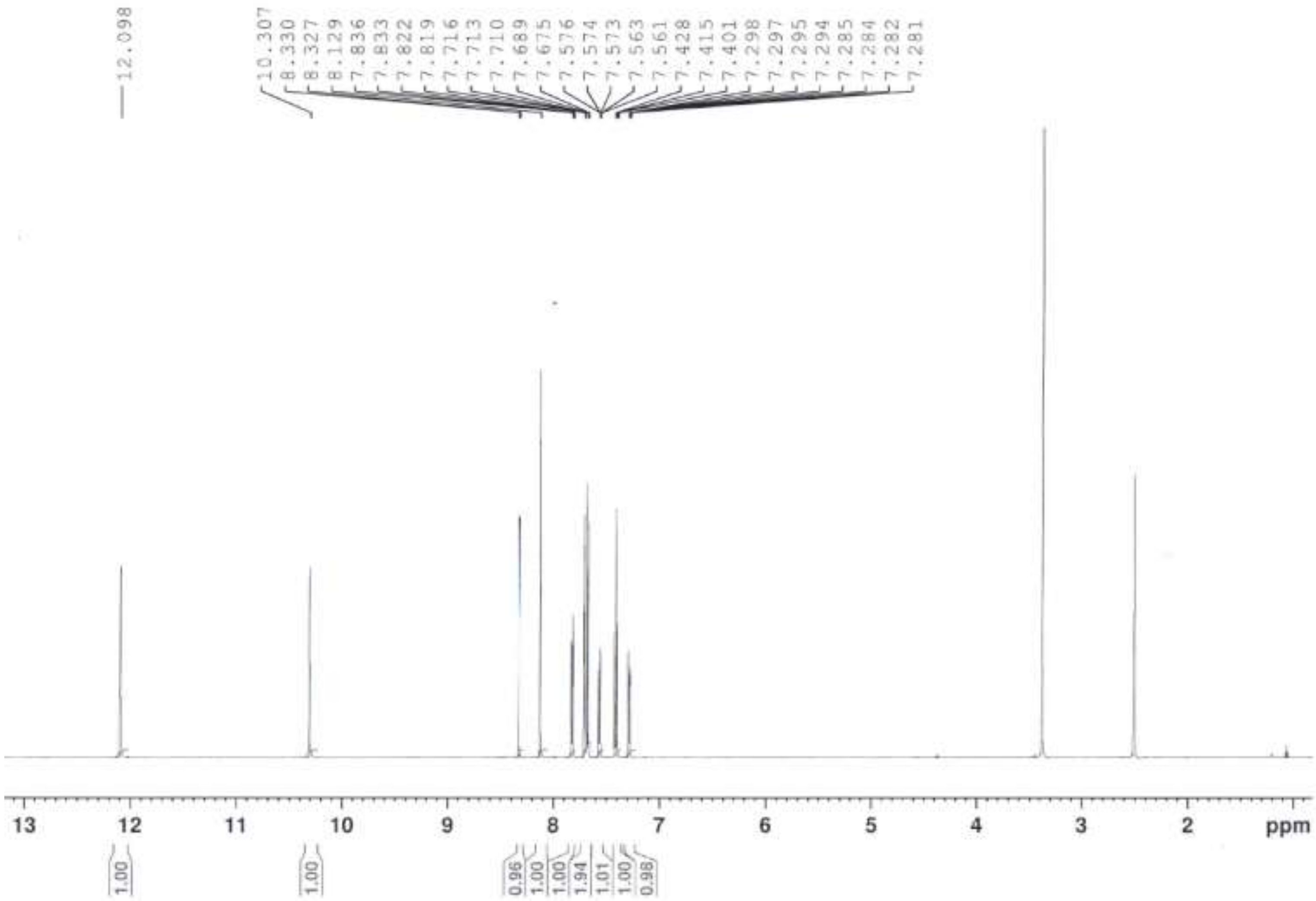
12.098
10.307
8.330
8.327
8.129
7.836
7.833
7.822
7.819
7.716
7.713
7.710
7.689
7.675
7.576
7.574
7.573
7.563
7.561
7.428
7.415
7.401
7.298
7.297
7.295
7.294
7.285
7.284
7.282
7.281
13 12 11 10 9 8 7 6 5 4 3 2 ppm
1.00
1.00
0.96
1.00
1.00
1.94
1.01
1.00
0.98

T16

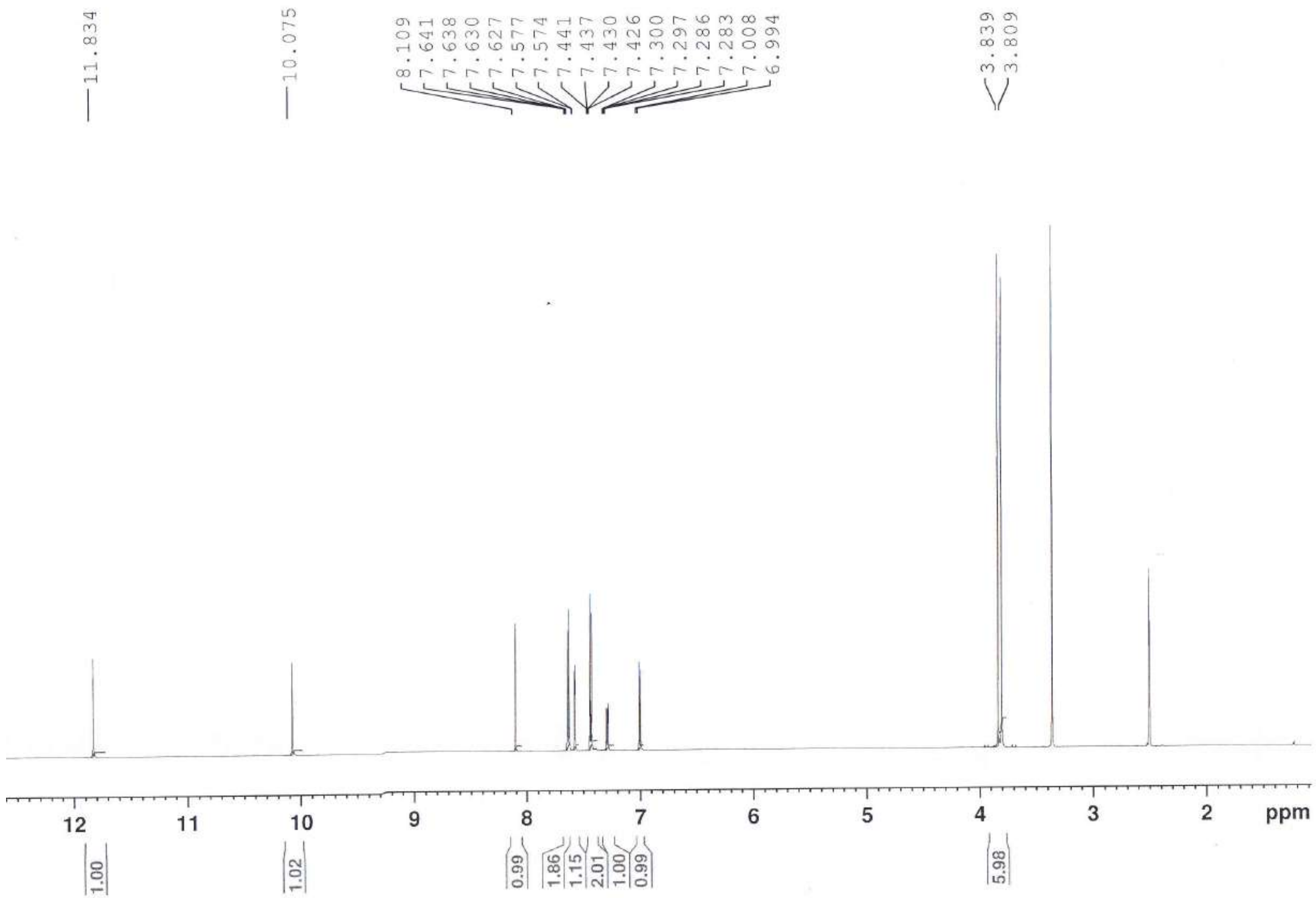
11.834
10.075
8.109
7.641
7.638
7.630
7.627
7.577
7.574
7.441
7.437
7.430
7.426
7.300
7.297
7.286
7.283
7.008
6.994
3.839
3.809
12
11
10
9
8
7
6
5
4
3
2
ppm
1.00
1.02
0.99
1.86
1.15
2.01
1.00
0.99
5.98

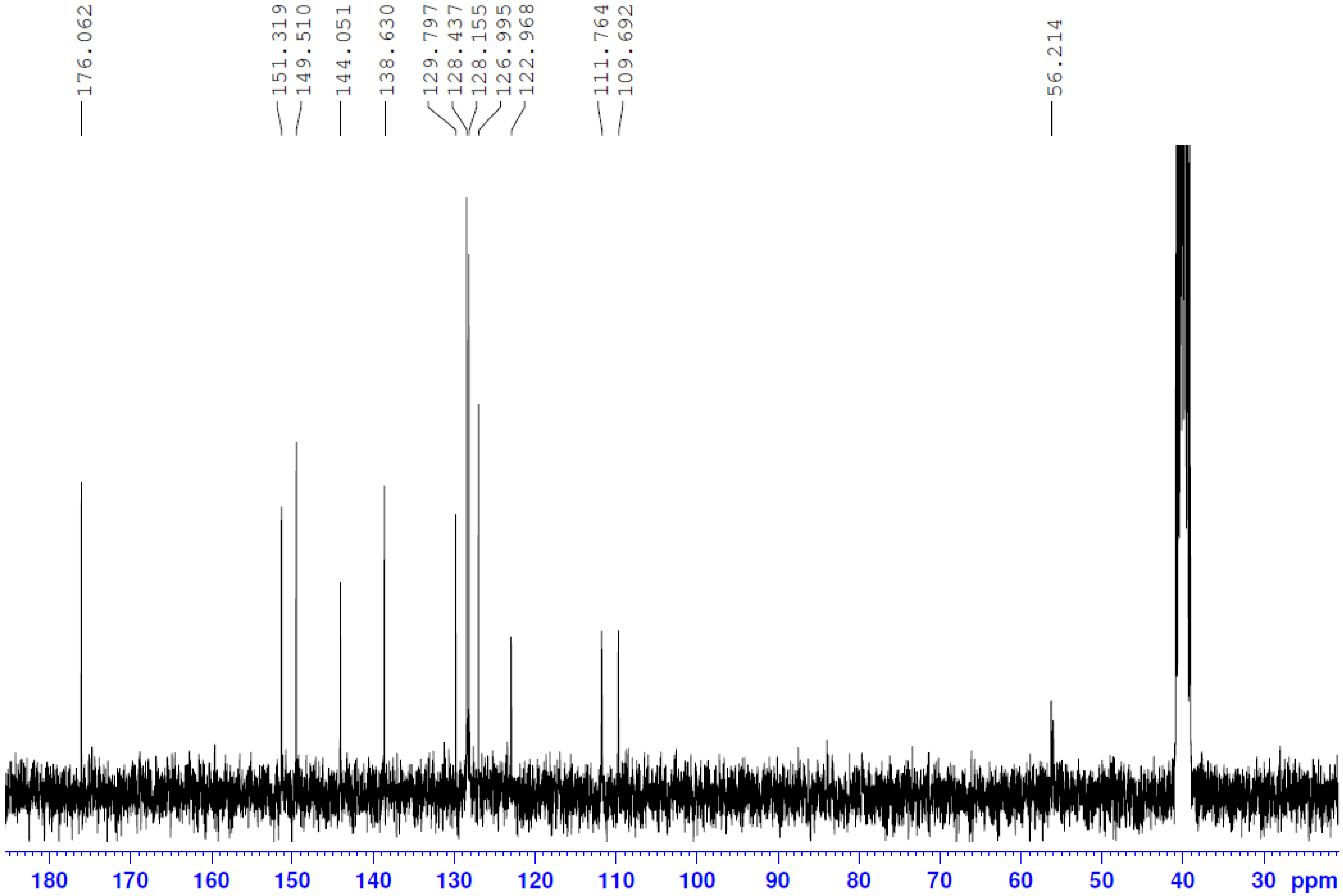

176.062
151.319
149.510
144.051
138.630
129.797
128.437
128.155
126.995
122.968
111.764
109.692
56.214
180
170
160
150
140
130
120
110
100
90
80
70
60
50
40
30
ppm

T17

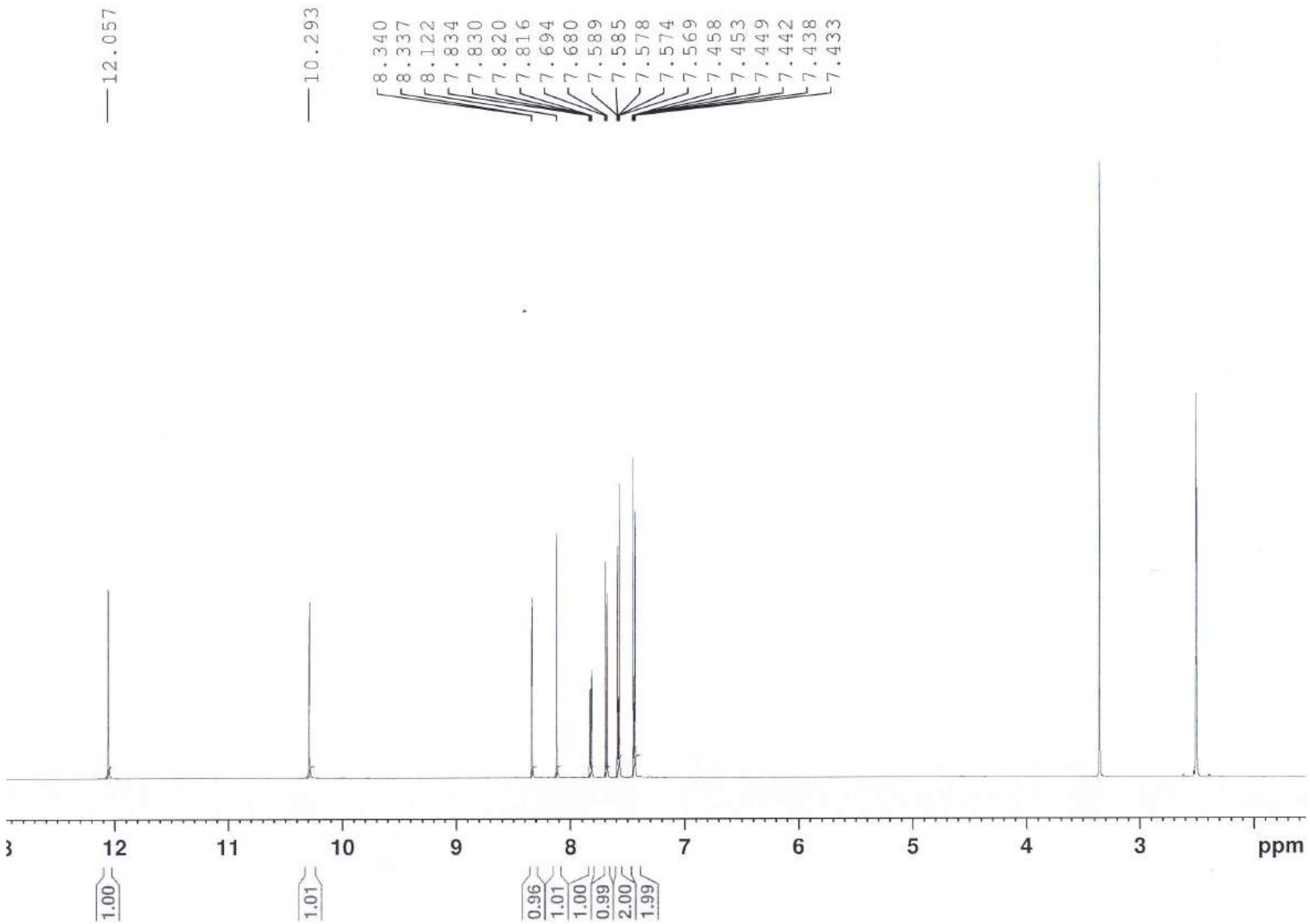

12.057
10.293
8.340
8.337
8.122
7.834
7.830
7.820
7.816
7.694
7.680
7.589
7.585
7.578
7.574
7.569
7.458
7.453
7.449
7.442
7.438
7.433
12
11
10
9
8
7
6
5
4
3
ppm
1.00
1.01
0.96
1.01
1.00
0.99
2.00
1.99

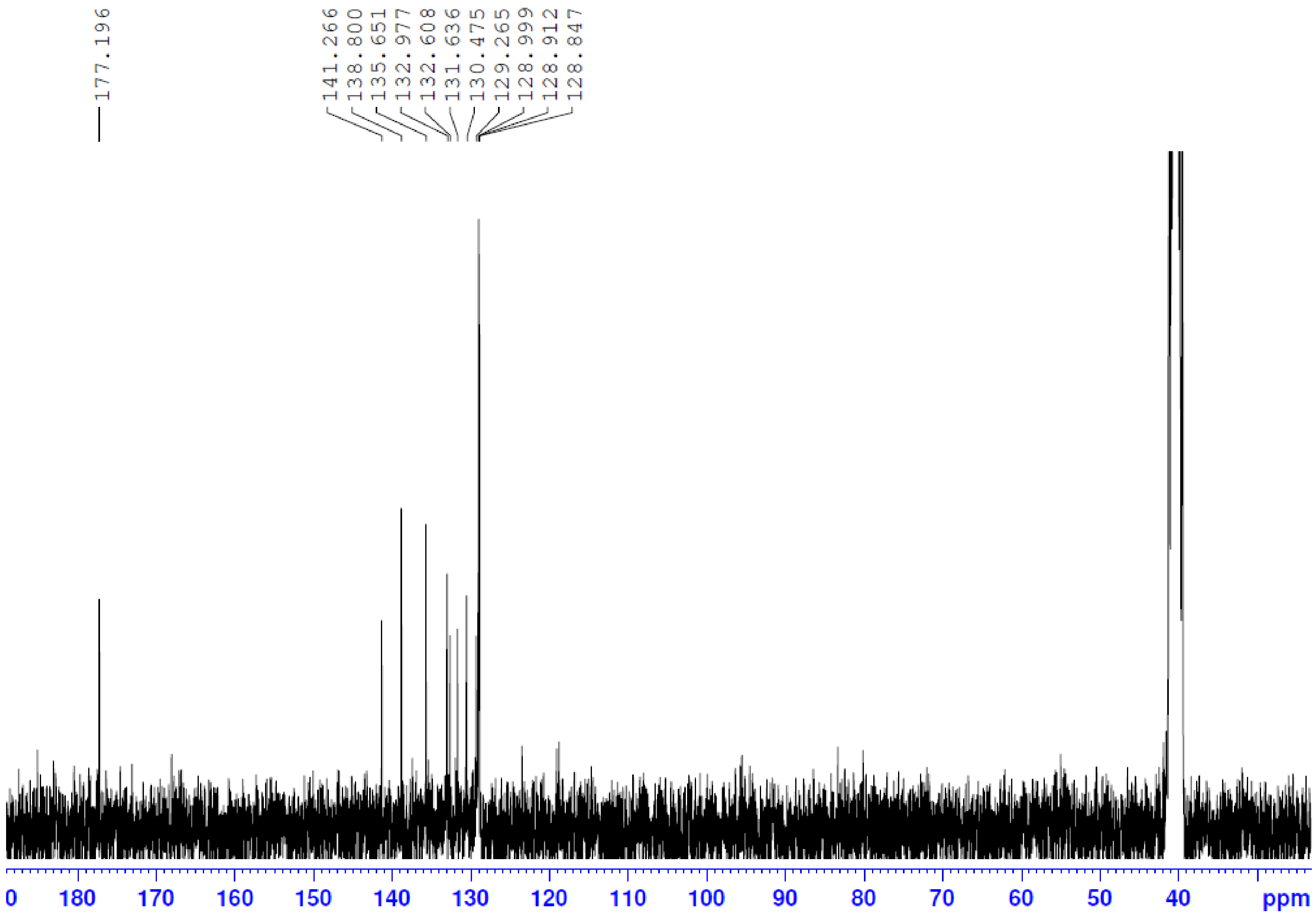
177.196
141.266
138.800
135.651
132.977
132.608
131.636
130.475
129.265
128.999
128.912
128.847
0
180
170
160
150
140
130
120
110
100
90
80
70
60
50
40
ppm

T18

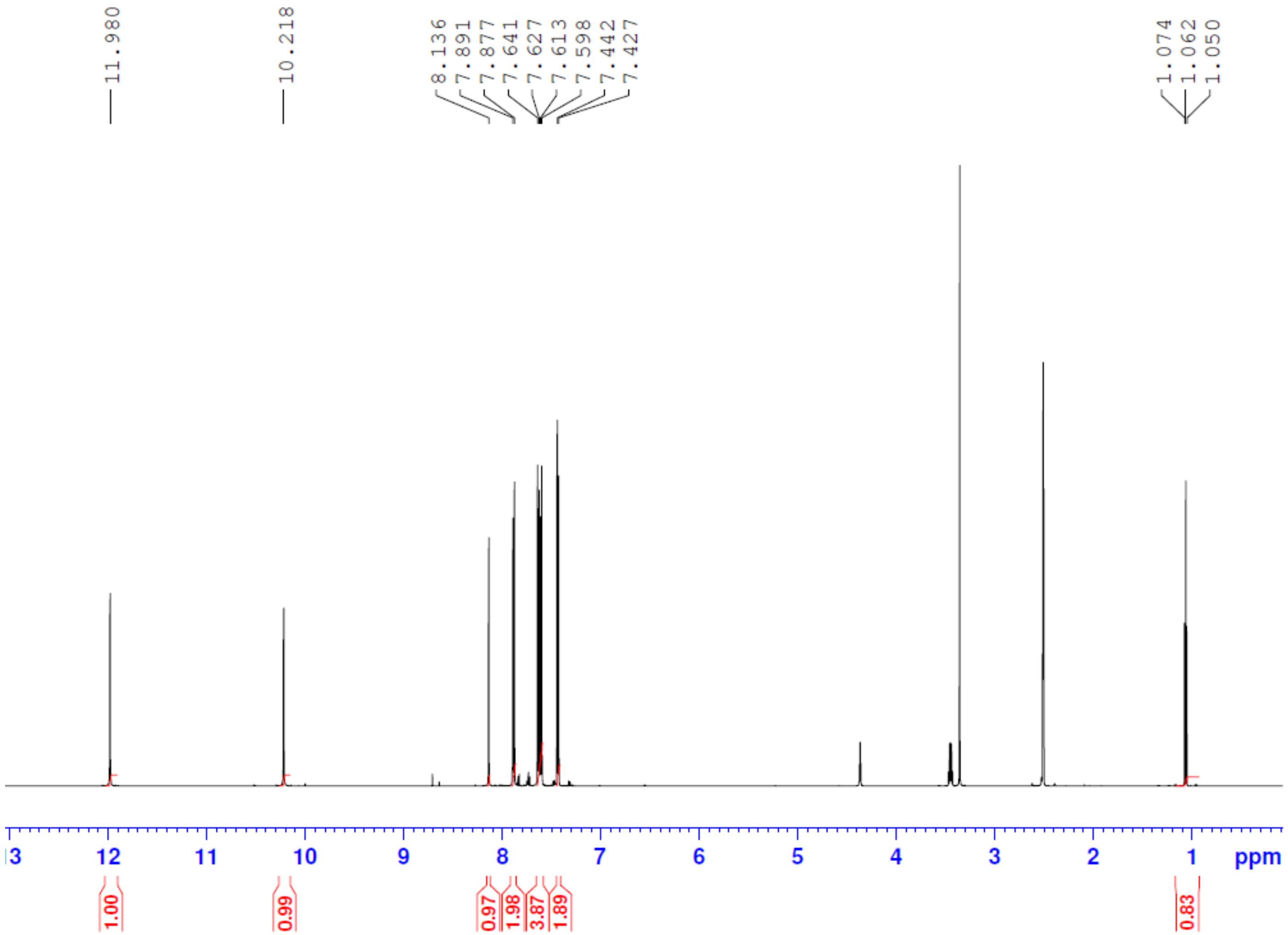
11.980
10.218
8.136
7.891
7.877
7.641
7.627
7.613
7.598
7.442
7.427
1.074
1.062
1.050
13
12
11
10
9
8
7
6
5
4
3
2
1
ppm
1.00
0.99
0.97
1.98
3.87
1.89
0.83

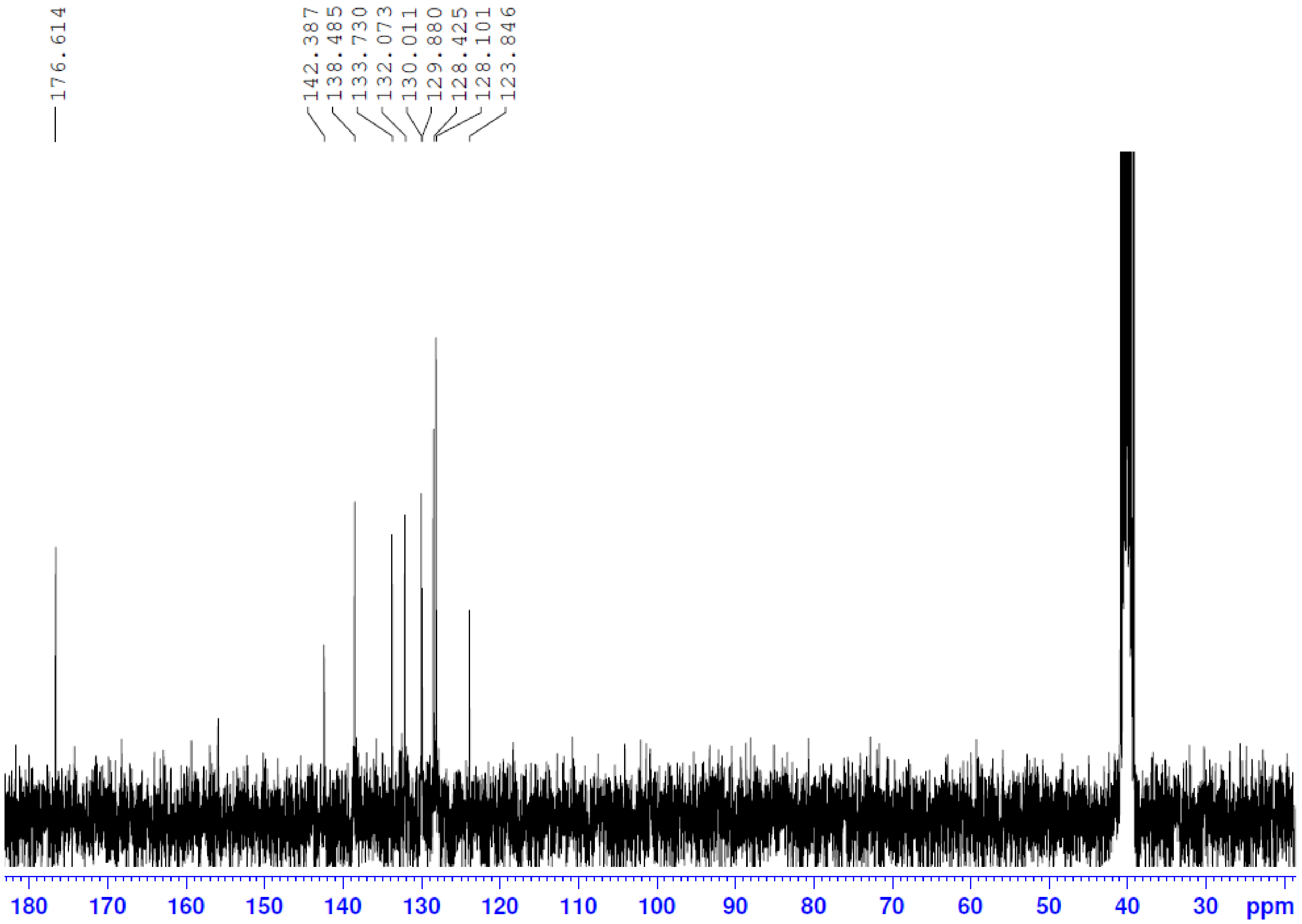
176.614
142.387
138.485
133.730
132.073
130.011
129.880
128.425
128.101
123.846
180
170
160
150
140
130
120
110
100
90
80
70
60
50
40
30
ppm

T19

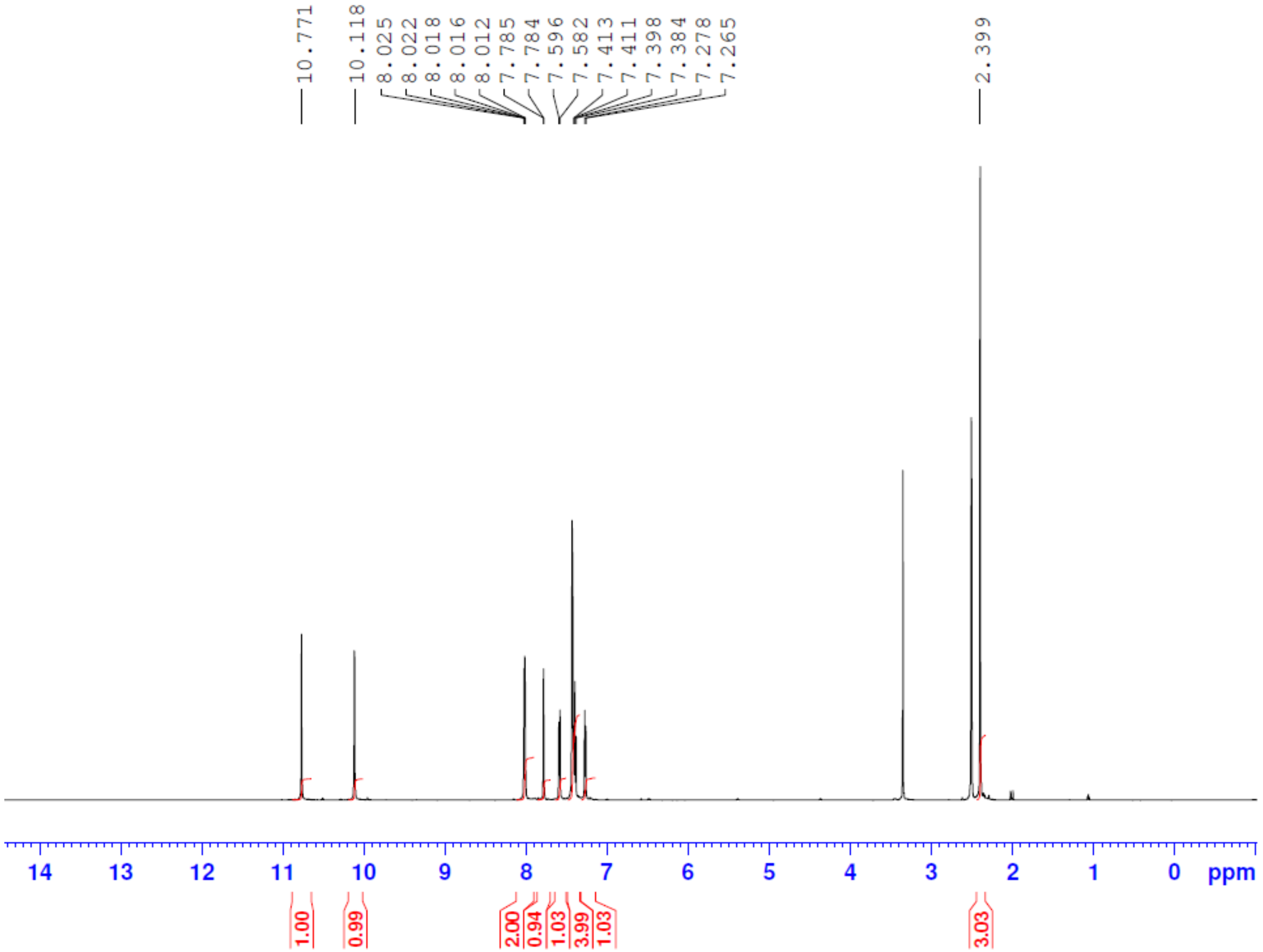
10.771
10.118
8.025
8.022
8.018
8.016
8.012
7.785
7.784
7.596
7.582
7.413
7.411
7.398
7.384
7.278
7.265
2.399
14
13
12
11
10
9
8
7
6
5
4
3
2
1
0
ppm
1.00
0.99
2.00
0.94
1.03
3.99
1.03
3.03
